# Emergence, prevalence, and evolution of H5N8 avian influenza viruses in central China, 2020

**DOI:** 10.1080/22221751.2021.2011622

**Published:** 2021-12-22

**Authors:** Xiang Li, Xinru Lv, Yi Li, Linhong Xie, Peng Peng, Qing An, Tian Fu, Siyuan Qin, Yuan Cui, Chengbo Zhang, Rongxiu Qin, Fengyi Qu, Zhenliang Zhao, Meixi Wang, Qiuzi Xu, Yong Li, Guoxiang Yang, Guang Chen, Jun Zhang, Hesong Zheng, Enda Ma, Ruifang Zhou, Xiangwei Zeng, Yulong Wang, Zhijun Hou, Yajun Wang, Dong Chu, Yanbing Li, Hongliang Chai

**Affiliations:** a College of Wildlife and Protected Area, Northeast Forestry University, Harbin, People’s Republic of China; b National Forestry and Grassland Administration, General Station for Surveillance of Wildlife Disease & Wildlife Borne Diseases, Shenyang, People’s Republic of China; c Sanmenxia Administration of the National Nature Reserve of the Yellow River Wetland, Sanmenxia, People’s Republic of China; d Ordos Forestry and Grassland Administration, Ordos, People’s Republic of China; e Research and Development Center, Hubei Wildlife Rescue, Wuhan, People’s Republic of China; f Bayannur Forestry and Grassland Administration, Bayannur, People’s Republic of China; g State Key Laboratory of Veterinary Biotechnology, Chinese Academy of Agricultural Sciences, Harbin Veterinary Research Institute, Harbin, People’s Republic of China

**Keywords:** Avian influenza viruses, China, H5N8, swan, 2.3.4.4b

## Abstract

Highly pathogenic influenza A(H5N8) viruses have caused several worldwide outbreaks in birds and are able cross the species barrier to infect humans, posing a substantial threat to public health. After the first detection of H5N8 viruses in deceased swans in Inner Mongolia, we performed early warning and active monitoring along swan migration routes in central China. We isolated and sequenced 42 avian influenza viruses, including 40 H5N8 viruses, 1 H5N2 virus, and 1 H9N2 virus, in central China. Our H5N8 viruses isolated in swan stopover sites and wintering grounds showed high nucleotide homologies in the whole genome, revealing a common evolutionary source. Phylogenetic analysis revealed that the H5 viruses of clade 2.3.4.4b prevalent in 2020 have further diverged into two sub-clades: b1 and b2. The phylogeographic analysis also showed that the viruses of sub-clade b2 most likely originated from poultry in Russia. Notably, whooper swans were found to be responsible for the introduction of sub-clade b2 viruses in central China; whooper and tundra swans play a role in viral spread in the Yellow River Basin and the Yangtze River Basin, respectively. Our findings highlight swans as an indicator species for transborder spreading and monitoring of the H5N8 virus.

## Introduction

Influenza A viruses (IAVs) infect a broad spectrum of hosts, including wild birds, poultry, swine, horses, seals, cats, and humans, occasionally causing major outbreaks or severe disease. Wild birds, especially those of the order *Anseriformes* and *Charadriiformes*, are considered to be the natural reservoirs of IAVs and play a fundamental role in the spread and maintenance of the virus [1]. Moreover, migratory birds contribute to the wide geographic spread and distribution of the circulating highly pathogenic avian influenza (HPAI) H5 virus [2]. In 2014, HPAI H5N8 virus of clade 2.3.4.4 was first identified in South Korea and subsequently spread to Asia, Europe, and North America by migratory waterfowl [3–5]. Another HPAI H5N8 virus of clade 2.3.4.4 began its intercontinental spread in the autumn in 2016 [6], and it has since been shown to have a sustained prevalence in Europe, Africa, and the Middle East (OIE, https://www.oie.int/en/animal-health-in-the-world/). In early 2020, after small H5N8 outbreaks were reported in poultry in Europe, HPAI H5N8 viruses were continuously detected in Iraq, Russia, and Kazakhstan beginning in May [7]. Moreover, seven poultry farm workers infected with a clade 2.3.4.4b H5N8 virus in Russia were first confirmed in December 2020 [8]. Thus, the global spread of the HPAI H5N8 virus has become a major concern to poultry farming, wildlife conservation and security, as well as global public health [9]. The most challenging tasks associated with HPAI disease ecology is to predict and prevent viral spread, transmission pathways, and host-specific contributions to interregional spread.

Migratory birds moving within several flyways in Eurasia exhibit an overlap in the locations of circumpolar arctic and subarctic breeding regions [10]. Each year, these birds typically perform seasonal movements between breeding and wintering grounds, viral interactions could also occur among different populations and species, during migration or within habitats. Long-distance migration also exposes the domestic or resident bird populations to IAVs at multiple stopover sites. Therefore, understanding the long-distance movement of wild migratory birds among breeding, wintering grounds, and stopover sites is crucial for explaining the interregional spread of HPAI viruses. Among the bird species involved in the HPAI H5 epidemic, swans appear to be the frequently affected wild species [11, 12]. Frequent HPAI H5 virus detection in swans might be related to their unique biological characteristics: (a) white feathers and large size make them easy to find; (b) concentrated habitat distribution; (c) highly susceptible to infection with the HPAI H5 virus; and (d) efficient spreaders of the virus based on duration, route, and concentration of viral excretion [11, 13].

Whooper swans (*Cygnus cygnus*) breed in northern Eurasia and winter in Europe and eastern Asia (i.e. China, South Korea, and Japan). In the Central Asian flyway, they breed in central-northern and western Mongolia, winter in the Sanmenxia Reservoir area in China, and migrate along the Yellow River and its tributaries of Inner Mongolia, Gansu, Ningxia, Shaanxi, and Shanxi [14]. For the tundra swan (*Cygnus columbianus*), the largest flyway population is the Eastern population, which breeds in the Russian arctic and winters in the middle and lower reaches of the Yangtze River in China [15].

It has become of increasingly apparent that as HPAI H5 virus-susceptible birds, swans might play a key role in the geographic spread of virus [14, 16]. Here, we report the monitoring and early warning of the clade 2.3.4.4b H5 virus along the swan migration routes in central China during Autumn 2020.

## Materials and methods

### Eggs

Ten-day-old specific pathogen-free chicken embryos were obtained from the National Poultry Laboratory Animal Resource Centre, Harbin Veterinary Research Institute, Chinese Academy of Agriculture Sciences, Harbin 150069, China.

### Sample collection and virus isolation

Sampling was conducted between October and November 2020 along swan flyways in central China. A total of 1,075 samples were collected, of which 872 samples were collected from the Yellow River Valley (Inner Mongolia, n = 189; Shaanxi, n = 108; Shanxi, n = 399; and Henan, n = 176), and 203 were collected from the Yangzi River Valley (Hubei). Swabs or tissue samples were collected from diseased or dead birds found in the field. Fresh and wet faecal samples were collected from the bird’s habitat in the morning. All samples were placed into viral transport media, stored in a handheld portable 4°C refrigerator, transported to the laboratory within 24 h, and stored at −80°C immediately for future use. Avian influenza viruses were isolated in 10-day-old specific pathogen-free chicken embryos using viral isolation procedures in accordance with the method described in the WHO manual [17]. The host species of the droppings was determined using the methods as previously described [18, 19].

### Subtyping and sequencing

HA subtypes were initially identified using a hemagglutination inhibition test. Viral RNA was extracted from haemagglutinin-positive incubated allantoic fluid samples using a QIAamp Viral RNA Mini Kit (Qiagen, Germany), reverse transcribed using the influenza A-specific primer, Un12, and subjected to RT–PCR using the method described in the WHO manual [17] to further confirm AIV-positivity. The PCR products of eight fragments of the isolates were sequenced using a set of specific sequencing primers listed in a previous dissertation [20]. The sequencing data were compiled using the SeqMan programme (DNASTAR, Madison, WI, United States).

### Genetic analysis

A Basic Local Alignment Search Tool (BLAST) search was performed against the sequencing data in the Global Initiative on Sharing All Influenza Data databases (GISAID; https://www.gisaid.org) to identify and download the closest relatives of our isolates. The top 100 BLAST hits were derived for each gene dataset. All reference sequences were downloaded in April 2021. The sequences were aligned using MAFFT [21] implemented in PhyloSuite 1.21 [22]. The alignment lengths for each dataset were: PB2: 2,277 nucleotides (nt); PB1: 2,271 nt; PA: 2,148 nt; H5: 1,701 nt; H9: 1,680 nt; NP: 1,494 nt; N8: 1,410 nt; N2: 1,407 nt; M: 979 nt; and NS: 835 nt. Repetitive and short sequences were removed for each dataset. The H5 phylogenetic analyses were conducted with an integrated dataset that was comprised of our isolates, sequence data downloaded in the BLAST search, and the 2017–2020 global H5N8 viruses. Additional sequences of the 2020 H5N8 viruses were also downloaded from GenBank (https://www.ncbi.nlm.nih.gov/genbank/). A phylogenetic analysis of H9 and N2 was conducted using our isolate and the sequences derived from the BLAST search. Phylogenetic analyses of the remaining datasets were reconstructed with the inclusion of our isolates, the sequence data downloaded in the BLAST search, and the 2020 global H5N8 viruses. Maximum likelihood (ML) phylogenies were inferred using IQ-TREE [23] under the best-fit substitution model for 10000 ultrafast bootstraps [24]. The best-fit substitution model was selected using the Bayesian information criterion of ModelFinder [25] implemented in PhyloSuite 1.21 [22].

### Phylodynamic analysis

To investigate the transmission patterns of the sub-clade 2.3.4.4b2 H5N8 viruses, we performed a phylogeographic analysis using an asymmetric model, with Bayesian stochastic search variable selection implemented in BEAST version 1.10.4 [26]. We first confirmed that the sequences contained sufficient temporal signal by using TempEst (version 1.5.3, http://tree.bio.ed.ac.uk/) as described previously [27]. The best-fit substitution model was selected as described above. We computed the marginal likelihoods using path sampling and stepping-stone sampling [28] to compare two models of among-lineage rate variation (strict clock and uncorrelated lognormal relaxed clock) and three coalescent-based tree priors (constant size, exponential growth, and Bayesian skyline). The Bayes factors revealed support for the Bayesian skyline tree prior and uncorrelated lognormal relaxed clock model. Three independent MCMC analyses were run, each for 100 million iterations and sampling every 10 thousand iterations. All runs were performed via the CIPRES Science Gateway (https://www.phylo.org). Tracer version 1.7.1 [29] was used to check for sufficient sampling and convergence among the three sets of samples after discarding the first 10% of the samples as burn-in, and all parameters reached an adequate effective sample size (exceeding 200). The maximum clade credibility tree was computed in TreeAnnotator, a part of the BEAST package. The resulting diffusion rates were used to calculate the Bayes factors using SpreaD3 [30]. Significant migration pathways were determined based on the combination of a BF of ≥ 3 and mean indicator of ≥ 0.5: decisively supported diffusions with BF ≥ 1000, very strongly supported diffusion with 100 ≤ BF < 1000, strongly supported diffusions with 10 ≤ BF < 100, and supported diffusions with 3 ≤ BF < 10.

## Results

### The emergence and monitoring of HPAI H5

On 17 October 2020, two HPAI H5N8 viruses, classified as clade 2.3.4.4b, were detected for the first time in two dead swans, whooper swan (*Cygnus cygnus*) and mute swan (*Cygnus olor*) in Inner Mongolia, China [31]. Subsequently, intensified surveillance of HPAI H5 virus was conducted along the reported whooper swan migration routes [14]. Moreover, a total of 42 AIVs were isolated from wild birds in their habitats in central China during autumn of 2020 (Appendix Table 1). In addition to the initial two viruses in Inner Mongolia [31], four H5N8 viruses were detected in the key stopover sites of whooper swan in Shaanxi (Hekou Reservoir and Hongjian Nur) from November 9 to10; 12 H5N8, 1 H5N2, and 1 H9N2 viruses were detected in Shanxi (Shengtian Lake and Sanwan wetland) on November 10; and 13 H5N8 viruses were detected in Henan (Sanmenxia Reservoir Area) from November 2–11; nine HPAI H5N8 viruses were also isolated in Longan Lake in Hubei, which is primarily the wintering ground of tundra swan, from November 4–16.

### Phylogenetic analysis

The whole genomic sequences of 42 AIVs, including 40 H5N8 viruses (2020–H5N8), 1 H5N2 virus, and 1 H9N2 virus, isolated in central China in autumn of 2020 were obtained (Appendix Table 2). In addition to a single H5N8 virus which was isolated from Eurasian eagle-owl (*Bubo bubo*), 41 AIVs were isolated from migratory waterfowl: whooper swan (n = 30), tundra swan (n = 7), mute swan (n = 1), common teal (*Anas crecca*, n = 1), bean goose (*Anser fabalis*, n = 1), and whiskered tern (*Chlidonias hybrida*, n = 1). The sequences of all isolates were deposited in to the GISAID (Appendix Table 2). All of the isolates were analyzed along with that of the H5N8 viruses isolated in 2020 and other closely related sequences.

The 2020-H5N8 sequences shared high nucleotide identity across all eight gene segments (99.2% – 100%) and were identified as HPAIVs based on the amino acid sequence REKRRKR↓GLFGAI at the haemagglutinin (HA) cleavage site. This finding indicates that they are descendants of a common ancestral virus. The H5N2 isolate, A/whooper swan/Shanxi/SX116/2020(H5N2) (SX116–H5N2), was found to share the same HA cleavage site with 2020-H5N8, but exhibited a diverse origin as low homology was observed in seven of the genes (91.7% – 96.9%, except for the NA gene). Interestingly, SX116-H5N2 shared quite a high nucleotide identity (99.9% – 100%) with the H9N2 isolate, A/whooper swan/Shanxi/SX126/2020(H9N2) (SX126-H9N2), in six of the genes (PB2, PB1, PA, NP, NA, and M), revealing potential recombination events.

**Figure 1.**
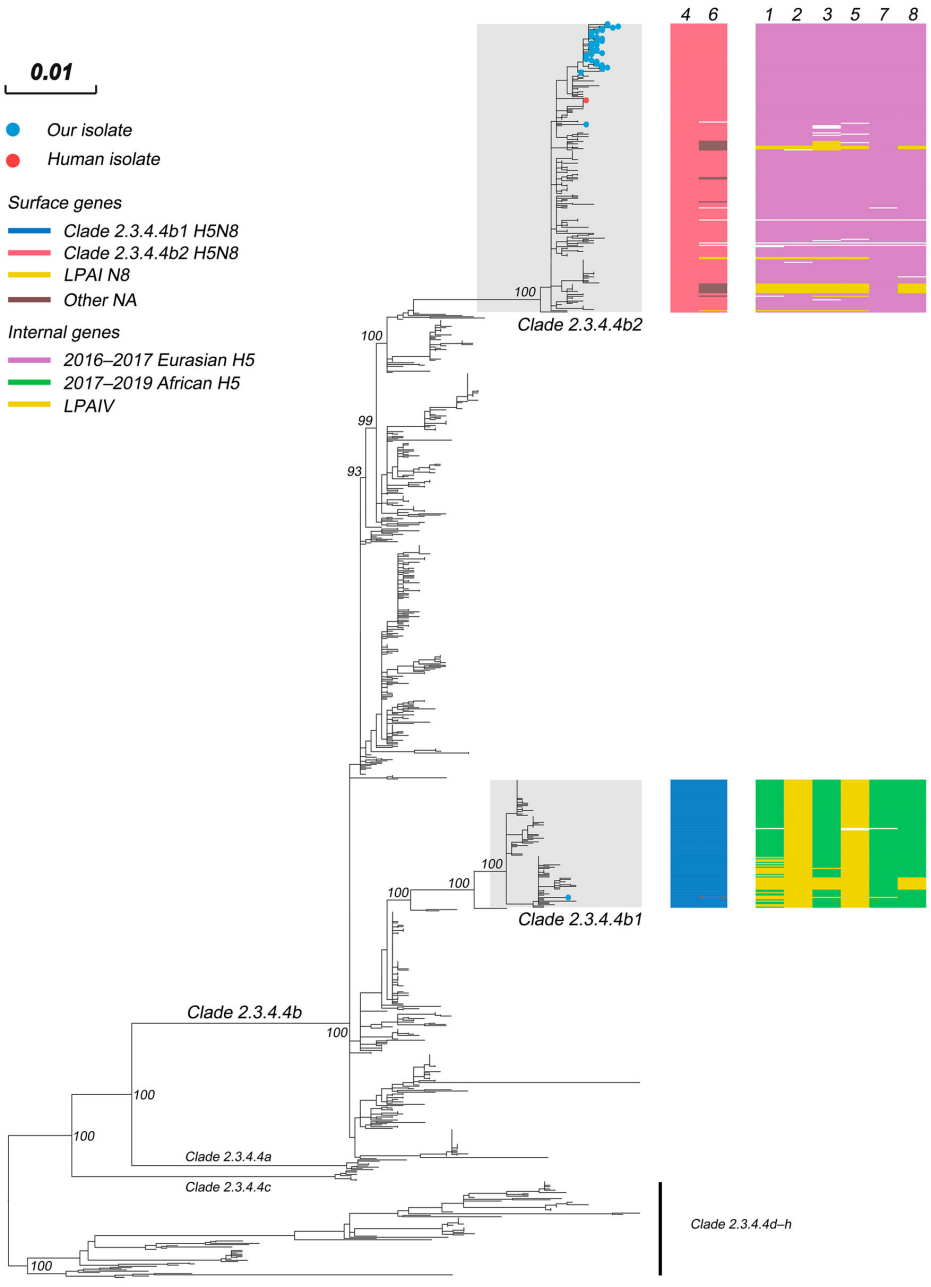
Maximum-likelihood phylogenetic tree of the HA gene segment of HPAI H5 sequences. Our isolates and the Russian human strain are marked by blue and red coloured circles, respectively. For the clade 2.3.4.4b H5 virus isolated in 2020, the clade origins of each gene segment are indicated by different coloured bars. A UFBoot support values of the major branch are indicated. The scale bar represents the nucleotide substitutions per site. Detailed information is available in Supplementary Materials. See also Appendix Figure 1 and Appendix Table 7.

In the Maximum-likelihood (ML) phylogenies of the H5 gene, 2020-H5N8 (n = 40) and SX116-H5N2 were both classified in 2.3.4.4b, and were further diverged into two phylogenetically distinct sub-clades: 2.3.4.4b1 and 2.3.4.4b2 (Figure 1) [7, 32, 33]. The high bootstrap value also supported the two novel sub-clades. Isolate SX116-H5N2 was divided into sub-clade 2.3.4.4b1 which was primarily detected in European poultry during early 2020, the immediate outlier virus was A/guinea fowl/Nigeria/OGGF11T_19VIR8424–7/2019(H5N8). The viruses of sub-clade 2.3.4.4b1 eventually spread to wild waterfowl in Asia in the autumn of 2020 (i.e. China, Japan [34], and Korea [35]); In May 2020, the sub-clade 2.3.4.4b2 was first emerged in Iraq and followed by the detection of H5N8 in southern central Russia, Kazakhstan, Netherlands, and other European countries. All of our H5N8 isolates among the H5 viruses isolated in Eurasian in autumn of 2020 were clustered into sub-clade 2.3.4.4b2, of which the ancestral strains were widely circulated in Eurasia in 2017 and 2018.

The phylogenetic analysis also demonstrated that 2020-H5N8 tends to cluster in the complete genome, indicating a common evolutionary source (Appendix Figure 1). In contrast, in the ML trees, SX116-H5N2 further confirmed a process of genetic recombination in which only HA and NS genes were originated from the common ancestor of 2.3.4.4b1. The NA gene of SX116-H5N2 and SX126-H9N2 were closely related to those of low pathogenic avian influenza (LPAI) HxN2 viruses established in wild ducks in East Asia. In the PB2, PB1, PA, NP, and M phylogenies (Appendix Figure 1), SX116-H5N2 and SX126-H9N2 shared most recent common ancestor, which was derived from the Mongolia poultry LPAI virus gene pool. The NS gene of SX126-H9N2 was also derived from the LPAI virus gene pool of Mongolia poultry. The HA gene of SX126-H9N2 was clustered with the 2016/2018 viruses of waterfowl-origin in East Asia and the long branch lengths for SX126-H9N2 suggested that the virus had been circulating undetected for the intervening period.

### Phylodynamic analysis

**Figure 2.**
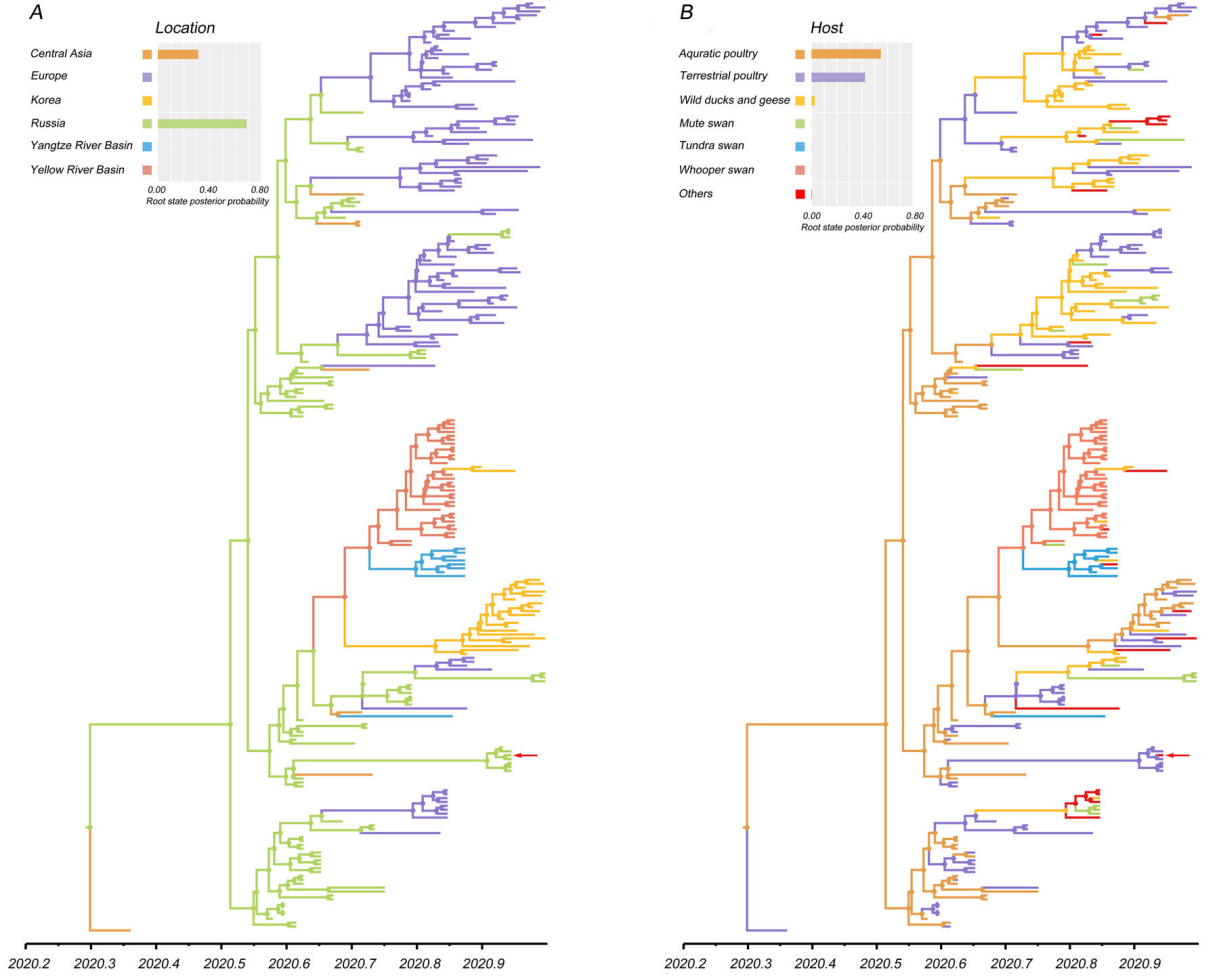
Maximum clade credibility (MCC) time-scaled phylogenetic tree of HA sequences of sub-clade 2.3.4.4b2 H5 virus coloured by geographic location (A), and host type (B). The branches are coloured according to the most probable ancestral geographic location, and host type. The Russian human strain is denoted by red arrows. The root state posterior probabilities are shown in each inset panel.

**Figure 3.**
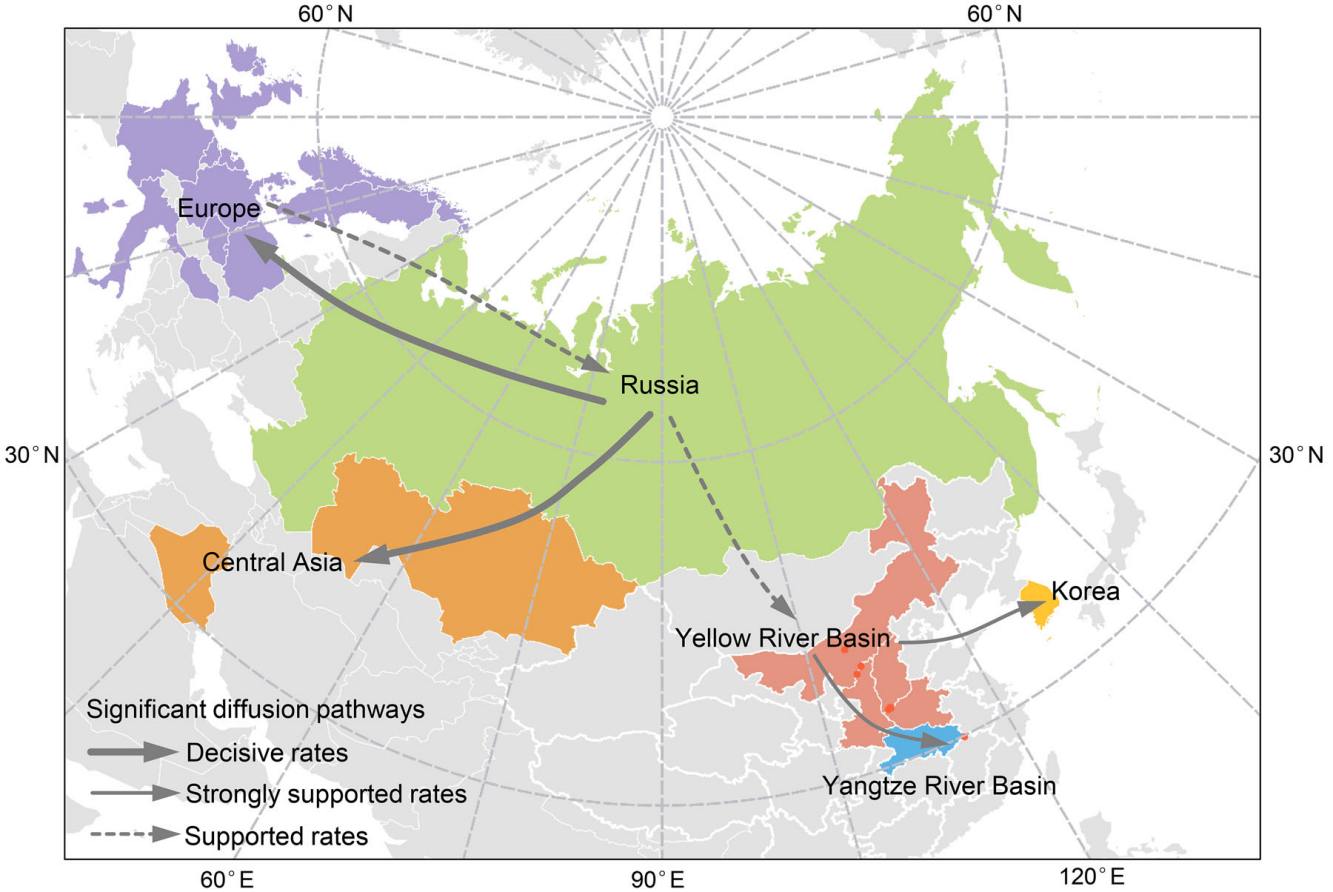
Spatial diffusion of the HA gene segment of the sub-clade 2.3.4.4b2 H5 virus. The red dots indicate the sampling site in central China. The bold grey arrow indicates decisively supported diffusions (BF ≥ 1000); solid grey arrows, strongly supported diffusions (10 ≤ BF < 100); and dashed grey arrows, supported diffusions (3 ≤ BF < 10).

To gain further insight into the large-scale transmission patterns of the sub-clade 2.3.4.4b2 H5N8 virus in Eurasia during autumn of 2020, and to clarify the contribution of different bird host populations to viral transmission, we performed a phylogeographic analysis of the HA gene sequences. We grouped the isolates into six distinct geographic categories: Central Asia (Iraq and Kazakhstan), Europe, Korea, Russia, Yangzi River Basin (Hubei, China), and the Yellow River Basin (Inner Mongolia, Shaanxi, Shanxi, and Henan, China). The AIV hosts were categorized into seven groups: aquatic poultry, terrestrial poultry, mute swan, tundra swan, whooper swan, wild ducks and geese (except mute swan, tundra swan, and whooper swan), others (Appendix Table 3). Our analyses support the idea that Russia was the geographic origin of sub-clade 2.3.4.4b2, with a posterior probability of 0.68 (Figure 2A). Phylogeographic analysis indicates that there are six migration pathways in the spatial diffusion, with three originating from Russia to central Asia (Bayes factor, BF > 1,000), Europe (BF > 1,000), and the Yellow River Basin (BF = 8.64), two from the Yellow River Basin to the Yangtze River Basin (BF = 26.05) and Korea (BF = 42.46), and the sixth from Europe to Russia (BF = 6.28) (Figure 3; Appendix Table 4). As such, our analysis indicates that Russia acted as a primary seeding population while the Yellow River Basin established epidemiological links with other Asian geographical locations in this transmission network.

The most likely ancestral host population for sub-clade 2.3.4.4b2 is poultry, with a posterior probability of 0.97 (aquatic poultry, posterior probability = 0.55; terrestrial poultry, posterior probability = 0.42) (Figure 2B). Poultry acted as a viral source while several statistically significant transitions (BF ≥ 3) to other host populations were also supported (Appendix Table 5). One diffusion pathway with decisive support of BF > 1,000 was recognized as originating from aquatic poultry to terrestrial poultry. A complex host history with a large amount of viral exchange was also indicated by several very strongly (100 ≤ BF < 1000) and strongly (10 ≤ BF < 100) supported diffusion pathways (Appendix Table 5). Our analysis reflected this dynamic with three species of swan playing different roles in spreading HPAI H5 virus: mute swan acted as a receiver, with only one statistically significant transition from wild ducks and geese (BF = 36.08); whooper swan acquired the virus from aquatic poultry (BF = 5.41), and subsequently transmitted it to tundra swan (BF = 12.26), wild ducks, and geese (BF = 76.07), and other hosts (BF = 13.28). Therefore, whooper swan acted as the seeding population in viral dissemination.

### Molecular characterization

In the H5 HA protein, the amino acid substitutions Q222L [36] and G224S [36] were not found; however, the S133A, S155N, T156A mutations [37, 38] were identified in all H5 isolates; T188I [37], V210I [39] mutations were found in partial H5 isolates, suggesting that these viruses may be able to potentially bind to human-type receptors (α−2,6 galactose sialic acids) (Appendix Table 6). Residues Q591 [40], E627 [41], and D701 [40] in the PB2 protein suggest that these viruses have not yet adapted to mammalian hosts. Notably, the D3 V [42] and D622G [43] mutations in the PB1 protein, N383D [44] mutation in the PA protein, and M105 V [45] and A184 K [46] mutations in the NP protein were observed in all isolates, which have been reported to be associated with increased polymerase activity or virulence. Furthermore, pathogenicity-associated mutations (N30D [47], I43M [48], and T215A [47] in the M1 protein), as well as L98F, I101M [49], and T215A [50] in the NS1 protein were observed, which might enhance viral virulence in mice. In addition, SX116-H5N2 exhibited the N200S mutation and PDZ motif, GSEV, in the NS1 protein and SX126-H9N2 displayed the PDZ motif, ESEV.

## Discussion

The re-emergence of H5N8 in Eurasia, especially its detection in human beings in Russia, poses a serious threat to public health. Recently, whooper swans have drawn increased attention as sentinel species in relation to HPAI H5 dissemination [14, 16]. Since swans in Russia have been reported to be infected with the HPAI H5N8 virus, we proposed an early warning system for HPAI outbreaks among whooper swan migratory routes in central China in accordance with the authenticated migration routes for swans [14, 15].

In the present study, we isolated 42 AIVs from wild birds along the Yellow River Basin (Inner Mongolia, Shaanxi, Shanxi, and Henan) and the Yangtze River Basin (Hubei), including 40 HPAI H5N8 viruses, 1 HPAI H5N2 virus (in Shanxi), and 1 LPAI H9N2 virus (in Shanxi). Our study conclusively demonstrated that the host distribution of the HPAI H5 virus was determined to be primarily in whooper swan in the Yellow River Basin (93.75%, 30/32) and in tundra swan in the Yangtze River Basin (77.78%, 7/9). The Yellow River Basin and the Yangtze River Basin constitute a complex wetland ecosystem in central China where the East Asian/Australasian and central Asian flyways overlap, providing a vital habitat for migratory waterfowl and playing an important intermediate role in connecting AIVs. A similar pattern was evident when compared to the prevalent of H5N8 viruses in 2016, as similar H5N8 viruses were detected in both in whooper swans in Shanxi (the Yellow River Basin) [51] and wild waterfowl in Hubei (the Yangtze River Basin) [52] in late 2016, following the outbreak of HPAI H5N8 in June 2016 in Ubsu-Nur Lake at the Russia-Mongolia border [53]. This finding suggests that HPAI H5N8 outbreaks in central China are closely associated with Russia.

Evolutionary genetic analyses indicate that the clade 2.3.4.4b H5 viruses detected in 2020 clustered into two distinct groups: viruses of sub-clade 2.3.4.4b1, which caused small outbreaks limited to the European poultry sector in early 2020, and spread to Asia by migratory waterfowl during autumn; and sub-clade 2.3.4.4b2, which was first detected in Iraq in May, and contained viruses primarily prevalent during autumn and winter of 2020 in Russia, Kazakhstan, Europe, and China. To further disentangle the host contributions in the spatial expansion of sub-clade 2.3.4.4b2 HPAI H5, we summarized the spatial and host dynamics. While we found that sub-clade 2.3.4.4b2 originated from Russian aquatic poultry, we could not exclude the possibility that viruses originated from central Asian terrestrial poultry due to the presence of a single isolate in chicken in Iraq.

Outbreaks of sub-clade 2.3.4.4b2 HPAI H5 in southern central Russia were reported in aquatic poultry in late July 2020, after which the virus subsequently circulated in various hosts, including aquatic poultry, terrestrial poultry, migratory waterfowl, and ultimately impacted poultry workers [8]. In autumn, the virus spread from Russia to Kazakhstan, Europe, and China, respectively. Moreover, the virus underwent two historical host shifts before it leaped to humans in Russia. This followed a trajectory of transmission from aquatic poultry to terrestrial poultry, and finally to farm workers (Figure 2B). Therefore, measures should be taken to govern the spill-over risk of HPAI H5 virus in the poultry trade and to protect people who are at risk from possible HPAI pandemics (i.e. via poultry vaccination, strengthened quarantine, and enhanced surveillance in hotspots or sentinel birds).

The analysis of the HA gene segment of sub-clade 2.3.4.4b2 suggests that the strains identified in Autumn of 2020 in China had most likely been introduced from Russia (Russia to the Yellow River Basin, BF = 7.67). Moreover, whooper swans are likely the most responsible for the initial introductions. After the detection of the H5N8 virus in the dead whooper swan in Inner Mongolia (the first port of entry for whooper swans in central China) in October 2020, we strengthened the active monitoring of HPAI H5N8 along the migration routes of swans in central China. Eventually, 40 HPAI H5N8 viruses which exhibited high nucleotide identity were isolated. In the Yellow River Basin (Inner Mongolia, Shaanxi, Shanxi, and Henan), all H5N8 isolates (n = 31) displayed high nucleotide identity at the genomic level and viruses were primarily isolated from whooper swan (28/31). Moreover, several cross-species transmission events from whooper swan to mute swan, common teal, and Eurasian eagle-owl were also observed. Another important cross-species transmission event from whooper swan to tundra swan (BF = 12.26) caused the spread of the virus to the Yangtze River Basin. As such, whooper swan should be considered the sentinel species in central China for monitoring the invasion of HPAI viruses. In contrast to whooper swans, which overwinter in entirely restricted habitats along the Yellow River Basin, tundra swans are associated with a habitat that is widely distributed along the Yangtze River Basin, indicating the risk of further expansion of the epidemic. In addition to the populations of overwintering waterfowl, there is a developed poultry industry in the Yangtze River Basin. Thus, bird host species are brought together in a high-density setting that can facilitate viral persistence, cross-species infection, and genetic reassortment, indicating the high pandemic potential of HPAI H5 viruses. Therefore, extensive surveillance systems are required to quickly detect HPAI H5 invasions and manage the outbreak in a timely manner.

Whooper swans are also responsible for the transmission of the HPAI H5N8 virus from the Yellow River Basin to Korea; however, there is no evidence of the direct migration of whooper swan from the Yellow River Basin to Korea. The absence of data suggests that it is necessary to strengthen the studies involving satellite tracking of migratory birds.

Although dead whooper swans have been occasionally found in their stopover sites (Inner Mongolia and Shaanxi), corpses have been continuously identified from November to December 2020 in wintering grounds (Henan). This indicates that intensified surveillance in the wintering grounds of whooper swan was necessary after the detection of HPAI H5 virus at their stopover sites. Furthermore, on 26 November 2020, after we detected the HPAI H5 viruses in apparently healthy whooper swan in the Sanwan wetland, Shanxi, dead whooper swans were reported. Early laboratory trials showed a decreased survival time (average of 4 days) following infection with the HPAI H5N1 virus of Asian lineage [13]. The longer duration of a live whooper swan infection would allow for ample time to travel and come into contact with other waterfowl. Thus, it was likely a factor that contributed to the spread of HPAI H5N8 viruses in China.

As early as 2016, the avian-origin HPAI H5N8 virus has been detected in dead grey seals (*Halichoerus grypus*) in Poland [54]. On December 2020, Russian poultry farm workers who participated in a response operation to a poultry H5N8 outbreak tested positive for HPAI H5N8 virus. These are the first reported detections of avian influenza A(H5N8) in humans. The E627 K and D701N substitutions in the PB2 protein have traditionally been thought to be critical for the mammalian adaptation of AIVs; however, no substitutions were observed in the Russia human isolate (A/Astrakhan/3212/2020[H5N8]) or the HPAI H5 isolates. Multiple mutations (S133A, S154N, and T156A) in the HA protein of Russia human strain and our H5 isolates were identified. Moreover, T188I and V210I mutations were observed in partial isolates, suggesting that these viruses may increase the affinity of the virus to bind to α2,6 “human-type” receptors. Taken together, these findings demonstrate that H5N8 viruses pose a considerable threat to public health, despite the limited number of human cases.

## Supplementary Material

Supplemental Material
