# Supplementary material for: Emergence, prevalence, and evolution of H5N8 avian influenza viruses in central China, 2020
Source: Emerg Microbes Infect. 2021 Dec 22;11(1):73–82. doi: 10.1080/22221751.2021.2011622 (PMC8725850; doi:10.1080/22221751.2021.2011622)
Supplement: Supplemental Material [file TEMI_A_2011622_SM3732.zip › Supplmentary files/Appendix Figure 1.docx]

**Appendix Figure 1**. Maximum-likelihood phylogenetic trees. Our isolates are marked with blue circle and the Russian human strain, A/Astrakhan/3212/2020(H5N8), is denoted by a red circle. The branches in blue, red, purple, green, and yellow represent sub-clade 2.3.4.4b1, sub-clade 2.3.4.4b1, Eurasian highly pathogenic avian influenza H5, African highly pathogenic avian influenza H5, and low pathogenic avian influenza viruses, respectively. A UFBoot support values of > 95 is shown.
