# Supplementary material for: Emergence, prevalence, and evolution of H5N8 avian influenza viruses in central China, 2020
Source: Emerg Microbes Infect. 2021 Dec 22;11(1):73–82. doi: 10.1080/22221751.2021.2011622 (PMC8725850; doi:10.1080/22221751.2021.2011622)
Supplement: Supplemental Material [file TEMI_A_2011622_SM3732.zip › Supplmentary files/Appendix-table_clean copy.docx]

**Appendix**

**Appendix Table 1**. Surveillance of wild birds in central China, 2020.

| **Province** | **Sampling site** | **Collection date** | **Coordinates** | **Sample** | | | **Influenza isolate** | | |
| --- | --- | --- | --- | --- | --- | --- | --- | --- | --- |
|  |  |  |  | **Number** | **Types** | **Host** | **Number** | **AIV positive rates (%)** | **Subtype (number)** |
| Inner Mongolia | Wuliangsuhai Lake, Bayannur city | 2020-Oct-17 | 40.95°N, 108.93°E | 2 | Organs | Whooper swan & mute swan | 2 | 100 | H5N8 (2) |
|  |  |  |  | 187 | Fecal dropping | Anatidae | 0 | 0 | 0 |
|  | **Subtotal** | | | **189** |  |  | **2** | **1.01** |  |
| Shaanxi | Hekou Reservoir, Yulin city | 2020-Nov-09 | 38.58°N, 109.28°E | 49 | Fecal dropping | Whooper swan | 1 | 2.04 | H5N8 (1) |
|  | Hongjian Nur, Yulin city | 2020-Nov-10 | 39.13°N, 109.90°E | 2 | Organs | Whooper swan & common teal | 2 | 100 | H5N8 (2) |
|  |  |  |  | 57 | Fecal dropping | Whooper swan | 1 | 1.75 | H5N8 (1) |
|  | **Subtotal** | | | **108** |  |  | **4** | **3.70** |  |
| Henan | Sanmenxia Reservoir Area, Sanmenxia city | 2020-Nov-02 | 34.78°N, 111.14°E | 1 | Oropharyngeal & cloacal swab | Whooper swan | 1 | 100 | H5N8 (1) |
|  |  | 2020-Nov-06 |  | 1 | Organ | Whooper swan | 1 | 100 | H5N8 (1) |
|  |  | 2020-Nov-09 |  | 2 | Organ | Whooper swan | 1 | 50 | H5N8 (1) |
|  |  | 2020-Nov-10 |  | 2 | Organ | Whooper swan | 2 | 100 | H5N8 (2) |
|  |  | 2020-Nov-10 |  | 132 | Fecal dropping | Whooper swan | 7 | 5.30 | H5N8 (7) |
|  |  | 2020-Nov-11 |  | 1 | Organ | Eurasian eagle-owl | 1 | 100 | H5N8 (1) |
|  |  | 2020-Nov-11 |  | 37 | Fecal dropping | Whooper swan | 0 | 0 | 0 |
|  | **Subtotal** | | | **176** |  |  | **13** | **7.38** |  |
| Hubei | Liangzi Lake, Ezhou city | 2020-Nov-02 | 30.17°N, 114.58°E | 102 | Oropharyngeal & cloacal swab | Poultry | 0 | 0 | 0 |
|  |  | 2020-Nov-13 |  | 32 | Fecal dropping | Anatidae | 0 | 0 | 0 |
|  |  | 2020-Nov-17 |  | 28 | Fecal dropping | Anatidae | 0 | 0 | 0 |
|  |  | 2020-Nov-20 |  | 31 | Fecal dropping | Anatidae | 0 | 0 | 0 |
|  | Longgan Lake, Huanggang city | 2020-Nov-04 | 29.91°N, 116.03°E | 1 | Organ | Tundra swan | 1 | 100 | H5N8 (1) |
|  |  | 2020-Nov-09 |  | 1 | Organ | Tundra swan | 1 | 100 | H5N8 (1) |
|  |  | 2020-Nov-11 |  | 1 | Organ | Tundra swan | 1 | 100 | H5N8 (1) |
|  |  | 2020-Nov-16 |  | 4 | Organ | Tundra swan | 4 | 100 | H5N8 (4) |
|  |  | 2020-Nov-16 |  | 1 | Oropharyngeal & cloacal swab | Tundra swan | 0 | 0 | 0 |
|  |  | 2020-Nov-16 |  | 1 | Organ | Whiskered tern | 1 | 100 | H5N8 (1) |
|  |  | 2020-Nov-16 |  | 1 | Oropharyngeal & cloacal swab | Bean goose | 1 | 100 | H5N8 (1) |
|  | **Subtotal** | | | **203** |  |  | **9** | **4.43** |  |
| **Total** | | | | **1075** |  |  | **42** | **3.91** |  |

**Appendix Table 2**. Isolation of avian influenza viruses from wild birds in central China, 2020.

| **Sampling date** | **Sampling site** | **Coordinates** | **Host** | | **Isolate** | **Isolate ID** |
| --- | --- | --- | --- | --- | --- | --- |
| 2020-Oct-17 | Wuliangsuhai Lake, Inner Mongolia | 40.95°N, 108.93°E | Whooper swan | *Cygnus cygnus* | A/whooper swan/Inner Mongolia/W1-1/2020(H5N8) | EPI_ISL_625671 |
| 2020-Oct-17 | Wuliangsuhai Lake, Inner Mongolia | 40.95°N, 108.93°E | Mute swan | *Cygnus olor* | A/mute swan/Inner Mongolia/W2-1/2020(H5N8) | EPI_ISL_625672 |
| 2020-Nov-09 | Hekou Reservoir, Shaanxi | 38.58°N, 109.28°E | Whooper swan | *Cygnus cygnus* | A/whooper swan/Shaanxi/SXY26/2020(H5N8) | EPI_ISL_1760446 |
| 2020-Nov-10 | Hongjian Nur, Shaanxi | 39.13°N, 109.90°E | Whooper swan | *Cygnus cygnus* | A/whooper swan/Shaanxi/SXY66/2020(H5N8) | EPI_ISL_1760447 |
| 2020-Nov-10 | Hongjian Nur, Shaanxi | 39.13°N, 109.90°E | Common teal | *Anas crecca* | A/common teal/Shaanxi/SXY1-1/2020(H5N8) | EPI_ISL_1760448 |
| 2020-Nov-10 | Hongjian Nur, Shaanxi | 39.13°N, 109.90°E | Whooper swan | *Cygnus cygnus* | A/whooper swan/Shaanxi/SXY2-1/2020(H5N8) | EPI_ISL_1760450 |
| 2020-Nov-10 | Shengtian Lake, Shanxi | 34.73°N, 110.89°E | Whooper swan | *Cygnus cygnus* | A/whooper swan/Shanxi/SX16/2020(H5N8) | EPI_ISL_1760451 |
| 2020-Nov-10 | Shengtian Lake, Shanxi | 34.73°N, 110.89°E | Whooper swan | *Cygnus cygnus* | A/whooper swan/Shanxi/SX31/2020(H5N8) | EPI_ISL_1760452 |
| 2020-Nov-10 | Shengtian Lake, Shanxi | 34.73°N, 110.89°E | Whooper swan | *Cygnus cygnus* | A/whooper swan/Shanxi/SX56/2020(H5N8) | EPI_ISL_1760453 |
| 2020-Nov-10 | Shengtian Lake, Shanxi | 34.73°N, 110.89°E | Whooper swan | *Cygnus cygnus* | A/whooper swan/Shanxi/SX106/2020(H5N8) | EPI_ISL_1760454 |
| 2020-Nov-10 | Shengtian Lake, Shanxi | 34.73°N, 110.89°E | Whooper swan | *Cygnus cygnus* | A/whooper swan/Shanxi/SX116/2020(H5N2) | EPI_ISL_1760455 |
| 2020-Nov-10 | Shengtian Lake, Shanxi | 34.73°N, 110.89°E | Whooper swan | *Cygnus cygnus* | A/whooper swan/Shanxi/SX126/2020(H9N2) | EPI_ISL_1760456 |
| 2020-Nov-10 | Sanwan wetland, Shanxi | 34.83°N, 111.15°E | Whooper swan | *Cygnus cygnus* | A/whooper swan/Shanxi/SX166/2020(H5N8) | EPI_ISL_1822592 |
| 2020-Nov-10 | Sanwan wetland, Shanxi | 34.83°N, 111.15°E | Whooper swan | *Cygnus cygnus* | A/whooper swan/Shanxi/SX206/2020(H5N8) | EPI_ISL_1822593 |
| 2020-Nov-10 | Sanwan wetland, Shanxi | 34.83°N, 111.15°E | Whooper swan | *Cygnus cygnus* | A/whooper swan/Shanxi/SX216/2020(H5N8) | EPI_ISL_1822594 |
| 2020-Nov-10 | Sanwan wetland, Shanxi | 34.83°N, 111.15°E | Whooper swan | *Cygnus cygnus* | A/whooper swan/Shanxi/SX231/2020(H5N8) | EPI_ISL_1822595 |
| 2020-Nov-10 | Sanwan wetland, Shanxi | 34.83°N, 111.15°E | Whooper swan | *Cygnus cygnus* | A/whooper swan/Shanxi/SX251/2020(H5N8) | EPI_ISL_1822596 |
| 2020-Nov-10 | Sanwan wetland, Shanxi | 34.83°N, 111.15°E | Whooper swan | *Cygnus cygnus* | A/whooper swan/Shanxi/SX276/2020(H5N8) | EPI_ISL_1822597 |
| 2020-Nov-10 | Sanwan wetland, Shanxi | 34.83°N, 111.15°E | Whooper swan | *Cygnus cygnus* | A/whooper swan/Shanxi/SX291/2020(H5N8) | EPI_ISL_1822598 |
| 2020-Nov-10 | Sanwan wetland, Shanxi | 34.83°N, 111.15°E | Whooper swan | *Cygnus cygnus* | A/whooper swan/Shanxi/SX346/2020(H5N8) | EPI_ISL_1822599 |
| 2020-Nov-02 | Sanmenxia Reservoir Area, Henan | 34.78°N, 111.14°E | Whooper swan | *Cygnus cygnus* | A/whooper swan/Henan/SMQ5/2020(H5N8) | EPI_ISL_1829205 |
| 2020-Nov-06 | Sanmenxia Reservoir Area, Henan | 34.78°N, 111.14°E | Whooper swan | *Cygnus cygnus* | A/whooper swan/Henan/SMQ6/2020(H5N8) | EPI_ISL_1834185 |
| 2020-Nov-09 | Sanmenxia Reservoir Area, Henan | 34.78°N, 111.14°E | Whooper swan | *Cygnus cygnus* | A/whooper swan/Henan/SMQ7/2020(H5N8) | EPI_ISL_1937883 |
| 2020-Nov-10 | Sanmenxia Reservoir Area, Henan | 34.78°N, 111.14°E | Whooper swan | *Cygnus cygnus* | A/whooper swan/Henan/SM1/2020(H5N8) | EPI_ISL_1937884 |
| 2020-Nov-10 | Sanmenxia Reservoir Area, Henan | 34.78°N, 111.14°E | Whooper swan | *Cygnus cygnus* | A/whooper swan/Henan/SM16/2020(H5N8) | EPI_ISL_1937889 |
| 2020-Nov-10 | Sanmenxia Reservoir Area, Henan | 34.78°N, 111.14°E | Whooper swan | *Cygnus cygnus* | A/whooper swan/Henan/SM31/2020(H5N8) | EPI_ISL_1937890 |
| 2020-Nov-10 | Sanmenxia Reservoir Area, Henan | 34.78°N, 111.14°E | Whooper swan | *Cygnus cygnus* | A/whooper swan/Henan/SM61/2020(H5N8) | EPI_ISL_1937891 |
| 2020-Nov-10 | Sanmenxia Reservoir Area, Henan | 34.78°N, 111.14°E | Whooper swan | *Cygnus cygnus* | A/whooper swan/Henan/SM76/2020(H5N8) | EPI_ISL_1937892 |
| 2020-Nov-10 | Sanmenxia Reservoir Area, Henan | 34.78°N, 111.14°E | Whooper swan | *Cygnus cygnus* | A/whooper swan/Henan/SM86/2020(H5N8) | EPI_ISL_1938307 |
| 2020-Nov-10 | Sanmenxia Reservoir Area, Henan | 34.78°N, 111.14°E | Whooper swan | *Cygnus cygnus* | A/whooper swan/Henan/SM111/2020(H5N8) | EPI_ISL_1938309 |
| 2020-Nov-10 | Sanmenxia Reservoir Area, Henan | 34.78°N, 111.14°E | Whooper swan | *Cygnus cygnus* | A/whooper swan/Henan/SMQ9/2020(H5N8) | EPI_ISL_1938478 |
| 2020-Nov-10 | Sanmenxia Reservoir Area, Henan | 34.78°N, 111.14°E | Whooper swan | *Cygnus cygnus* | A/whooper swan/Henan/SMQ10/2020(H5N8) | EPI_ISL_1939080 |
| 2020-Nov-11 | Sanmenxia Reservoir Area, Henan | 34.78°N, 111.14°E | Eurasian eagle-owl | *Bubo bubo* | A/eurasian eagle-owl/Henan/SMQ11/2020(H5N8) | EPI_ISL_1939617 |
| 2020-Nov-04 | Longgan Lake, Hubei | 29.91°N, 116.03°E | Tundra swan | *Cygnus columbianus* | A/tundra swan/Hubei/BQ2/2020(H5N8) | EPI_ISL_2685842 |
| 2020-Nov-09 | Longgan Lake, Hubei | 29.91°N, 116.03°E | Tundra swan | *Cygnus columbianus* | A/tundra swan/Hubei/BQ3/2020(H5N8) | EPI_ISL_2685846 |
| 2020-Nov-11 | Longgan Lake, Hubei | 29.91°N, 116.03°E | Tundra swan | *Cygnus columbianus* | A/tundra swan/Hubei/BQ4/2020(H5N8) | EPI_ISL_2685847 |
| 2020-Nov-16 | Longgan Lake, Hubei | 29.91°N, 116.03°E | Tundra swan | *Cygnus columbianus* | A/tundra swan/Hubei/BQ6/2020(H5N8) | EPI_ISL_2685848 |
| 2020-Nov-16 | Longgan Lake, Hubei | 29.91°N, 116.03°E | Tundra swan | *Cygnus columbianus* | A/tundra swan/Hubei/BQ7/2020(H5N8) | EPI_ISL_2685900 |
| 2020-Nov-16 | Longgan Lake, Hubei | 29.91°N, 116.03°E | Tundra swan | *Cygnus columbianus* | A/tundra swan/Hubei/BQ8/2020(H5N8) | EPI_ISL_2685947 |
| 2020-Nov-16 | Longgan Lake, Hubei | 29.91°N, 116.03°E | Tundra swan | *Cygnus columbianus* | A/tundra swan/Hubei/BQ9/2020(H5N8) | EPI_ISL_2713170 |
| 2020-Nov-16 | Longgan Lake, Hubei | 29.91°N, 116.03°E | Whiskered tern | *Chlidonias hybrida* | A/whiskered tern/Hubei/BQ10/2020(H5N8) | EPI_ISL_2713314 |
| 2020-Nov-16 | Longgan Lake, Hubei | 29.91°N, 116.03°E | Bean goose | *Anser fabalis* | A/bean goose/Hubei/BQ11/2020(H5N8) | EPI_ISL_2713372 |

**Appendix Table 3.** Geographic categories and host-type of sequence dataset.

| Trait | Data set | Number of sequences |
| --- | --- | --- |
| Geographic categories | Central Asia (Iraq and Kazakhstan) | 7 |
|  | Europe | 91 |
|  | Korea | 22 |
|  | Russia | 79 |
|  | Yangzi River Basin (Hubei, China) | 9 |
|  | Yellow River Basin (Inner Mongolia, Shaanxi, Shanxi, and Henan, China) | 31 |
| Host-type | Aquatic poultry | 43 |
|  | Terrestrial poultry | 81 |
|  | Mute swan | 17 |
|  | Tundra swan | 7 |
|  | Whooper swan | 28 |
|  | Wild ducks and geese (except mute swan, tundra swan, and whooper swan) | 42 |
|  | Others | 21 |

**Appendix Table 4**. Statistically supported diffusion rates of epidemiological links between geographic locations.

| From | To | Bayes factor | Posterior probability |
| --- | --- | --- | --- |
| Europe | Russia | 6.28 | 0.60 |
| Russia | Yellow River Basin | 7.67 | 0.64 |
| Russia | Central Asia | >1000 | 1 |
| Russia | Europe | >1000 | 1 |
| Yellow River Basin | Korea | 42.46 | 0.91 |
| Yellow River Basin | Yangtze River Basin | 26.05 | 0.86 |

**Appendix Table 5**. Statistically supported diffusion rates of epidemiological links between host-types.

| From | To | Bayes factor | Posterior probability |
| --- | --- | --- | --- |
| Aquatic poultry | Terrestrial poultry | >1000 | 1 |
| Aquatic poultry | Wild ducks and geese | 27.36 | 0.84 |
| Aquatic poultry | Whooper swan | 5.41 | 0.51 |
| Terrestrial poultry | Aquatic poultry | 17.80 | 0.77 |
| Terrestrial poultry | Wild ducks and geese | 7.58 | 0.59 |
| Terrestrial poultry | Others | 248.63 | 0.98 |
| Wild ducks and geese | Terrestrial poultry | 197.06 | 0.97 |
| Wild ducks and geese | Mute swan | 36.08 | 0.87 |
| Wild ducks and geese | Others | 16.35 | 0.76 |
| Tundra swan | Wild ducks and geese | 14.05 | 0.73 |
| Tundra swan | Others | 17.58 | 0.77 |
| Whooper swan | Wild ducks and geese | 76.07 | 0.94 |
| Whooper swan | Tundra swan | 12.26 | 0.70 |
| Whooper swan | Others | 13.28 | 0.72 |

**Appendix Table 6**. Key molecular markers of our isolates and the Russian human strain, A/Astrakhan/3212/2020(H5N8).

| **Isolate** | **HA: Mutation/location H5 numbering relative to A/Vietnam/1203/2004(H5N1)** | | | | | | | | | | | | | | | | | | | | | | |
| --- | --- | --- | --- | --- | --- | --- | --- | --- | --- | --- | --- | --- | --- | --- | --- | --- | --- | --- | --- | --- | --- | --- | --- |
|  | **94 D/N** | **121 S/N** | **133 S/A** | **134 A/V** | **139 G/R** | **154 S/N** | **155 S/N** | **156 T/A** | **182 N/K, D** | **183 D/G** | **186 E/G** | **188 T/I** | **189 K/R, T** | **192 Q/R, H** | **193 N/K** | **210 V/I** | **218 K/Q** | **221 G/D** | **222 Q/L** | **223 S/N** | **224 G/S** | **235 P/S** | **251 E/K** |
| A/Astrakhan/3212/2020(H5N8) | S | S | A | A | G | N | D | A | N | N | E | T | N | K | N | V | Q | G | Q | R | G | P | E |
| A/whooper swan/Inner Mongolia/W1-1/2020(H5N8) | S | S | A | A | G | N | D | A | N | N | E | I | N | K | N | V | Q | G | Q | R | G | P | E |
| A/mute swan/Inner Mongolia/W2-1/2020(H5N8) | S | S | A | A | G | N | D | A | N | N | E | I | N | K | N | V | Q | G | Q | R | G | P | E |
| A/whooper swan/Shaanxi/SXY26/2020(H5N8) | S | S | A | A | G | N | D | A | N | N | E | I | N | K | N | V | Q | G | Q | R | G | P | E |
| A/whooper swan/Shaanxi/SXY66/2020(H5N8) | S | S | A | A | G | N | D | A | N | N | E | I | N | K | N | V | Q | G | Q | R | G | P | E |
| A/common teal/Shaanxi/SXY1-1/2020(H5N8) | S | S | A | A | G | N | D | A | N | N | E | I | N | K | N | I | Q | G | Q | R | G | P | E |
| A/whooper swan/Shaanxi/SXY2-1/2020(H5N8) | S | S | A | A | G | N | D | A | N | N | E | I | N | K | N | I | Q | G | Q | R | G | P | E |
| A/whooper swan/Shanxi/SX16/2020(H5N8) | S | S | A | A | G | N | D | A | N | N | E | T | N | K | N | V | Q | G | Q | R | G | P | E |
| A/whooper swan/Shanxi/SX31/2020(H5N8) | S | S | A | A | G | N | D | A | N | N | E | I | N | K | N | V | Q | G | Q | R | G | P | E |
| A/whooper swan/Shanxi/SX56/2020(H5N8) | S | S | A | A | G | N | D | A | N | N | E | T | N | K | N | V | Q | G | Q | R | G | P | E |
| A/whooper swan/Shanxi/SX106/2020(H5N8) | S | S | A | A | G | N | D | A | N | N | E | I | N | K | N | V | Q | G | Q | R | G | P | E |
| A/whooper swan/Shanxi/SX116/2020(H5N2) | S | S | A | A | G | N | D | A | N | N | E | T | N | K | N | V | Q | G | Q | R | G | P | E |
| A/whooper swan/Shanxi/SX126/2020(H9N2) | N | I | K | A | - | S | N | S | P | T | E | T | N | K | K | N | L | G | Q | Q | G | P | W |
| A/whooper swan/Shanxi/SX166/2020(H5N8) | S | S | A | A | G | N | D | A | N | N | E | I | N | K | N | V | Q | G | Q | R | G | P | E |
| A/whooper swan/Shanxi/SX206/2020(H5N8) | S | S | A | A | G | N | D | A | N | N | E | I | N | K | N | V | Q | G | Q | R | G | P | E |
| A/whooper swan/Shanxi/SX216/2020(H5N8) | S | S | A | A | G | N | D | A | N | N | E | I | N | K | N | V | Q | G | Q | R | G | P | E |
| A/whooper swan/Shanxi/SX231/2020(H5N8) | S | S | A | A | G | N | D | A | N | N | E | I | N | K | N | V | Q | G | Q | R | G | P | E |
| A/whooper swan/Shanxi/SX251/2020(H5N8) | S | S | A | A | G | N | D | A | N | N | E | I | N | K | N | V | Q | G | Q | R | G | P | E |
| A/whooper swan/Shanxi/SX276/2020(H5N8) | S | S | A | A | G | N | D | A | N | N | E | I | N | K | N | V | Q | G | Q | R | G | P | E |
| A/whooper swan/Shanxi/SX291/2020(H5N8) | S | S | A | A | G | N | D | A | N | N | E | I | N | K | N | V | Q | G | Q | R | G | P | E |
| A/whooper swan/Shanxi/SX346/2020(H5N8) | S | S | A | A | G | N | D | A | N | N | E | I | N | K | N | V | Q | G | Q | R | G | P | E |
| A/whooper swan/Henan/SM1/2020(H5N8) | S | S | A | A | G | N | D | A | N | N | E | I | N | K | N | V | Q | G | Q | R | G | P | E |
| A/whooper swan/Henan/SM16/2020(H5N8) | S | S | A | A | G | N | D | A | N | N | E | I | N | K | N | I | Q | G | Q | R | G | P | E |
| A/whooper swan/Henan/SM31/2020(H5N8) | S | S | A | A | G | N | D | A | N | N | E | I | N | K | N | I | Q | G | Q | R | G | P | E |
| A/whooper swan/Henan/SM61/2020(H5N8) | S | S | A | A | G | N | D | A | N | N | E | I | N | K | N | V | Q | G | Q | R | G | P | E |
| A/whooper swan/Henan/SM76/2020(H5N8) | S | S | A | A | G | N | D | A | N | N | E | I | N | K | N | V | Q | G | Q | R | G | P | E |
| A/whooper swan/Henan/SM86/2020(H5N8) | S | S | A | A | G | N | D | A | N | N | E | I | N | K | N | V | Q | G | Q | R | G | P | E |
| A/whooper swan/Henan/SM111/2020(H5N8) | S | S | A | A | G | N | D | A | N | N | E | I | N | K | N | V | Q | G | Q | R | G | P | E |
| A/whooper swan/Henan/SMQ5/2020(H5N8) | S | S | A | A | G | N | D | A | N | N | E | I | N | K | N | V | Q | G | Q | R | G | P | E |
| A/whooper swan/Henan/SMQ6/2020(H5N8) | S | S | A | A | G | N | D | A | N | N | E | I | N | K | N | V | Q | G | Q | R | G | P | E |
| A/whooper swan/Henan/SMQ7/2020(H5N8) | S | S | A | A | G | N | D | A | N | N | E | I | N | K | N | V | Q | G | Q | R | G | P | E |
| A/whooper swan/Henan/SMQ9/2020(H5N8) | S | S | A | A | G | N | D | A | N | N | E | I | N | K | N | I | Q | G | Q | R | G | P | E |
| A/whooper swan/Henan/SMQ10/2020(H5N8) | S | S | A | A | G | N | D | A | N | N | E | I | N | K | N | I | Q | G | Q | R | G | P | E |
| A/eurasian eagle-owl/Henan/SMQ11/2020(H5N8) | S | S | A | A | G | N | D | A | N | N | E | I | N | K | N | I | Q | G | Q | R | G | P | E |
| A/tundra swan/Hubei/BQ2/2020(H5N8) | S | S | A | A | G | N | D | A | N | N | E | I | N | K | N | V | Q | G | Q | R | G | P | E |
| A/tundra swan/Hubei/BQ3/2020(H5N8) | S | S | A | A | G | N | D | A | N | N | E | T | N | K | N | V | Q | G | Q | R | G | P | E |
| A/tundra swan/Hubei/BQ4/2020(H5N8) | S | S | A | A | G | N | D | A | N | N | E | I | N | K | N | V | Q | G | Q | R | G | P | E |
| A/tundra swan/Hubei/BQ6/2020(H5N8) | S | S | A | A | G | N | D | A | N | N | E | I | N | K | N | V | Q | G | Q | R | G | P | E |
| A/tundra swan/Hubei/BQ7/2020(H5N8) | S | S | A | A | G | N | D | A | N | N | E | I | N | K | N | V | Q | G | Q | R | G | P | E |
| A/tundra swan/Hubei/BQ8/2020(H5N8) | S | S | A | A | G | N | D | A | N | N | E | I | N | K | N | V | Q | G | Q | R | G | P | E |
| A/tundra swan/Hubei/BQ9/2020(H5N8) | S | S | A | A | G | N | D | A | N | N | E | I | N | K | N | V | Q | G | Q | R | G | P | E |
| A/whiskered tern/Hubei/BQ10/2020(H5N8) | S | S | A | A | G | N | D | A | N | N | E | I | N | K | N | V | Q | G | Q | R | G | P | E |
| A/bean goose/Hubei/BQ11/2020(H5N8) | S | S | A | A | G | N | D | A | N | N | E | I | N | K | N | V | Q | G | Q | R | G | P | E |

| **Isolate** | **NA: Mutation/location N2 numbering relative to A/Aichi/2/1968(H3N2)** | | | | | | | | | | | | | | **PB2** | | | | | | | | | |
| --- | --- | --- | --- | --- | --- | --- | --- | --- | --- | --- | --- | --- | --- | --- | --- | --- | --- | --- | --- | --- | --- | --- | --- | --- |
|  | **116 V/A** | **117 I/T** | **119 E/D, G, A** | **136 Q/L, K, R** | **149 V/A** | **156 R/K** | **198 D/G** | **222 I/M** | **246 S/N** | **274 H/Y** | **277 E/Q** | **294 N/S** | **295 N/S** | **432 K/T** | **9 D/N** | **25 V/A** | **158 E/G** | **192 E/K** | **199 A/S** | **256 D/G** | **526 K/R** | **591 Q/K** | **627 E/K, V** | **701 D/N, V** |
| A/Astrakhan/3212/2020(H5N8) | V | I | E | Q | V | R | D | I | S | H | E | N | W | E | D | V | E | E | A | D | K | Q | E | D |
| A/whooper swan/Inner Mongolia/W1-1/2020(H5N8) | V | I | E | Q | V | R | D | I | S | H | E | N | W | E | D | V | E | E | A | D | K | Q | E | D |
| A/mute swan/Inner Mongolia/W2-1/2020(H5N8) | V | I | E | Q | V | R | D | I | S | H | E | N | W | E | D | V | E | E | A | D | K | Q | E | D |
| A/whooper swan/Shaanxi/SXY26/2020(H5N8) | V | I | E | Q | V | R | D | I | S | H | E | N | W | E | D | V | E | E | A | D | K | Q | E | D |
| A/whooper swan/Shaanxi/SXY66/2020(H5N8) | V | I | E | Q | V | R | D | I | S | H | E | N | W | E | D | V | E | E | A | D | K | Q | E | D |
| A/common teal/Shaanxi/SXY1-1/2020(H5N8) | V | I | E | Q | V | R | D | I | S | H | E | N | W | E | D | V | E | E | A | D | K | Q | E | D |
| A/whooper swan/Shaanxi/SXY2-1/2020(H5N8) | V | I | E | Q | V | R | D | I | S | H | E | N | W | E | D | V | E | E | A | D | K | Q | E | D |
| A/whooper swan/Shanxi/SX16/2020(H5N8) | V | I | E | Q | V | R | D | I | S | H | E | N | W | E | D | V | E | E | A | D | K | Q | E | D |
| A/whooper swan/Shanxi/SX31/2020(H5N8) | V | I | E | Q | V | R | D | I | S | H | E | N | W | E | D | V | E | E | A | D | K | Q | E | D |
| A/whooper swan/Shanxi/SX56/2020(H5N8) | V | I | E | Q | V | R | D | I | S | H | E | N | W | E | D | V | E | E | A | D | K | Q | E | D |
| A/whooper swan/Shanxi/SX106/2020(H5N8) | V | I | E | Q | V | R | D | I | S | H | E | N | W | E | D | V | E | E | A | D | K | Q | E | D |
| A/whooper swan/Shanxi/SX116/2020(H5N2) | V | T | E | Q | I | R | D | I | A | H | E | N | W | Q | D | V | E | E | A | D | K | Q | E | D |
| A/whooper swan/Shanxi/SX126/2020(H9N2) | V | T | E | Q | I | R | D | I | A | H | E | N | W | Q | D | V | E | E | A | D | K | Q | E | D |
| A/whooper swan/Shanxi/SX166/2020(H5N8) | V | I | E | Q | V | R | D | I | S | H | E | N | W | E | D | V | E | E | A | D | K | Q | E | D |
| A/whooper swan/Shanxi/SX206/2020(H5N8) | V | I | E | Q | V | R | D | I | S | H | E | N | W | E | D | V | E | E | A | D | K | Q | E | D |
| A/whooper swan/Shanxi/SX216/2020(H5N8) | V | I | E | Q | V | R | D | I | S | H | E | N | W | E | D | V | E | E | A | D | K | Q | E | D |
| A/whooper swan/Shanxi/SX231/2020(H5N8) | V | I | E | Q | V | R | D | I | S | H | E | N | W | E | D | V | E | E | A | D | K | Q | E | D |
| A/whooper swan/Shanxi/SX251/2020(H5N8) | V | I | E | Q | V | R | D | I | S | H | E | N | W | E | D | V | E | E | A | D | K | Q | E | D |
| A/whooper swan/Shanxi/SX276/2020(H5N8) | V | I | E | Q | V | R | D | I | S | H | E | N | W | E | D | V | E | E | A | D | K | Q | E | D |
| A/whooper swan/Shanxi/SX291/2020(H5N8) | V | I | E | Q | V | R | D | I | S | H | E | N | W | E | D | V | E | E | A | D | K | Q | E | D |
| A/whooper swan/Shanxi/SX346/2020(H5N8) | V | I | E | Q | V | R | D | I | S | H | E | N | W | E | D | V | E | E | A | D | K | Q | E | D |
| A/whooper swan/Henan/SM1/2020(H5N8) | V | I | E | Q | V | R | D | I | S | H | E | N | W | E | D | V | E | E | A | D | K | Q | E | D |
| A/whooper swan/Henan/SM16/2020(H5N8) | V | I | E | Q | V | R | D | I | S | H | E | N | W | E | D | V | E | E | A | D | K | Q | E | D |
| A/whooper swan/Henan/SM31/2020(H5N8) | V | I | E | Q | V | R | D | I | S | H | E | N | W | E | D | V | E | E | A | D | K | Q | E | D |
| A/whooper swan/Henan/SM61/2020(H5N8) | V | I | E | Q | V | R | D | I | S | H | E | N | W | E | D | V | E | E | A | D | K | Q | E | D |
| A/whooper swan/Henan/SM76/2020(H5N8) | V | I | E | Q | V | R | D | I | S | H | E | N | W | E | D | V | E | E | A | D | K | Q | E | D |
| A/whooper swan/Henan/SM86/2020(H5N8) | V | I | E | Q | V | R | D | I | S | H | E | N | W | E | D | V | E | E | A | D | K | Q | E | D |
| A/whooper swan/Henan/SM111/2020(H5N8) | V | I | E | Q | V | R | D | I | S | H | E | N | W | E | D | V | E | E | A | D | K | Q | E | D |
| A/whooper swan/Henan/SMQ5/2020(H5N8) | V | I | E | Q | V | R | D | I | S | H | E | N | W | E | D | V | E | E | A | D | K | Q | E | D |
| A/whooper swan/Henan/SMQ6/2020(H5N8) | V | I | E | Q | V | R | D | I | S | H | E | N | W | E | D | V | E | E | A | D | K | Q | E | D |
| A/whooper swan/Henan/SMQ7/2020(H5N8) | V | I | E | Q | V | R | D | I | S | H | E | N | W | E | D | V | E | E | A | D | K | Q | E | D |
| A/whooper swan/Henan/SMQ9/2020(H5N8) | V | I | E | Q | V | R | D | I | S | H | E | N | W | E | D | V | E | E | A | D | K | Q | E | D |
| A/whooper swan/Henan/SMQ10/2020(H5N8) | V | I | E | Q | V | R | D | I | S | H | E | N | W | E | D | V | E | E | A | D | K | Q | E | D |
| A/eurasian eagle-owl/Henan/SMQ11/2020(H5N8) | V | I | E | Q | V | R | D | I | S | H | E | N | W | E | D | V | E | E | A | D | K | Q | E | D |
| A/tundra swan/Hubei/BQ2/2020(H5N8) | V | I | E | Q | V | R | D | I | S | H | E | N | W | E | D | V | E | E | A | D | K | Q | E | D |
| A/tundra swan/Hubei/BQ3/2020(H5N8) | V | I | E | Q | V | R | D | I | P | H | E | N | W | E | D | V | E | E | A | D | K | Q | E | D |
| A/tundra swan/Hubei/BQ4/2020(H5N8) | V | I | E | Q | V | R | D | I | P | H | E | N | W | E | D | V | E | E | A | D | K | Q | E | D |
| A/tundra swan/Hubei/BQ6/2020(H5N8) | V | I | E | Q | V | R | D | I | S | H | E | N | W | E | D | V | E | E | A | D | K | Q | E | D |
| A/tundra swan/Hubei/BQ7/2020(H5N8) | V | I | E | Q | V | R | D | I | S | H | E | N | W | E | D | V | E | E | A | D | K | Q | E | D |
| A/tundra swan/Hubei/BQ8/2020(H5N8) | V | I | E | Q | V | R | D | I | S | H | E | N | W | E | D | V | E | E | A | D | K | Q | E | D |
| A/tundra swan/Hubei/BQ9/2020(H5N8) | V | I | E | Q | V | R | D | I | S | H | E | N | W | E | D | V | E | E | A | D | K | Q | E | D |
| A/whiskered tern/Hubei/BQ10/2020(H5N8) | V | I | E | Q | V | R | D | I | S | H | E | N | W | E | D | V | E | E | A | D | K | Q | E | D |
| A/bean goose/Hubei/BQ11/2020(H5N8) | V | I | E | Q | V | R | D | I | S | H | E | N | W | E | D | V | E | E | A | D | K | Q | E | D |

| **Isolate** | **PB1** | | | | | **PB1-F2** | **PA** | | | | | | **NP** | | | | | | | **M1** | | | |
| --- | --- | --- | --- | --- | --- | --- | --- | --- | --- | --- | --- | --- | --- | --- | --- | --- | --- | --- | --- | --- | --- | --- | --- |
|  | **3 D/V** | **99 H/Y** | **105 N/S** | **622 D/G** | **677 T/M** | **66 N/S** | **97 T/I** | **142 K/N, E** | **159 K/R** | **383 N/D** | **421 S/I** | **443 R/K** | **105 M/V** | **109 I/T** | **184 A/K** | **227 K/R** | **229 K/R** | **319 N/K** | **470 K/R** | **30 N/D** | **43 I/M** | **139 T/A** | **215 T/A** |
| A/Astrakhan/3212/2020(H5N8) | V | H | N | G | T | - | T | K | A | D | S | R | V | I | K | K | K | N | K | D | M | T | A |
| A/whooper swan/Inner Mongolia/W1-1/2020(H5N8) | V | H | N | G | T | N | T | K | A | D | S | R | V | I | K | K | K | N | K | D | M | T | A |
| A/mute swan/Inner Mongolia/W2-1/2020(H5N8) | V | H | N | G | T | N | T | K | A | D | S | R | V | I | K | K | K | N | K | D | M | T | A |
| A/whooper swan/Shaanxi/SXY26/2020(H5N8) | V | H | N | G | T | N | T | K | A | D | S | R | V | I | K | K | K | N | K | D | M | T | A |
| A/whooper swan/Shaanxi/SXY66/2020(H5N8) | V | H | N | G | T | N | T | K | A | D | S | R | V | I | K | K | K | N | K | D | M | T | A |
| A/common teal/Shaanxi/SXY1-1/2020(H5N8) | V | H | N | G | T | N | T | K | A | D | S | R | V | I | K | K | K | N | K | D | M | T | A |
| A/whooper swan/Shaanxi/SXY2-1/2020(H5N8) | V | H | N | G | T | N | T | K | A | D | S | R | V | I | K | K | K | N | K | D | M | T | A |
| A/whooper swan/Shanxi/SX16/2020(H5N8) | V | H | N | G | T | N | T | K | A | D | S | R | V | I | K | K | K | N | K | D | M | T | A |
| A/whooper swan/Shanxi/SX31/2020(H5N8) | V | H | N | G | T | N | T | K | A | D | S | R | V | I | K | K | K | N | K | D | M | T | A |
| A/whooper swan/Shanxi/SX56/2020(H5N8) | V | H | N | G | T | N | T | K | A | D | S | R | V | I | K | K | K | N | K | D | M | T | A |
| A/whooper swan/Shanxi/SX106/2020(H5N8) | V | H | N | G | T | N | T | K | A | D | S | R | V | I | K | K | K | N | K | D | M | T | A |
| A/whooper swan/Shanxi/SX116/2020(H5N2) | V | H | N | G | T | N | T | K | A | D | S | R | V | I | K | K | K | N | K | D | M | T | A |
| A/whooper swan/Shanxi/SX126/2020(H9N2) | V | H | N | G | T | N | T | K | A | D | S | R | V | I | K | K | K | N | K | D | M | T | A |
| A/whooper swan/Shanxi/SX166/2020(H5N8) | V | H | N | G | T | N | T | K | A | D | S | R | V | I | K | K | K | N | K | D | M | T | A |
| A/whooper swan/Shanxi/SX206/2020(H5N8) | V | H | N | G | T | N | T | K | A | D | S | R | V | I | K | K | K | N | K | D | M | T | A |
| A/whooper swan/Shanxi/SX216/2020(H5N8) | V | H | N | G | T | N | T | K | A | D | S | R | V | I | K | K | K | N | K | D | M | T | A |
| A/whooper swan/Shanxi/SX231/2020(H5N8) | V | H | N | G | T | N | T | K | A | D | S | R | V | I | K | K | K | N | K | D | M | T | A |
| A/whooper swan/Shanxi/SX251/2020(H5N8) | V | H | N | G | T | N | T | K | A | D | S | R | V | I | K | K | K | N | K | D | M | T | A |
| A/whooper swan/Shanxi/SX276/2020(H5N8) | V | H | N | G | T | N | T | K | A | D | S | R | V | I | K | K | K | N | K | D | M | T | A |
| A/whooper swan/Shanxi/SX291/2020(H5N8) | V | H | N | G | T | N | T | K | A | D | S | R | V | I | K | K | K | N | K | D | M | T | A |
| A/whooper swan/Shanxi/SX346/2020(H5N8) | V | H | N | G | T | N | T | K | A | D | S | R | V | I | K | K | K | N | K | D | M | T | A |
| A/whooper swan/Henan/SM1/2020(H5N8) | V | H | N | G | T | N | T | K | A | D | S | R | V | I | K | K | K | N | K | D | M | T | A |
| A/whooper swan/Henan/SM16/2020(H5N8) | V | H | N | G | T | N | T | K | A | D | S | R | V | I | K | K | K | N | K | D | M | T | A |
| A/whooper swan/Henan/SM31/2020(H5N8) | V | H | N | G | T | N | T | K | A | D | S | R | V | I | K | K | K | N | K | D | M | T | A |
| A/whooper swan/Henan/SM61/2020(H5N8) | V | H | N | G | T | N | T | K | A | D | S | R | V | I | K | K | K | N | K | D | M | T | A |
| A/whooper swan/Henan/SM76/2020(H5N8) | V | H | N | G | T | N | T | K | A | D | S | R | V | I | K | K | K | N | K | D | M | T | A |
| A/whooper swan/Henan/SM86/2020(H5N8) | V | H | N | G | T | N | T | K | A | D | S | R | V | I | K | K | K | N | K | D | M | T | A |
| A/whooper swan/Henan/SM111/2020(H5N8) | V | H | N | G | T | N | T | K | A | D | S | R | V | I | K | K | K | N | K | D | M | T | A |
| A/whooper swan/Henan/SMQ5/2020(H5N8) | V | H | N | G | T | N | T | K | A | D | S | R | V | I | K | K | K | N | K | D | M | T | A |
| A/whooper swan/Henan/SMQ6/2020(H5N8) | V | H | N | G | T | N | T | K | A | D | S | R | V | I | K | K | K | N | K | D | M | T | A |
| A/whooper swan/Henan/SMQ7/2020(H5N8) | V | H | N | G | T | N | T | K | A | D | S | R | V | I | K | K | K | N | K | D | M | T | A |
| A/whooper swan/Henan/SMQ9/2020(H5N8) | V | H | N | G | T | N | T | K | A | D | S | R | V | I | K | K | K | N | K | D | M | T | A |
| A/whooper swan/Henan/SMQ10/2020(H5N8) | V | H | N | G | T | N | T | K | A | D | S | R | V | I | K | K | K | N | K | D | M | T | A |
| A/eurasian eagle-owl/Henan/SMQ11/2020(H5N8) | V | H | N | G | T | N | T | K | A | D | S | R | V | I | K | K | K | N | K | D | M | T | A |
| A/tundra swan/Hubei/BQ2/2020(H5N8) | V | H | N | G | T | N | T | K | A | D | S | R | V | I | K | K | K | N | K | D | M | T | A |
| A/tundra swan/Hubei/BQ3/2020(H5N8) | V | H | N | G | T | N | T | K | A | D | S | R | V | I | K | K | K | N | K | D | M | T | A |
| A/tundra swan/Hubei/BQ4/2020(H5N8) | V | H | N | G | T | N | T | K | A | D | S | R | V | I | K | K | K | N | K | D | M | T | A |
| A/tundra swan/Hubei/BQ6/2020(H5N8) | V | H | N | G | T | N | T | K | A | D | S | R | V | I | K | K | K | N | K | D | M | T | A |
| A/tundra swan/Hubei/BQ7/2020(H5N8) | V | H | N | G | T | N | T | K | A | D | S | R | V | I | K | K | K | N | K | D | M | T | A |
| A/tundra swan/Hubei/BQ8/2020(H5N8) | V | H | N | G | T | N | T | K | A | D | S | R | V | I | K | K | K | N | K | D | M | T | A |
| A/tundra swan/Hubei/BQ9/2020(H5N8) | V | H | N | G | T | N | T | K | A | D | S | R | V | I | K | K | K | N | K | D | M | T | A |
| A/whiskered tern/Hubei/BQ10/2020(H5N8) | V | H | N | G | T | N | T | K | A | D | S | R | V | I | K | K | K | N | K | D | M | T | A |
| A/bean goose/Hubei/BQ11/2020(H5N8) | V | H | N | G | T | N | T | K | A | D | S | R | V | I | K | K | K | N | K | D | M | T | A |

| **Isolate** | **M2** | | | | | **NS1** | | | | | | | | | | **NS2** | | |
| --- | --- | --- | --- | --- | --- | --- | --- | --- | --- | --- | --- | --- | --- | --- | --- | --- | --- | --- |
|  | **26 L/F** | **27 I, V/A, T, S** | **30 A/V, T, S** | **31 S/N, G** | **34 G/E** | **42 P/S** | **80-84 deletion** | **92 D/E** | **103 L/F** | **106 I/M** | **138 C/F** | **149 V/A** | **200 N/S** | **205 G/R** | **222-225 PDZ domain** | **16 M/I** | **47 T/A** | **51 M/I** |
| A/Astrakhan/3212/2020(H5N8) | L | V | A | S | G | S | No | D | F | M | F | A | N | G | No | M | T | M |
| A/whooper swan/Inner Mongolia/W1-1/2020(H5N8) | L | V | A | S | G | S | No | D | F | M | F | A | N | G | No | M | T | M |
| A/mute swan/Inner Mongolia/W2-1/2020(H5N8) | L | V | A | S | G | S | No | D | F | M | F | A | N | G | No | M | T | M |
| A/whooper swan/Shaanxi/SXY26/2020(H5N8) | L | V | A | S | G | S | No | D | F | M | F | A | N | G | No | M | T | M |
| A/whooper swan/Shaanxi/SXY66/2020(H5N8) | L | V | A | S | G | S | No | D | F | M | F | A | N | G | No | M | T | M |
| A/common teal/Shaanxi/SXY1-1/2020(H5N8) | L | V | A | S | G | S | No | D | F | M | F | A | N | G | No | M | T | M |
| A/whooper swan/Shaanxi/SXY2-1/2020(H5N8) | L | V | A | S | G | S | No | D | F | M | F | A | N | G | No | M | T | M |
| A/whooper swan/Shanxi/SX16/2020(H5N8) | L | V | A | S | G | S | No | D | F | M | F | A | N | G | No | M | T | M |
| A/whooper swan/Shanxi/SX31/2020(H5N8) | L | V | A | S | G | S | No | D | F | M | F | A | N | G | No | M | T | M |
| A/whooper swan/Shanxi/SX56/2020(H5N8) | L | V | A | S | G | S | No | D | F | M | F | A | N | G | No | M | T | M |
| A/whooper swan/Shanxi/SX106/2020(H5N8) | L | V | A | S | G | S | No | D | F | M | F | A | N | G | No | M | T | M |
| A/whooper swan/Shanxi/SX116/2020(H5N2) | L | V | A | S | G | S | No | D | F | M | S | A | S | G | GSEV | M | A | M |
| A/whooper swan/Shanxi/SX126/2020(H9N2) | L | V | A | S | G | A | No | D | Y | M | F | A | I | G | ESEV | M | S | M |
| A/whooper swan/Shanxi/SX166/2020(H5N8) | L | V | A | S | G | S | No | D | F | M | F | A | N | G | No | M | T | M |
| A/whooper swan/Shanxi/SX206/2020(H5N8) | L | V | A | S | G | S | No | D | F | M | F | A | N | G | No | M | T | M |
| A/whooper swan/Shanxi/SX216/2020(H5N8) | L | V | A | S | G | S | No | D | F | M | F | A | N | G | No | M | T | M |
| A/whooper swan/Shanxi/SX231/2020(H5N8) | L | V | A | S | G | S | No | D | F | M | F | A | N | G | No | M | T | M |
| A/whooper swan/Shanxi/SX251/2020(H5N8) | L | V | A | S | G | S | No | D | F | M | F | A | N | G | No | M | T | M |
| A/whooper swan/Shanxi/SX276/2020(H5N8) | L | V | A | S | G | S | No | D | F | M | F | A | N | G | No | M | T | M |
| A/whooper swan/Shanxi/SX291/2020(H5N8) | L | V | A | S | G | S | No | D | F | M | F | A | N | G | No | M | T | M |
| A/whooper swan/Shanxi/SX346/2020(H5N8) | L | V | A | S | G | S | No | D | F | M | F | A | N | G | No | M | T | M |
| A/whooper swan/Henan/SM1/2020(H5N8) | L | V | A | S | G | S | No | D | F | M | F | A | N | G | No | M | T | M |
| A/whooper swan/Henan/SM16/2020(H5N8) | L | V | A | S | G | S | No | D | F | M | F | A | N | G | No | M | T | M |
| A/whooper swan/Henan/SM31/2020(H5N8) | L | V | A | S | G | S | No | D | F | M | F | A | N | G | No | M | T | M |
| A/whooper swan/Henan/SM61/2020(H5N8) | L | V | A | S | G | S | No | D | F | M | F | A | N | G | No | M | T | M |
| A/whooper swan/Henan/SM76/2020(H5N8) | L | V | A | S | G | S | No | D | F | M | F | A | N | G | No | M | T | M |
| A/whooper swan/Henan/SM86/2020(H5N8) | L | V | A | S | G | S | No | D | F | M | F | A | N | G | No | M | T | M |
| A/whooper swan/Henan/SM111/2020(H5N8) | L | V | A | S | G | S | No | D | F | M | F | A | N | G | No | M | T | M |
| A/whooper swan/Henan/SMQ5/2020(H5N8) | L | V | A | S | G | S | No | D | F | M | F | A | N | G | No | M | T | M |
| A/whooper swan/Henan/SMQ6/2020(H5N8) | L | V | A | S | G | S | No | D | F | M | F | A | N | G | No | M | T | M |
| A/whooper swan/Henan/SMQ7/2020(H5N8) | L | V | A | S | G | S | No | D | F | M | F | A | N | G | No | M | T | M |
| A/whooper swan/Henan/SMQ9/2020(H5N8) | L | V | A | S | G | S | No | D | F | M | F | A | N | G | No | M | T | M |
| A/whooper swan/Henan/SMQ10/2020(H5N8) | L | V | A | S | G | S | No | D | F | M | F | A | N | G | No | M | T | M |
| A/eurasian eagle-owl/Henan/SMQ11/2020(H5N8) | L | V | A | S | G | S | No | D | F | M | F | A | N | G | No | M | T | M |
| A/tundra swan/Hubei/BQ2/2020(H5N8) | L | V | A | S | G | S | No | D | F | M | F | A | N | G | No | M | T | M |
| A/tundra swan/Hubei/BQ3/2020(H5N8) | L | V | A | S | G | S | No | D | F | M | F | A | N | G | No | M | T | M |
| A/tundra swan/Hubei/BQ4/2020(H5N8) | L | V | A | S | G | S | No | D | F | M | F | A | N | G | No | M | T | M |
| A/tundra swan/Hubei/BQ6/2020(H5N8) | L | V | A | S | G | S | No | D | F | M | F | A | N | G | No | M | T | M |
| A/tundra swan/Hubei/BQ7/2020(H5N8) | L | V | A | S | G | S | No | D | F | M | F | A | N | G | No | M | T | M |
| A/tundra swan/Hubei/BQ8/2020(H5N8) | L | V | A | S | G | S | No | D | F | M | F | A | N | G | No | M | T | M |
| A/tundra swan/Hubei/BQ9/2020(H5N8) | L | V | A | S | G | S | No | D | F | M | F | A | N | G | No | M | T | M |
| A/whiskered tern/Hubei/BQ10/2020(H5N8) | L | V | A | S | G | S | No | D | F | M | F | A | N | G | No | M | T | M |
| A/bean goose/Hubei/BQ11/2020(H5N8) | L | V | A | S | G | S | No | D | F | M | F | A | N | G | No | M | T | M |

**Appendix Table 7**. Related to Figure 1. Detailed information and genotyping of all sub-clade 2.3.4.4b H5 viruses (2020) analyzed in this study.

| **Strain name** | **Collection Date** | **Isolate ID** | **Country** | **Host** | **HA^*^** | **NA** | **PB2^#^** | **PB1** | **PA** | **NP** | **M** | **NS** |
| --- | --- | --- | --- | --- | --- | --- | --- | --- | --- | --- | --- | --- |
| A/whooper_swan/Shanxi/SX116/2020(H5N2) | 2020-Nov-10 | EPI_ISL_1822592 | China | Whooper swan | b1 | N2 | LPAIV | LPAIV | LPAIV | LPAIV | LPAIV | African HPAI H5 |
| A/chicken/Czech_Republic/1175-1/2020(H5N8) | 2020-Jan-17 | EPI_ISL_405391 | Czech Republic | Chicken | b1 | b1 (N8) | African HPAI H5 | LPAIV | African HPAI H5 | LPAIV | African HPAI H5 | African HPAI H5 |
| A/turkey/Czech_Republic/3071/2020(H5N8) | 2020-Feb-17 | EPI_ISL_418266 | Czech Republic | Turkey | b1 | b1 (N8) | African HPAI H5 | LPAIV | African HPAI H5 | LPAIV | African HPAI H5 | African HPAI H5 |
| A/buzzard/Germany-SN/AI00285/2020(H5N8) | 2020-Mar-20 | EPI_ISL_417414 | Germany | Buzzard | b1 | b1 (N8) | African HPAI H5 | LPAIV | African HPAI H5 | LPAIV | African HPAI H5 | African HPAI H5 |
| A/chicken/Germany-BW/AI00049/2020(H5N8) | 2020-Feb-06 | EPI_ISL_410291 | Germany | Chicken | b1 | b1 (N8) | African HPAI H5 | LPAIV | African HPAI H5 | LPAIV | African HPAI H5 | African HPAI H5 |
| A/chicken/Germany-SN/AI00276/2020(H5N8) | 2020-Mar-12 | EPI_ISL_415197 | Germany | Chicken | b1 | b1 (N8) | African HPAI H5 | LPAIV | African HPAI H5 | LPAIV | African HPAI H5 | African HPAI H5 |
| A/steamer_duck/Germany-SN/AI00346/2020(H5N8) | 2020-Mar-26 | EPI_ISL_419312 | Germany | Steamer duck | b1 | b1 (N8) | African HPAI H5 | LPAIV | African HPAI H5 | LPAIV | African HPAI H5 | African HPAI H5 |
| A/turkey/Germany-NI/AI00334/2020(H5N8) | 2020-Mar-20 | EPI_ISL_417415 | Germany | Turkey | b1 | b1 (N8) | African HPAI H5 | LPAIV | African HPAI H5 | LPAIV | African HPAI H5 | African HPAI H5 |
| A/turkey/Germany-ST/AI00352/2020(H5N8) | 2020-Mar-27 | EPI_ISL_419314 | Germany | Turkey | b1 | b1 (N8) | African HPAI H5 | LPAIV | African HPAI H5 | LPAIV | African HPAI H5 | African HPAI H5 |
| A/white-fronted_goose/Germany-BB/AI00018/2020(H5N8) | 2020-Jan-16 | EPI_ISL_404993 | Germany | White-fronted goose | b1 | b1 (N8) | African HPAI H5 | LPAIV | African HPAI H5 | LPAIV | African HPAI H5 | African HPAI H5 |
| A/Chicken/Hungary/18466/2020(H5N8) | 2020-Apr-14 | EPI_ISL_810970 | Hungary | Chicken | b1 | b1 (N8) | African HPAI H5 | LPAIV | African HPAI H5 | LPAIV | African HPAI H5 | African HPAI H5 |
| A/Chicken/Hungary/18467/2020(H5N8) | 2020-Apr-14 | EPI_ISL_811128 | Hungary | Chicken | b1 | b1 (N8) | African HPAI H5 | LPAIV | African HPAI H5 | LPAIV | African HPAI H5 | African HPAI H5 |
| A/Chicken/Hungary/19776/2020(H5N8) | 2020-Apr-21 | EPI_ISL_811134 | Hungary | Chicken | b1 | b1 (N8) | African HPAI H5 | LPAIV | African HPAI H5 | LPAIV | African HPAI H5 | African HPAI H5 |
| A/Chicken/Hungary/20227/2020(H5N8) | 2020-Apr-23 | EPI_ISL_811135 | Hungary | Chicken | b1 | b1 (N8) | African HPAI H5 | LPAIV | African HPAI H5 | LPAIV | African HPAI H5 | African HPAI H5 |
| A/Chicken/Hungary/21379/2020(H5N8) | 2020-Apr-30 | EPI_ISL_846583 | Hungary | Chicken | b1 | b1 (N8) | African HPAI H5 | LPAIV | African HPAI H5 | LPAIV | African HPAI H5 | African HPAI H5 |
| A/Chicken/Hungary/24596/2020(H5N8) | 2020-May-13 | EPI_ISL_846585 | Hungary | Chicken | b1 | b1 (N8) | African HPAI H5 | LPAIV | African HPAI H5 | LPAIV | African HPAI H5 | African HPAI H5 |
| A/Chicken/Hungary/29723/2020(H5N8) | 2020-Jun-05 | EPI_ISL_846586 | Hungary | Chicken | b1 | b1 (N8) | African HPAI H5 | LPAIV | African HPAI H5 | LPAIV | African HPAI H5 | African HPAI H5 |
| A/Duck/Hungary/14788/2020(H5N8) | 2020-Mar-25 | EPI_ISL_780096 | Hungary | Duck | b1 | b1 (N8) | African HPAI H5 | LPAIV | African HPAI H5 | LPAIV | African HPAI H5 | African HPAI H5 |
| A/Duck/Hungary/17806/2020(H5N8) | 2020-Apr-08 | EPI_ISL_804045 | Hungary | Duck | b1 | b1 (N8) | African HPAI H5 | LPAIV | African HPAI H5 | LPAIV | African HPAI H5 | African HPAI H5 |
| A/Duck/Hungary/17957/2020(H5N8) | 2020-Apr-09 | EPI_ISL_804047 | Hungary | Duck | b1 | b1 (N8) | African HPAI H5 | LPAIV | African HPAI H5 | LPAIV | African HPAI H5 | African HPAI H5 |
| A/Duck/Hungary/18358/2020(H5N8) | 2020-Apr-11 | EPI_ISL_808250 | Hungary | Duck | b1 | b1 (N8) | African HPAI H5 | LPAIV | African HPAI H5 | LPAIV | African HPAI H5 | African HPAI H5 |
| A/Duck/Hungary/18414/2020(H5N8) | 2020-Apr-14 | EPI_ISL_833579 | Hungary | Duck | b1 | b1 (N8) | African HPAI H5 | LPAIV | African HPAI H5 | LPAIV | African HPAI H5 | African HPAI H5 |
| A/Duck/Hungary/18444/2020(H5N8) | 2020-Apr-14 | EPI_ISL_810963 | Hungary | Duck | b1 | b1 (N8) | African HPAI H5 | LPAIV | African HPAI H5 | LPAIV | African HPAI H5 | African HPAI H5 |
| A/Goose/Hungary/15267/2020(H5N8) | 2020-Mar-26 | EPI_ISL_796011 | Hungary | Goose | b1 | b1 (N8) | African HPAI H5 | LPAIV | African HPAI H5 | LPAIV | African HPAI H5 | African HPAI H5 |
| A/Goose/Hungary/18325/2020(H5N8) | 2020-Apr-10 | EPI_ISL_833482 | Hungary | Goose | b1 | b1 (N8) | African HPAI H5 | LPAIV | African HPAI H5 | LPAIV | African HPAI H5 | African HPAI H5 |
| A/Goose/Hungary/18406/2020(H5N8) | 2020-Apr-14 | EPI_ISL_809658 | Hungary | Goose | b1 | b1 (N8) | African HPAI H5 | LPAIV | African HPAI H5 | LPAIV | African HPAI H5 | African HPAI H5 |
| A/Goose/Hungary/19118/2020(H5N8) | 2020-Apr-17 | EPI_ISL_833581 | Hungary | Goose | b1 | b1 (N8) | African HPAI H5 | LPAIV | African HPAI H5 | LPAIV | African HPAI H5 | African HPAI H5 |
| A/Goose/Hungary/19128/2020(H5N8) | 2020-Apr-17 | EPI_ISL_846584 | Hungary | Goose | b1 | b1 (N8) | African HPAI H5 | LPAIV | African HPAI H5 | LPAIV | African HPAI H5 | African HPAI H5 |
| A/Goose/Hungary/19953/2020(H5N8) | 2020-Apr-22 | EPI_ISL_833582 | Hungary | Goose | b1 | b1 (N8) | African HPAI H5 | LPAIV | African HPAI H5 | LPAIV | African HPAI H5 | African HPAI H5 |
| A/Goose/Hungary/19959/2020(H5N8) | 2020-Apr-22 | EPI_ISL_833583 | Hungary | Goose | b1 | b1 (N8) | African HPAI H5 | LPAIV | African HPAI H5 | LPAIV | African HPAI H5 | African HPAI H5 |
| A/Goose/Hungary/21737/2020(H5N8) | 2020-Apr-30 | EPI_ISL_811144 | Hungary | Goose | b1 | b1 (N8) | African HPAI H5 | LPAIV | African HPAI H5 | LPAIV | African HPAI H5 | African HPAI H5 |
| A/Goose/Hungary/22493/2020(H5N8) | 2020-May-04 | EPI_ISL_813599 | Hungary | Goose | b1 | b1 (N8) | African HPAI H5 | LPAIV | African HPAI H5 | LPAIV | African HPAI H5 | African HPAI H5 |
| A/Goose/Hungary/24021/2020(H5N8) | 2020-May-11 | EPI_ISL_813972 | Hungary | Goose | b1 | b1 (N8) | African HPAI H5 | LPAIV | African HPAI H5 | LPAIV | African HPAI H5 | African HPAI H5 |
| A/Mallard_duck/Hungary/17319/2020(H5N8) | 2020-Apr-07 | EPI_ISL_804043 | Hungary | Mallard duck | b1 | b1 (N8) | African HPAI H5 | LPAIV | African HPAI H5 | LPAIV | African HPAI H5 | African HPAI H5 |
| A/Mallard_duck/Hungary/18410/2020(H5N8) | 2020-Apr-14 | EPI_ISL_833485 | Hungary | Mallard duck | b1 | b1 (N8) | African HPAI H5 | LPAIV | African HPAI H5 | LPAIV | African HPAI H5 | African HPAI H5 |
| A/Pheasant/Hungary/18731/2020(H5N8) | 2020-Apr-19 | EPI_ISL_833487 | Hungary | Pheasan | b1 | b1 (N8) | African HPAI H5 | LPAIV | African HPAI H5 | LPAIV | African HPAI H5 | African HPAI H5 |
| A/turkey/Hungary/1020_20VIR749-1/2020(H5N8) | 2020-Jan-09 | EPI_ISL_419220 | Hungary | Turkey | b1 | b1 (N8) | African HPAI H5 | LPAIV | African HPAI H5 | LPAIV | African HPAI H5 | African HPAI H5 |
| A/Turkey/Hungary/19338/2020(H5N8) | 2020-Apr-18 | EPI_ISL_833488 | Hungary | Turkey | b1 | b1 (N8) | African HPAI H5 | LPAIV | African HPAI H5 | LPAIV | African HPAI H5 | African HPAI H5 |
| A/Turkey/Hungary/19394/2020(H5N8) | 2020-Apr-18 | EPI_ISL_833580 | Hungary | Turkey | b1 | b1 (N8) | African HPAI H5 | LPAIV | African HPAI H5 | LPAIV | African HPAI H5 | African HPAI H5 |
| A/Turkey/Hungary/21753/2020(H5N8) | 2020-Apr-30 | EPI_ISL_811146 | Hungary | Turkey | b1 | b1 (N8) | African HPAI H5 | LPAIV | African HPAI H5 | LPAIV | African HPAI H5 | African HPAI H5 |
| A/Turkey/Hungary/22494/2020(H5N8) | 2020-May-05 | EPI_ISL_813971 | Hungary | Turkey | b1 | b1 (N8) | African HPAI H5 | LPAIV | African HPAI H5 | LPAIV | African HPAI H5 | African HPAI H5 |
| A/chicken/Kagawa/11C/2020(H5N8) | 2020-Nov-04 | EPI_ISL_681286 | Japan | Chicken | b1 | b1 (N8) | African HPAI H5 | LPAIV | African HPAI H5 | LPAIV | African HPAI H5 | African HPAI H5 |
| A/environment/Kagoshima/KU-ngr-J2/2020(H5N8) | 2020-Nov-09 | EPI_ISL_682297 | Japan | Environment | b1 | b1 (N8) | African HPAI H5 | LPAIV | African HPAI H5 | LPAIV | African HPAI H5 | African HPAI H5 |
| A/Mandarin_duck/Kagoshima/KU-d57/2020(H5N8) | 2020-Dec-22 | EPI_ISL_1063533 | Japan | Mandarin duck | b1 | b1 (N8) | LPAIV | LPAIV | African HPAI H5 | LPAIV | African HPAI H5 | African HPAI H5 |
| A/northern_pintail/Hokkaido/M13/2020(H5N8) | 2020-Oct-24 | EPI_ISL_697771 | Japan | Northern pintail | b1 | b1 (N8) | African HPAI H5 | LPAIV | African HPAI H5 | LPAIV | African HPAI H5 | African HPAI H5 |
| A/chicken/Korea/H365/2020(H5N8) | 2020-Dec-01 | EPI_ISL_985180 | Korea | Chicken | b1 | b1 (N8) | African HPAI H5 | LPAIV | African HPAI H5 | LPAIV | African HPAI H5 | African HPAI H5 |
| A/chicken/Korea/H390/2020(H5N8) | 2020-Dec-06 | EPI_ISL_985182 | Korea | Chicken | b1 | b1 (N8) | LPAIV | LPAIV | LPAIV | LPAIV | African HPAI H5 | LPAIV |
| A/chicken/Korea/H441/2020(H5N8) | 2020-Dec-14 | EPI_ISL_985192 | Korea | Chicken | b1 | b1 (N8) | LPAIV | LPAIV | African HPAI H5 | LPAIV | African HPAI H5 | African HPAI H5 |
| A/chicken/Korea/H450/2020(H5N8) | 2020-Dec-14 | EPI_ISL_985193 | Korea | Chicken | b1 | b1 (N8) | African HPAI H5 | LPAIV | African HPAI H5 | LPAIV | African HPAI H5 | African HPAI H5 |
| A/chicken/Korea/H470/2020(H5N8) | 2020-Dec-16 | EPI_ISL_985195 | Korea | Chicken | b1 | b1 (N8) | LPAIV | LPAIV | LPAIV | LPAIV | African HPAI H5 | LPAIV |
| A/chicken/Korea/H491/2020(H5N8) | 2020-Dec-21 | EPI_ISL_985198 | Korea | Chicken | b1 | b1 (N8) | LPAIV | LPAIV | LPAIV | LPAIV | African HPAI H5 | LPAIV |
| A/chicken/Korea/H531/2020(H5N8) | 2020-Dec-28 | EPI_ISL_985209 | Korea | Chicken | b1 | b1 (N8) | LPAIV | LPAIV | LPAIV | LPAIV | African HPAI H5 | LPAIV |
| A/chicken/Korea/H532/2020(H5N8) | 2020-Dec-28 | EPI_ISL_985210 | Korea | Chicken | b1 | b1 (N8) | LPAIV | LPAIV | LPAIV | LPAIV | African HPAI H5 | LPAIV |
| A/chicken/Korea/H541/2020(H5N8) | 2020-Dec-29 | EPI_ISL_1009681 | Korea | Chicken | b1 | b1 (N8) | LPAIV | LPAIV | African HPAI H5 | LPAIV | African HPAI H5 | African HPAI H5 |
| A/duck/Korea/H007/2020(H5N8) | 2020-Dec-28 | EPI_ISL_1009679 | Korea | Duck | b1 | b1 (N8) | African HPAI H5 | LPAIV | African HPAI H5 | LPAIV | African HPAI H5 | African HPAI H5 |
| A/duck/Korea/H338/2020(H5N8) | 2020-Nov-26 | EPI_ISL_985179 | Korea | Duck | b1 | b1 (N8) | African HPAI H5 | LPAIV | African HPAI H5 | LPAIV | African HPAI H5 | African HPAI H5 |
| A/duck/Korea/H385/2020(H5N8) | 2020-Dec-04 | EPI_ISL_985181 | Korea | Duck | b1 | b1 (N8) | LPAIV | LPAIV | LPAIV | LPAIV | African HPAI H5 | LPAIV |
| A/duck/Korea/H419/2020(H5N8) | 2020-Dec-09 | EPI_ISL_985186 | Korea | Duck | b1 | b1 (N8) | African HPAI H5 | LPAIV | African HPAI H5 | LPAIV | African HPAI H5 | African HPAI H5 |
| A/duck/Korea/H432/2020(H5N8) | 2020-Dec-10 | EPI_ISL_985188 | Korea | Duck | b1 | b1 (N8) | LPAIV | LPAIV | African HPAI H5 | LPAIV | African HPAI H5 | African HPAI H5 |
| A/duck/Korea/H438/2020(H5N8) | 2020-Dec-11 | EPI_ISL_985189 | Korea | Duck | b1 | b1 (N8) | LPAIV | LPAIV | LPAIV | LPAIV | African HPAI H5 | African HPAI H5 |
| A/duck/Korea/H439/2020(H5N8) | 2020-Dec-11 | EPI_ISL_985190 | Korea | Duck | b1 | b1 (N8) | LPAIV | LPAIV | African HPAI H5 | LPAIV | African HPAI H5 | African HPAI H5 |
| A/duck/Korea/H509/2020(H5N8) | 2020-Dec-22 | EPI_ISL_985200 | Korea | Duck | b1 | b1 (N8) | LPAIV | LPAIV | LPAIV | LPAIV | African HPAI H5 | LPAIV |
| A/duck/Korea/H511/2020(H5N8) | 2020-Dec-22 | EPI_ISL_985201 | Korea | Duck | b1 | b1 (N8) | LPAIV | LPAIV | African HPAI H5 | LPAIV | African HPAI H5 | African HPAI H5 |
| A/duck/Korea/H514/2020(H5N8) | 2020-Dec-23 | EPI_ISL_985202 | Korea | Duck | b1 | b1 (N8) | LPAIV | LPAIV | African HPAI H5 | LPAIV | African HPAI H5 | African HPAI H5 |
| A/duck/Korea/H515/2020(H5N8) | 2020-Dec-23 | EPI_ISL_985203 | Korea | Duck | b1 | b1 (N8) | LPAIV | LPAIV | African HPAI H5 | LPAIV | African HPAI H5 | African HPAI H5 |
| A/duck/Korea/H516/2020(H5N8) | 2020-Dec-23 | EPI_ISL_985204 | Korea | Duck | b1 | b1 (N8) | African HPAI H5 | LPAIV | African HPAI H5 | LPAIV | African HPAI H5 | African HPAI H5 |
| A/duck/Korea/H528/2020(H5N8) | 2020-Dec-27 | EPI_ISL_985208 | Korea | Duck | b1 | b1 (N8) | LPAIV | LPAIV | LPAIV | LPAIV | African HPAI H5 | LPAIV |
| A/duck/Korea/H548/2020(H5N8) | 2020-Dec-30 | EPI_ISL_1009684 | Korea | Duck | b1 | b1 (N8) | LPAIV | LPAIV | African HPAI H5 | LPAIV | African HPAI H5 | African HPAI H5 |
| A/Madarin_duck/Korea/K20-551-4/2020(H5N8) | 2020-Oct-24 | EPI_ISL_666687 | Korea | Madarin duck | b1 | b1 (N8) | African HPAI H5 | LPAIV | African HPAI H5 | LPAIV | African HPAI H5 | African HPAI H5 |
| A/Mandarin_duck/Korea/H242/2020(H5N8) | 2020-Oct-21 | EPI_ISL_631824 | Korea | Mandarin duck | b1 | b1 (N8) | African HPAI H5 | LPAIV | African HPAI H5 | LPAIV | African HPAI H5 | African HPAI H5 |
| A/mandarin_duck/Korea/WA831/2020(H5N8) | 2020-Nov-25 | EPI_ISL_1009699 | Korea | Mandarin duck | b1 | b1 (N8) | LPAIV | LPAIV | African HPAI H5 | LPAIV | African HPAI H5 | African HPAI H5 |
| A/mandarin_duck/Korea/WA857/2020(H5N8) | 2020-Nov-26 | EPI_ISL_1009701 | Korea | Mandarin duck | b1 | b1 (N8) | LPAIV | LPAIV | African HPAI H5 | LPAIV | African HPAI H5 | African HPAI H5 |
| A/mandarin_duck/Korea/WA877/2020(H5N8) | 2020-Dec-01 | EPI_ISL_1009702 | Korea | Mandarin duck | b1 | b1 (N8) | African HPAI H5 | LPAIV | African HPAI H5 | LPAIV | African HPAI H5 | African HPAI H5 |
| A/mandarin_duck/Korea/WA899/2020(H5N8) | 2020-Dec-01 | EPI_ISL_1009704 | Korea | Mandarin duck | b1 | b1 (N8) | LPAIV | LPAIV | African HPAI H5 | LPAIV | African HPAI H5 | African HPAI H5 |
| A/mandarin_duck/Korea/WA913/2020(H5N8) | 2020-Dec-01 | EPI_ISL_1009705 | Korea | Mandarin duck | b1 | b1 (N8) | African HPAI H5 | LPAIV | African HPAI H5 | LPAIV | African HPAI H5 | African HPAI H5 |
| A/mandarin_duck/Korea/WB80/2020(H5N8) | 2020-Nov-10 | EPI_ISL_1009696 | Korea | Mandarin duck | b1 | b1 (N8) | African HPAI H5 | LPAIV | African HPAI H5 | LPAIV | African HPAI H5 | African HPAI H5 |
| A/quail/Korea/H394/2020(H5N8) | 2020-Dec-07 | EPI_ISL_985183 | Korea | Quail | b1 | b1 (N8) | LPAIV | LPAIV | African HPAI H5 | LPAIV | African HPAI H5 | African HPAI H5 |
| A/quail/Korea/H412/2020(H5N8) | 2020-Dec-08 | EPI_ISL_985185 | Korea | Quail | b1 | b1 (N8) | LPAIV | LPAIV | LPAIV | LPAIV | African HPAI H5 | LPAIV |
| A/spot-billed_duck/Korea/WA612/2020(H5N8) | 2020-Nov-03 | EPI_ISL_1009695 | Korea | Spot-billed duck | b1 | b1 (N8) | African HPAI H5 | LPAIV | African HPAI H5 | LPAIV | African HPAI H5 | African HPAI H5 |
| A/spot-billed_duck/Korea/WA854/2020(H5N8) | 2020-Nov-26 | EPI_ISL_1009700 | Korea | Spot-billed duck | b1 | b1 (N8) | LPAIV | LPAIV | African HPAI H5 | LPAIV | African HPAI H5 | African HPAI H5 |
| A/spot-billed_duck/Korea/WA889/2020(H5N8) | 2020-Dec-01 | EPI_ISL_1009703 | Korea | Spot-billed duck | b1 | b1 (N8) | LPAIV | LPAIV | African HPAI H5 | LPAIV | African HPAI H5 | African HPAI H5 |
| A/wild_bird/Korea/H357/2020(H5N8) | 2020-Nov-23 | EPI_ISL_1009697 | Korea | Wild bird | b1 | b1 (N8) | LPAIV | LPAIV | African HPAI H5 | LPAIV | African HPAI H5 | African HPAI H5 |
| A/wild_bird/Korea/H379/2020(H5N8) | 2020-Dec-02 | EPI_ISL_1009706 | Korea | Wild bird | b1 | b1 (N8) | LPAIV | LPAIV | LPAIV | LPAIV | African HPAI H5 | LPAIV |
| A/chicken/Poland/003/2020(H5N8) | 2020-Jan-02 | EPI_ISL_525440 | Poland | Chicken | b1 | b1 (N8) | African HPAI H5 | LPAIV | African HPAI H5 | LPAIV | African HPAI H5 | African HPAI H5 |
| A/chicken/Poland/004/2020(H5N8) | 2020-Jan-02 | EPI_ISL_525441 | Poland | Chicken | b1 | b1 (N8) | African HPAI H5 | LPAIV | African HPAI H5 | LPAIV | African HPAI H5 | African HPAI H5 |
| A/chicken/Poland/054/2020(H5N8) | 2020-Jan-17 | EPI_ISL_525444 | Poland | Chicken | b1 | b1 (N8) | African HPAI H5 | LPAIV | African HPAI H5 | LPAIV | African HPAI H5 | African HPAI H5 |
| A/domestic_duck/Poland/219/2020(H5N8) | 2020-Feb-20 | EPI_ISL_525449 | Poland | Domestic duck | b1 | b1 (N8) | African HPAI H5 | LPAIV | African HPAI H5 | LPAIV | African HPAI H5 | African HPAI H5 |
| A/domestic_duck/Poland/221/2020(H5N8) | 2020-Feb-20 | EPI_ISL_525450 | Poland | Domestic duck | b1 | b1 (N8) | African HPAI H5 | LPAIV | African HPAI H5 | LPAIV | African HPAI H5 | African HPAI H5 |
| A/domestic_duck/Poland/222/2020(H5N8) | 2020-Feb-21 | EPI_ISL_525451 | Poland | Domestic duck | b1 | b1 (N8) | African HPAI H5 | LPAIV | African HPAI H5 | LPAIV | African HPAI H5 | African HPAI H5 |
| A/domestic_duck/Poland/223/2020(H5N8) | 2020-Feb-21 | EPI_ISL_525452 | Poland | Domestic duck | b1 | b1 (N8) | African HPAI H5 | LPAIV | African HPAI H5 | LPAIV | African HPAI H5 | African HPAI H5 |
| A/domestic_duck/Poland/229/2020(H5N8) | 2020-Feb-22 | EPI_ISL_525453 | Poland | Domestic duck | b1 | b1 (N8) | African HPAI H5 | LPAIV | African HPAI H5 | LPAIV | African HPAI H5 | African HPAI H5 |
| A/domestic_duck/Poland/230/2020(H5N8) | 2020-Feb-22 | EPI_ISL_525454 | Poland | Domestic duck | b1 | b1 (N8) | African HPAI H5 | LPAIV | African HPAI H5 | LPAIV | African HPAI H5 | African HPAI H5 |
| A/domestic_duck/Poland/237/2020(H5N8) | 2020-Feb-24 | EPI_ISL_525455 | Poland | Domestic duck | b1 | b1 (N8) | African HPAI H5 | LPAIV | African HPAI H5 | LPAIV | African HPAI H5 | African HPAI H5 |
| A/domestic_duck/Poland/263/2020(H5N8) | 2020-Feb-26 | EPI_ISL_525464 | Poland | Domestic duck | b1 | b1 (N8) | African HPAI H5 | LPAIV | African HPAI H5 | LPAIV | African HPAI H5 | African HPAI H5 |
| A/domestic_duck/Poland/271/2020(H5N8) | 2020-Feb-29 | EPI_ISL_525465 | Poland | Domestic duck | b1 | b1 (N8) | African HPAI H5 | LPAIV | African HPAI H5 | LPAIV | African HPAI H5 | African HPAI H5 |
| A/domestic_duck/Poland/285/2020(H5N8) | 2020-Mar-03 | EPI_ISL_525463 | Poland | Domestic duck | b1 | b1 (N8) | African HPAI H5 | LPAIV | African HPAI H5 | LPAIV | African HPAI H5 | African HPAI H5 |
| A/domestic_goose/Poland/028/2020(H5N8) | 2020-Jan-12 | EPI_ISL_525443 | Poland | Domestic goose | b1 | b1 (N8) | African HPAI H5 | LPAIV | African HPAI H5 | LPAIV | African HPAI H5 | African HPAI H5 |
| A/domestic_goose/Poland/274/2020(H5N8) | 2020-Mar-01 | EPI_ISL_525462 | Poland | Domestic goose | b1 | b1 (N8) | African HPAI H5 | LPAIV | African HPAI H5 | LPAIV | African HPAI H5 | African HPAI H5 |
| A/hawk/Poland/003/2020(H5N8) | 2020-Jan-06 | EPI_ISL_405813 | Poland | Hawk | b1 | b1 (N8) | African HPAI H5 | LPAIV | African HPAI H5 | Null | African HPAI H5 | African HPAI H5 |
| A/laying_hen/Poland/002/2020(H5N8) | 2020-Jan-01 | EPI_ISL_525439 | Poland | Laying hen | b1 | b1 (N8) | African HPAI H5 | LPAIV | African HPAI H5 | LPAIV | African HPAI H5 | African HPAI H5 |
| A/laying_hen/Poland/095/2020(H5N8) | 2020-Jan-27 | EPI_ISL_525446 | Poland | Laying hen | b1 | b1 (N8) | African HPAI H5 | LPAIV | African HPAI H5 | LPAIV | African HPAI H5 | African HPAI H5 |
| A/turkey/Poland/027/2020(H5N8) | 2020-Jan-09 | EPI_ISL_525442 | Poland | Turkey | b1 | b1 (N8) | African HPAI H5 | LPAIV | African HPAI H5 | LPAIV | African HPAI H5 | African HPAI H5 |
| A/turkey/Poland/079/2020(H5N8) | 2020-Jan-25 | EPI_ISL_525445 | Poland | Turkey | b1 | b1 (N8) | African HPAI H5 | LPAIV | African HPAI H5 | LPAIV | African HPAI H5 | African HPAI H5 |
| A/turkey/Poland/096/2020(H5N8) | 2020-Jan-28 | EPI_ISL_525447 | Poland | Turkey | b1 | b1 (N8) | African HPAI H5 | LPAIV | African HPAI H5 | LPAIV | African HPAI H5 | African HPAI H5 |
| A/turkey/Poland/182/2020(H5N8) | 2020-Feb-07 | EPI_ISL_525448 | Poland | Turkey | b1 | b1 (N8) | African HPAI H5 | LPAIV | African HPAI H5 | LPAIV | African HPAI H5 | African HPAI H5 |
| A/Anser_albifrons/Belgium/11956_005/2020(H5N8) | 2020-Nov-07 | EPI_ISL_661313 | Belgium | Anser albifrons | b2 | b2 (N8) | Eurasian HPAI H5 | Eurasian HPAI H5 | Eurasian HPAI H5 | Eurasian HPAI H5 | Eurasian HPAI H5 | Eurasian HPAI H5 |
| A/Gallus_gallus/Belgium/12168_002/2020(H5N5) | 2020-Nov-18 | EPI_ISL_660264 | Belgium | Gallus gallus | b2 | N5 | Eurasian HPAI H5 | Eurasian HPAI H5 | LPAIV | Eurasian HPAI H5 | Eurasian HPAI H5 | Eurasian HPAI H5 |
| A/Numenius_arquata/Belgium/11956_003/2020(H5N8) | 2020-Nov-07 | EPI_ISL_664102 | Belgium | Numenius arquata | b2 | b2 (N8) | Eurasian HPAI H5 | Null | Eurasian HPAI H5 | Eurasian HPAI H5 | Eurasian HPAI H5 | Eurasian HPAI H5 |
| A/bean_goose/Hubei/BQ11/2020(H5N8) | 2020-Nov-16 | EPI_ISL_2713372 | China | Bean goose | b2 | b2 (N8) | Eurasian HPAI H5 | Eurasian HPAI H5 | Eurasian HPAI H5 | Eurasian HPAI H5 | Eurasian HPAI H5 | Eurasian HPAI H5 |
| A/common_teal/Shaanxi/SXY1-1/2020(H5N8) | 2020-Nov-10 | EPI_ISL_1760448 | China | Common teal | b2 | b2 (N8) | Eurasian HPAI H5 | Eurasian HPAI H5 | Eurasian HPAI H5 | Eurasian HPAI H5 | Eurasian HPAI H5 | Eurasian HPAI H5 |
| A/eurasian_eagle-owl/Henan/SMQ11/2020(H5N8) | 2020-Nov-11 | EPI_ISL_1939617 | China | Eurasian eagle-owl | b2 | b2 (N8) | Eurasian HPAI H5 | Eurasian HPAI H5 | Eurasian HPAI H5 | Eurasian HPAI H5 | Eurasian HPAI H5 | Eurasian HPAI H5 |
| A/mute_swan/Inner_Mongolia/W2-1/2020(H5N8) | 2020-Oct-17 | EPI_ISL_625672 | China | Mute swan | b2 | b2 (N8) | Eurasian HPAI H5 | Eurasian HPAI H5 | Eurasian HPAI H5 | Eurasian HPAI H5 | Eurasian HPAI H5 | Eurasian HPAI H5 |
| A/tundra_swan/Hubei/BQ2/2020(H5N8) | 2020-Nov-04 | EPI_ISL_2685842 | China | Tundra swan | b2 | b2 (N8) | Eurasian HPAI H5 | Eurasian HPAI H5 | Eurasian HPAI H5 | Eurasian HPAI H5 | Eurasian HPAI H5 | Eurasian HPAI H5 |
| A/tundra_swan/Hubei/BQ3/2020(H5N8) | 2020-Nov-09 | EPI_ISL_2685846 | China | Tundra swan | b2 | b2 (N8) | Eurasian HPAI H5 | Eurasian HPAI H5 | Eurasian HPAI H5 | Eurasian HPAI H5 | Eurasian HPAI H5 | Eurasian HPAI H5 |
| A/tundra_swan/Hubei/BQ4/2020(H5N8) | 2020-Nov-11 | EPI_ISL_2685847 | China | Tundra swan | b2 | b2 (N8) | Eurasian HPAI H5 | Eurasian HPAI H5 | Eurasian HPAI H5 | Eurasian HPAI H5 | Eurasian HPAI H5 | Eurasian HPAI H5 |
| A/tundra_swan/Hubei/BQ6/2020(H5N8) | 2020-Nov-16 | EPI_ISL_2685848 | China | Tundra swan | b2 | b2 (N8) | Eurasian HPAI H5 | Eurasian HPAI H5 | Eurasian HPAI H5 | Eurasian HPAI H5 | Eurasian HPAI H5 | Eurasian HPAI H5 |
| A/tundra_swan/Hubei/BQ7/2020(H5N8) | 2020-Nov-16 | EPI_ISL_2685900 | China | Tundra swan | b2 | b2 (N8) | Eurasian HPAI H5 | Eurasian HPAI H5 | Eurasian HPAI H5 | Eurasian HPAI H5 | Eurasian HPAI H5 | Eurasian HPAI H5 |
| A/tundra_swan/Hubei/BQ8/2020(H5N8) | 2020-Nov-16 | EPI_ISL_2685947 | China | Tundra swan | b2 | b2 (N8) | Eurasian HPAI H5 | Eurasian HPAI H5 | Eurasian HPAI H5 | Eurasian HPAI H5 | Eurasian HPAI H5 | Eurasian HPAI H5 |
| A/tundra_swan/Hubei/BQ9/2020(H5N8) | 2020-Nov-16 | EPI_ISL_2713170 | China | Tundra swan | b2 | b2 (N8) | Eurasian HPAI H5 | Eurasian HPAI H5 | Eurasian HPAI H5 | Eurasian HPAI H5 | Eurasian HPAI H5 | Eurasian HPAI H5 |
| A/whiskered_tern/Hubei/BQ10/2020(H5N8) | 2020-Nov-16 | EPI_ISL_2713314 | China | Whiskered tern | b2 | b2 (N8) | Eurasian HPAI H5 | Eurasian HPAI H5 | Eurasian HPAI H5 | Eurasian HPAI H5 | Eurasian HPAI H5 | Eurasian HPAI H5 |
| A/whooper_swan/Henan/SM1/2020(H5N8) | 2020-Nov-10 | EPI_ISL_1937884 | China | Whooper swan | b2 | b2 (N8) | Eurasian HPAI H5 | Eurasian HPAI H5 | Eurasian HPAI H5 | Eurasian HPAI H5 | Eurasian HPAI H5 | Eurasian HPAI H5 |
| A/whooper_swan/Henan/SM111/2020(H5N8) | 2020-Nov-10 | EPI_ISL_1938309 | China | Whooper swan | b2 | b2 (N8) | Eurasian HPAI H5 | Eurasian HPAI H5 | Eurasian HPAI H5 | Eurasian HPAI H5 | Eurasian HPAI H5 | Eurasian HPAI H5 |
| A/whooper_swan/Henan/SM16/2020(H5N8) | 2020-Nov-10 | EPI_ISL_1937889 | China | Whooper swan | b2 | b2 (N8) | Eurasian HPAI H5 | Eurasian HPAI H5 | Eurasian HPAI H5 | Eurasian HPAI H5 | Eurasian HPAI H5 | Eurasian HPAI H5 |
| A/whooper_swan/Henan/SM31/2020(H5N8) | 2020-Nov-10 | EPI_ISL_1937890 | China | Whooper swan | b2 | b2 (N8) | Eurasian HPAI H5 | Eurasian HPAI H5 | Eurasian HPAI H5 | Eurasian HPAI H5 | Eurasian HPAI H5 | Eurasian HPAI H5 |
| A/whooper_swan/Henan/SM61/2020(H5N8) | 2020-Nov-10 | EPI_ISL_1937891 | China | Whooper swan | b2 | b2 (N8) | Eurasian HPAI H5 | Eurasian HPAI H5 | Eurasian HPAI H5 | Eurasian HPAI H5 | Eurasian HPAI H5 | Eurasian HPAI H5 |
| A/whooper_swan/Henan/SM76/2020(H5N8) | 2020-Nov-10 | EPI_ISL_1937892 | China | Whooper swan | b2 | b2 (N8) | Eurasian HPAI H5 | Eurasian HPAI H5 | Eurasian HPAI H5 | Eurasian HPAI H5 | Eurasian HPAI H5 | Eurasian HPAI H5 |
| A/whooper_swan/Henan/SM86/2020(H5N8) | 2020-Nov-10 | EPI_ISL_1938307 | China | Whooper swan | b2 | b2 (N8) | Eurasian HPAI H5 | Eurasian HPAI H5 | Eurasian HPAI H5 | Eurasian HPAI H5 | Eurasian HPAI H5 | Eurasian HPAI H5 |
| A/whooper_swan/Henan/SMQ10/2020(H5N8) | 2020-Nov-10 | EPI_ISL_1939080 | China | Whooper swan | b2 | b2 (N8) | Eurasian HPAI H5 | Eurasian HPAI H5 | Eurasian HPAI H5 | Eurasian HPAI H5 | Eurasian HPAI H5 | Eurasian HPAI H5 |
| A/whooper_swan/Henan/SMQ5/2020(H5N8) | 2020-Nov-02 | EPI_ISL_1829205 | China | Whooper swan | b2 | b2 (N8) | Eurasian HPAI H5 | Eurasian HPAI H5 | Eurasian HPAI H5 | Eurasian HPAI H5 | Eurasian HPAI H5 | Eurasian HPAI H5 |
| A/whooper_swan/Henan/SMQ6/2020(H5N8) | 2020-Nov-06 | EPI_ISL_1834185 | China | Whooper swan | b2 | b2 (N8) | Eurasian HPAI H5 | Eurasian HPAI H5 | Eurasian HPAI H5 | Eurasian HPAI H5 | Eurasian HPAI H5 | Eurasian HPAI H5 |
| A/whooper_swan/Henan/SMQ7/2020(H5N8) | 2020-Nov-09 | EPI_ISL_1937883 | China | Whooper swan | b2 | b2 (N8) | Eurasian HPAI H5 | Eurasian HPAI H5 | Eurasian HPAI H5 | Eurasian HPAI H5 | Eurasian HPAI H5 | Eurasian HPAI H5 |
| A/whooper_swan/Henan/SMQ9/2020(H5N8) | 2020-Nov-10 | EPI_ISL_1938478 | China | Whooper swan | b2 | b2 (N8) | Eurasian HPAI H5 | Eurasian HPAI H5 | Eurasian HPAI H5 | Eurasian HPAI H5 | Eurasian HPAI H5 | Eurasian HPAI H5 |
| A/whooper_swan/Inner_Mongolia/W1-1/2020(H5N8) | 2020-Oct-17 | EPI_ISL_625671 | China | Whooper swan | b2 | b2 (N8) | Eurasian HPAI H5 | Eurasian HPAI H5 | Eurasian HPAI H5 | Eurasian HPAI H5 | Eurasian HPAI H5 | Eurasian HPAI H5 |
| A/whooper_swan/Shaanxi/SXY2-1/2020(H5N8) | 2020-Nov-10 | EPI_ISL_1760450 | China | Whooper swan | b2 | b2 (N8) | Eurasian HPAI H5 | Eurasian HPAI H5 | Eurasian HPAI H5 | Eurasian HPAI H5 | Eurasian HPAI H5 | Eurasian HPAI H5 |
| A/whooper_swan/Shaanxi/SXY26/2020(H5N8) | 2020-Nov-09 | EPI_ISL_1760446 | China | Whooper swan | b2 | b2 (N8) | Eurasian HPAI H5 | Eurasian HPAI H5 | Eurasian HPAI H5 | Eurasian HPAI H5 | Eurasian HPAI H5 | Eurasian HPAI H5 |
| A/whooper_swan/Shaanxi/SXY66/2020(H5N8) | 2020-Nov-10 | EPI_ISL_1760447 | China | Whooper swan | b2 | b2 (N8) | Eurasian HPAI H5 | Eurasian HPAI H5 | Eurasian HPAI H5 | Eurasian HPAI H5 | Eurasian HPAI H5 | Eurasian HPAI H5 |
| A/whooper_swan/Shanxi/SX106/2020(H5N8) | 2020-Nov-10 | EPI_ISL_1760454 | China | Whooper swan | b2 | b2 (N8) | Eurasian HPAI H5 | Eurasian HPAI H5 | Eurasian HPAI H5 | Eurasian HPAI H5 | Eurasian HPAI H5 | Eurasian HPAI H5 |
| A/whooper_swan/Shanxi/SX16/2020(H5N8) | 2020-Nov-10 | EPI_ISL_1760451 | China | Whooper swan | b2 | b2 (N8) | Eurasian HPAI H5 | Eurasian HPAI H5 | Eurasian HPAI H5 | Eurasian HPAI H5 | Eurasian HPAI H5 | Eurasian HPAI H5 |
| A/whooper_swan/Shanxi/SX166/2020(H5N8) | 2020-Nov-10 | EPI_ISL_1822592 | China | Whooper swan | b2 | b2 (N8) | Eurasian HPAI H5 | Eurasian HPAI H5 | Eurasian HPAI H5 | Eurasian HPAI H5 | Eurasian HPAI H5 | Eurasian HPAI H5 |
| A/whooper_swan/Shanxi/SX206/2020(H5N8) | 2020-Nov-10 | EPI_ISL_1822593 | China | Whooper swan | b2 | b2 (N8) | Eurasian HPAI H5 | Eurasian HPAI H5 | Eurasian HPAI H5 | Eurasian HPAI H5 | Eurasian HPAI H5 | Eurasian HPAI H5 |
| A/whooper_swan/Shanxi/SX216/2020(H5N8) | 2020-Nov-10 | EPI_ISL_1822594 | China | Whooper swan | b2 | b2 (N8) | Eurasian HPAI H5 | Eurasian HPAI H5 | Eurasian HPAI H5 | Eurasian HPAI H5 | Eurasian HPAI H5 | Eurasian HPAI H5 |
| A/whooper_swan/Shanxi/SX231/2020(H5N8) | 2020-Nov-10 | EPI_ISL_1822595 | China | Whooper swan | b2 | b2 (N8) | Eurasian HPAI H5 | Eurasian HPAI H5 | Eurasian HPAI H5 | Eurasian HPAI H5 | Eurasian HPAI H5 | Eurasian HPAI H5 |
| A/whooper_swan/Shanxi/SX251/2020(H5N8) | 2020-Nov-10 | EPI_ISL_1822596 | China | Whooper swan | b2 | b2 (N8) | Eurasian HPAI H5 | Eurasian HPAI H5 | Eurasian HPAI H5 | Eurasian HPAI H5 | Eurasian HPAI H5 | Eurasian HPAI H5 |
| A/whooper_swan/Shanxi/SX276/2020(H5N8) | 2020-Nov-10 | EPI_ISL_1822597 | China | Whooper swan | b2 | b2 (N8) | Eurasian HPAI H5 | Eurasian HPAI H5 | Eurasian HPAI H5 | Eurasian HPAI H5 | Eurasian HPAI H5 | Eurasian HPAI H5 |
| A/whooper_swan/Shanxi/SX291/2020(H5N8) | 2020-Nov-10 | EPI_ISL_1822598 | China | Whooper swan | b2 | b2 (N8) | Eurasian HPAI H5 | Eurasian HPAI H5 | Eurasian HPAI H5 | Eurasian HPAI H5 | Eurasian HPAI H5 | Eurasian HPAI H5 |
| A/whooper_swan/Shanxi/SX31/2020(H5N8) | 2020-Nov-10 | EPI_ISL_1760452 | China | Whooper swan | b2 | b2 (N8) | Eurasian HPAI H5 | Eurasian HPAI H5 | Eurasian HPAI H5 | Eurasian HPAI H5 | Eurasian HPAI H5 | Eurasian HPAI H5 |
| A/whooper_swan/Shanxi/SX346/2020(H5N8) | 2020-Nov-10 | EPI_ISL_1822599 | China | Whooper swan | b2 | b2 (N8) | Eurasian HPAI H5 | Eurasian HPAI H5 | Eurasian HPAI H5 | Eurasian HPAI H5 | Eurasian HPAI H5 | Eurasian HPAI H5 |
| A/whooper_swan/Shanxi/SX56/2020(H5N8) | 2020-Nov-10 | EPI_ISL_1760453 | China | Whooper swan | b2 | b2 (N8) | Eurasian HPAI H5 | Eurasian HPAI H5 | Eurasian HPAI H5 | Eurasian HPAI H5 | Eurasian HPAI H5 | Eurasian HPAI H5 |
| A/barnacle_goose/Denmark/14138-1/2020-11-04(H5N8) | 2020-Nov-04 | EPI_ISL_644824 | Denmark | Barnacle goose | b2 | b2 (N8) | Eurasian HPAI H5 | Eurasian HPAI H5 | Eurasian HPAI H5 | Eurasian HPAI H5 | Eurasian HPAI H5 | Eurasian HPAI H5 |
| A/peregrine_falcon/Denmark/13776-1/2020-10-30(H5N5) | 2020-Oct-30 | EPI_ISL_644737 | Denmark | Peregrine falcon | b2 | N5 | Null | Eurasian HPAI H5 | LPAIV | LPAIV | Eurasian HPAI H5 | Eurasian HPAI H5 |
| A/brent_goose/England/095684/2020(H5N5) | 2020-Nov-12 | EPI_ISL_1123358 | England | Brent goose | b2 | N5 | Eurasian HPAI H5 | Eurasian HPAI H5 | Eurasian HPAI H5 | Eurasian HPAI H5 | Eurasian HPAI H5 | Eurasian HPAI H5 |
| A/brent_goose/England/233339/2020(H5N8) | 2020-Nov-08 | EPI_ISL_1123357 | England | Brent goose | b2 | b2 (N8) | Eurasian HPAI H5 | Eurasian HPAI H5 | Eurasian HPAI H5 | Eurasian HPAI H5 | Eurasian HPAI H5 | Eurasian HPAI H5 |
| A/Canada_goose/England/032697/2020(H5N8) | 2020-Nov-03 | EPI_ISL_710506 | England | Canada goose | b2 | b2 (N8) | Eurasian HPAI H5 | Eurasian HPAI H5 | Eurasian HPAI H5 | Eurasian HPAI H5 | Eurasian HPAI H5 | Eurasian HPAI H5 |
| A/chicken/England/030720/2020(H5N8) | 2020-Nov-02 | EPI_ISL_626652 | England | Chicken | b2 | b2 (N8) | Eurasian HPAI H5 | Eurasian HPAI H5 | Eurasian HPAI H5 | Eurasian HPAI H5 | Eurasian HPAI H5 | Eurasian HPAI H5 |
| A/chicken/England/033708/2020(H5N8) | 2020-Nov-09 | EPI_ISL_710509 | England | Chicken | b2 | b2 (N8) | Eurasian HPAI H5 | Eurasian HPAI H5 | Eurasian HPAI H5 | Eurasian HPAI H5 | Eurasian HPAI H5 | Eurasian HPAI H5 |
| A/chicken/England/037052/2020(H5N8) | 2020-Nov-19 | EPI_ISL_710511 | England | Chicken | b2 | b2 (N8) | Eurasian HPAI H5 | Eurasian HPAI H5 | Eurasian HPAI H5 | Eurasian HPAI H5 | Eurasian HPAI H5 | Eurasian HPAI H5 |
| A/chicken/England/043683/2020(H5N8) | 2020-Dec-17 | EPI_ISL_1123350 | England | Chicken | b2 | b2 (N8) | Eurasian HPAI H5 | Eurasian HPAI H5 | Eurasian HPAI H5 | Eurasian HPAI H5 | Eurasian HPAI H5 | Eurasian HPAI H5 |
| A/chicken/England/045984/2020(H5N8) | 2020-Dec-24 | EPI_ISL_1123352 | England | Chicken | b2 | b2 (N8) | Eurasian HPAI H5 | Eurasian HPAI H5 | Eurasian HPAI H5 | Eurasian HPAI H5 | Eurasian HPAI H5 | Eurasian HPAI H5 |
| A/chicken/England/046491/2020(H5N8) | 2020-Dec-28 | EPI_ISL_1123354 | England | Chicken | b2 | b2 (N8) | Eurasian HPAI H5 | Eurasian HPAI H5 | Eurasian HPAI H5 | Eurasian HPAI H5 | Eurasian HPAI H5 | Null |
| A/chicken/Northern_Ireland/2020-17671_21VIR113-11/2020(H5N8) | 2020-Dec-31 | EPI_ISL_995172 | England | Chicken | b2 | b2 (N8) | Eurasian HPAI H5 | Eurasian HPAI H5 | Eurasian HPAI H5 | Eurasian HPAI H5 | Eurasian HPAI H5 | Eurasian HPAI H5 |
| A/chicken/Scotland/043405/2020(H5N8) | 2020-Dec-15 | EPI_ISL_1123263 | England | Chicken | b2 | b2 (N8) | Eurasian HPAI H5 | Eurasian HPAI H5 | Eurasian HPAI H5 | Eurasian HPAI H5 | Eurasian HPAI H5 | Eurasian HPAI H5 |
| A/duck/England/043628/2020(H5N8) | 2020-Dec-17 | EPI_ISL_1123351 | England | Duck | b2 | b2 (N8) | Eurasian HPAI H5 | Eurasian HPAI H5 | Eurasian HPAI H5 | Eurasian HPAI H5 | Eurasian HPAI H5 | Eurasian HPAI H5 |
| A/duck/England/046311/2020(H5N8) | 2020-Dec-26 | EPI_ISL_1123353 | England | Duck | b2 | b2 (N8) | Eurasian HPAI H5 | Eurasian HPAI H5 | Eurasian HPAI H5 | Eurasian HPAI H5 | Eurasian HPAI H5 | Eurasian HPAI H5 |
| A/falcon/England/041976/2020(H5N8) | 2020-Dec-14 | EPI_ISL_766056 | England | Falcon | b2 | b2 (N8) | Eurasian HPAI H5 | Eurasian HPAI H5 | Eurasian HPAI H5 | Eurasian HPAI H5 | Eurasian HPAI H5 | Eurasian HPAI H5 |
| A/Greylag_goose/England/032698/2020(H5N8) | 2020-Nov-03 | EPI_ISL_710507 | England | Greylag goose | b2 | b2 (N8) | Eurasian HPAI H5 | Eurasian HPAI H5 | Eurasian HPAI H5 | Eurasian HPAI H5 | Eurasian HPAI H5 | Eurasian HPAI H5 |
| A/Greylag_goose/England/033100/2020(H5N8) | 2020-Oct-30 | EPI_ISL_710508 | England | Greylag goose | b2 | Null | Eurasian HPAI H5 | Eurasian HPAI H5 | Eurasian HPAI H5 | Eurasian HPAI H5 | Eurasian HPAI H5 | Eurasian HPAI H5 |
| A/mute_swan/England/234135/2020(H5N8) | 2020-Dec-01 | EPI_ISL_1123360 | England | Mute swan | b2 | b2 (N8) | Eurasian HPAI H5 | Eurasian HPAI H5 | Eurasian HPAI H5 | Eurasian HPAI H5 | Eurasian HPAI H5 | Eurasian HPAI H5 |
| A/mute_swan/England/263814/2020(H5N8) | 2020-Nov-10 | EPI_ISL_1123359 | England | Mute swan | b2 | b2 (N8) | Eurasian HPAI H5 | Eurasian HPAI H5 | Eurasian HPAI H5 | Eurasian HPAI H5 | Eurasian HPAI H5 | Eurasian HPAI H5 |
| A/mute_swan/Wales/048068/2020(H5N5) | 2020-Nov-24 | EPI_ISL_683999 | England | Mute swan | b2 | N5 | Eurasian HPAI H5 | Null | LPAIV | Eurasian HPAI H5 | Eurasian HPAI H5 | Eurasian HPAI H5 |
| A/turkey/England/037784/2020(H5N8) | 2020-Nov-28 | EPI_ISL_710504 | England | Turkey | b2 | b2 (N8) | Eurasian HPAI H5 | Eurasian HPAI H5 | Eurasian HPAI H5 | Eurasian HPAI H5 | Eurasian HPAI H5 | Eurasian HPAI H5 |
| A/turkey/England/038115/2020(H5N8) | 2020-Dec-02 | EPI_ISL_710505 | England | Turkey | b2 | b2 (N8) | Eurasian HPAI H5 | Eurasian HPAI H5 | Eurasian HPAI H5 | Eurasian HPAI H5 | Eurasian HPAI H5 | Eurasian HPAI H5 |
| A/turkey/England/038730/2020(H5N8) | 2020-Dec-03 | EPI_ISL_766052 | England | Turkey | b2 | b2 (N8) | Eurasian HPAI H5 | Eurasian HPAI H5 | Eurasian HPAI H5 | Eurasian HPAI H5 | Eurasian HPAI H5 | Eurasian HPAI H5 |
| A/turkey/England/039352/2020(H5N8) | 2020-Dec-04 | EPI_ISL_766053 | England | Turkey | b2 | b2 (N8) | Eurasian HPAI H5 | Eurasian HPAI H5 | Eurasian HPAI H5 | Eurasian HPAI H5 | Eurasian HPAI H5 | Eurasian HPAI H5 |
| A/turkey/England/039472/2020(H5N8) | 2020-Dec-04 | EPI_ISL_766054 | England | Turkey | b2 | b2 (N8) | Eurasian HPAI H5 | Eurasian HPAI H5 | Eurasian HPAI H5 | Eurasian HPAI H5 | Eurasian HPAI H5 | Eurasian HPAI H5 |
| A/whistling_duck/England/035643/2020(H5N8) | 2020-Nov-18 | EPI_ISL_710512 | England | Whistling duck | b2 | b2 (N8) | Eurasian HPAI H5 | Eurasian HPAI H5 | Eurasian HPAI H5 | Eurasian HPAI H5 | Eurasian HPAI H5 | Eurasian HPAI H5 |
| A/chicken/France/20P016448/2020(H5N8) | 2020-Nov-10 | EPI_ISL_667810 | France | Chicken | b2 | b2 (N8) | Eurasian HPAI H5 | Eurasian HPAI H5 | Eurasian HPAI H5 | Eurasian HPAI H5 | Eurasian HPAI H5 | Eurasian HPAI H5 |
| A/barnacle_goose/Germany-SH/AI02167/2020(H5N8) | 2020-Oct-30 | EPI_ISL_614400 | Germany | Barnacle goose | b2 | b2 (N8) | Eurasian HPAI H5 | Eurasian HPAI H5 | Eurasian HPAI H5 | Eurasian HPAI H5 | Eurasian HPAI H5 | Eurasian HPAI H5 |
| A/buzzard/Germany-MV/AI02166/2020(H5N5) | 2020-Oct-29 | EPI_ISL_614399 | Germany | Buzzard | b2 | N5 | Eurasian HPAI H5 | Eurasian HPAI H5 | LPAIV | Eurasian HPAI H5 | Eurasian HPAI H5 | Eurasian HPAI H5 |
| A/red_knot/Germany-SH/AI03419/2020(H5N3) | 2020-Dec-14 | EPI_ISL_1205485 | Germany | Red knot | b2 | N3 | LPAIV | LPAIV | LPAIV | LPAIV | Eurasian HPAI H5 | LPAIV |
| A/red_knot/Germany-SH/AI03421/2020(H5N3) | 2020-Dec-14 | EPI_ISL_1205487 | Germany | Red knot | b2 | N3 | LPAIV | LPAIV | LPAIV | LPAIV | Eurasian HPAI H5 | LPAIV |
| A/red_knot/Germany-SH/AI03424/2020(H5N3) | 2020-Dec-16 | EPI_ISL_1205489 | Germany | Red knot | b2 | N3 | LPAIV | LPAIV | LPAIV | LPAIV | Eurasian HPAI H5 | LPAIV |
| A/chicken/Iraq/1/2020(H5N8) | 2020-May-12 | EPI_ISL_623074 | Iraq | Chicken | b2 | b2 (N8) | Eurasian HPAI H5 | Eurasian HPAI H5 | Eurasian HPAI H5 | Eurasian HPAI H5 | Eurasian HPAI H5 | Eurasian HPAI H5 |
| A/peregrine_falcon/Ireland/20VIR7872-1/2020(H5N8) | 2020-Nov-01 | EPI_ISL_813979 | Ireland | Peregrine falcon | b2 | b2 (N8) | Eurasian HPAI H5 | Eurasian HPAI H5 | Eurasian HPAI H5 | Eurasian HPAI H5 | Eurasian HPAI H5 | Eurasian HPAI H5 |
| A/common_buzzard/Italy/21VIR431-7/2020(H5N8) | 2020-Nov-17 | EPI_ISL_1665272 | Italy | Common buzzard | b2 | b2 (N8) | Eurasian HPAI H5 | Eurasian HPAI H5 | Eurasian HPAI H5 | Null | Eurasian HPAI H5 | Eurasian HPAI H5 |
| A/common_teal/Italy/20VIR7439-190/2020(H5N5) | 2020-Nov-28 | EPI_ISL_956409 | Italy | Common teal | b2 | N5 | Eurasian HPAI H5 | Eurasian HPAI H5 | LPAIV | Eurasian HPAI H5 | Eurasian HPAI H5 | Eurasian HPAI H5 |
| A/common_teal/Italy/20VIR7439-191/2020(H5N8) | 2020-Nov-28 | EPI_ISL_1665251 | Italy | Common teal | b2 | b2 (N8) | Eurasian HPAI H5 | Eurasian HPAI H5 | Eurasian HPAI H5 | Null | Eurasian HPAI H5 | Eurasian HPAI H5 |
| A/common_teal/Italy/20VIR7608-73/2020(H5N8) | 2020-Dec-04 | EPI_ISL_956408 | Italy | Common teal | b2 | b2 (N8) | Eurasian HPAI H5 | Eurasian HPAI H5 | Null | Eurasian HPAI H5 | Eurasian HPAI H5 | Eurasian HPAI H5 |
| A/Eurasian_wigeon/Italy/20VIR7139-121/2020(H5N8) | 2020-Nov-14 | EPI_ISL_683593 | Italy | Eurasian wigeon | b2 | b2 (N8) | Eurasian HPAI H5 | Eurasian HPAI H5 | Eurasian HPAI H5 | Eurasian HPAI H5 | Eurasian HPAI H5 | Eurasian HPAI H5 |
| A/Eurasian_wigeon/Italy/20VIR7301-206/2020(H5N1) | 2020-Nov-21 | EPI_ISL_683592 | Italy | Eurasian wigeon | b2 | N1 | LPAIV | LPAIV | LPAIV | LPAIV | Eurasian HPAI H5 | LPAIV |
| A/Eurasian_wigeon/Italy/20VIR7301-31/2020(H5N8) | 2020-Nov-21 | EPI_ISL_683751 | Italy | Eurasian wigeon | b2 | b2 (N8) | Eurasian HPAI H5 | Eurasian HPAI H5 | Null | Eurasian HPAI H5 | Eurasian HPAI H5 | Eurasian HPAI H5 |
| A/Eurasian_wigeon/Italy/20VIR7301-34/2020(H5N8) | 2020-Nov-21 | EPI_ISL_683752 | Italy | Eurasian wigeon | b2 | b2 (N8) | Eurasian HPAI H5 | Eurasian HPAI H5 | Null | Eurasian HPAI H5 | Eurasian HPAI H5 | Eurasian HPAI H5 |
| A/Eurasian_wigeon/Italy/20VIR7301-362/2020(H5N8) | 2020-Nov-21 | EPI_ISL_956414 | Italy | Eurasian wigeon | b2 | b2 (N8) | Eurasian HPAI H5 | Eurasian HPAI H5 | Eurasian HPAI H5 | Eurasian HPAI H5 | Eurasian HPAI H5 | Eurasian HPAI H5 |
| A/greater_white-fronted_goose/Italy/20VIR8073-4/2020(H5N1) | 2020-Nov-23 | EPI_ISL_956412 | Italy | Greater white-fronted goose | b2 | N1 | LPAIV | LPAIV | LPAIV | LPAIV | Eurasian HPAI H5 | LPAIV |
| A/greylag_goose/Italy/20VIR7660-6/2020(H5N8) | 2020-Nov-29 | EPI_ISL_956411 | Italy | Greylag goose | b2 | b2 (N8) | Eurasian HPAI H5 | Eurasian HPAI H5 | Eurasian HPAI H5 | Eurasian HPAI H5 | Eurasian HPAI H5 | Eurasian HPAI H5 |
| A/mallard/Italy/20VIR7139-124_feather/2020(H5N8) | 2020-Nov-14 | EPI_ISL_683594 | Italy | Mallard | b2 | b2 (N8) | Eurasian HPAI H5 | Eurasian HPAI H5 | Eurasian HPAI H5 | Eurasian HPAI H5 | Eurasian HPAI H5 | Eurasian HPAI H5 |
| A/mallard/Italy/20VIR7139-73/2020(H5N8) | 2020-Nov-14 | EPI_ISL_654958 | Italy | Mallard | b2 | b2 (N8) | Eurasian HPAI H5 | Eurasian HPAI H5 | Eurasian HPAI H5 | Eurasian HPAI H5 | Eurasian HPAI H5 | Eurasian HPAI H5 |
| A/chicken/Kazakhstan/Kn-3/2020(H5N8) | 2020-Sep-18 | EPI_ISL_739686 | Kazakhstan | Chicken | b2 | b2 (N8) | Eurasian HPAI H5 | Eurasian HPAI H5 | Eurasian HPAI H5 | Eurasian HPAI H5 | Eurasian HPAI H5 | Eurasian HPAI H5 |
| A/chicken/Kazakhstan/Kn-6/2020(H5N8) | 2020-Sep-18 | EPI_ISL_739687 | Kazakhstan | Chicken | b2 | b2 (N8) | Eurasian HPAI H5 | Eurasian HPAI H5 | Eurasian HPAI H5 | Eurasian HPAI H5 | Eurasian HPAI H5 | Eurasian HPAI H5 |
| A/domestic_duck/Kazakhstan/1-274-20-B/2020(H5N8) | 2020-Sep-25 | EPI_ISL_615072 | Kazakhstan | Domestic duck | b2 | b2 (N8) | Eurasian HPAI H5 | Eurasian HPAI H5 | Eurasian HPAI H5 | Eurasian HPAI H5 | Eurasian HPAI H5 | Eurasian HPAI H5 |
| A/domestic_goose/Kazakhstan/1-242_2-20-B/2020(H5N8) | 2020-Sep-19 | EPI_ISL_615073 | Kazakhstan | Domestic goose | b2 | b2 (N8) | Eurasian HPAI H5 | Eurasian HPAI H5 | Null | Eurasian HPAI H5 | Eurasian HPAI H5 | Eurasian HPAI H5 |
| A/domestic_goose/Kazakhstan/1-248_2-20-B/2020(H5N8) | 2020-Sep-20 | EPI_ISL_615068 | Kazakhstan | Domestic goose | b2 | b2 (N8) | Null | Eurasian HPAI H5 | Eurasian HPAI H5 | Eurasian HPAI H5 | Eurasian HPAI H5 | Eurasian HPAI H5 |
| A/mute_swan/Kazakhstan/1-267-20-B/2020(H5N8) | 2020-Sep-23 | EPI_ISL_614401 | Kazakhstan | Mute swan | b2 | b2 (N8) | Eurasian HPAI H5 | Eurasian HPAI H5 | Eurasian HPAI H5 | Eurasian HPAI H5 | Eurasian HPAI H5 | Eurasian HPAI H5 |
| A/chicken/Korea/H440/2020(H5N8) | 2020-Dec-12 | EPI_ISL_985191 | Korea | Chicken | b2 | b2 (N8) | Eurasian HPAI H5 | Eurasian HPAI H5 | Eurasian HPAI H5 | Eurasian HPAI H5 | Eurasian HPAI H5 | Eurasian HPAI H5 |
| A/chicken/Korea/H510/2020(H5N8) | 2020-Dec-22 | EPI_ISL_985199 | Korea | Chicken | b2 | b2 (N8) | Eurasian HPAI H5 | Eurasian HPAI H5 | Eurasian HPAI H5 | Eurasian HPAI H5 | Eurasian HPAI H5 | Eurasian HPAI H5 |
| A/chicken/Korea/H525/2020(H5N8) | 2020-Dec-25 | EPI_ISL_985206 | Korea | Chicken | b2 | b2 (N8) | Eurasian HPAI H5 | Eurasian HPAI H5 | Eurasian HPAI H5 | Eurasian HPAI H5 | Eurasian HPAI H5 | Eurasian HPAI H5 |
| A/chicken/Korea/H526/2020(H5N8) | 2020-Dec-25 | EPI_ISL_985207 | Korea | Chicken | b2 | b2 (N8) | Eurasian HPAI H5 | Eurasian HPAI H5 | Eurasian HPAI H5 | Eurasian HPAI H5 | Eurasian HPAI H5 | Eurasian HPAI H5 |
| A/chicken/Korea/H544/2020(H5N8) | 2020-Dec-29 | EPI_ISL_1009682 | Korea | Chicken | b2 | b2 (N8) | Eurasian HPAI H5 | Eurasian HPAI H5 | Eurasian HPAI H5 | Eurasian HPAI H5 | Eurasian HPAI H5 | Eurasian HPAI H5 |
| A/chicken/Korea/H550/2020(H5N8) | 2020-Dec-31 | EPI_ISL_1009686 | Korea | Chicken | b2 | b2 (N8) | Eurasian HPAI H5 | Eurasian HPAI H5 | Eurasian HPAI H5 | Eurasian HPAI H5 | Eurasian HPAI H5 | Eurasian HPAI H5 |
| A/duck/Korea/H411/2020(H5N8) | 2020-Dec-08 | EPI_ISL_985184 | Korea | Duck | b2 | b2 (N8) | Eurasian HPAI H5 | Eurasian HPAI H5 | Eurasian HPAI H5 | Eurasian HPAI H5 | Eurasian HPAI H5 | Eurasian HPAI H5 |
| A/duck/Korea/H431/2020(H5N8) | 2020-Dec-10 | EPI_ISL_985187 | Korea | Duck | b2 | b2 (N8) | Eurasian HPAI H5 | Eurasian HPAI H5 | Eurasian HPAI H5 | Eurasian HPAI H5 | Eurasian HPAI H5 | Eurasian HPAI H5 |
| A/duck/Korea/H471/2020(H5N8) | 2020-Dec-16 | EPI_ISL_985196 | Korea | Duck | b2 | b2 (N8) | Eurasian HPAI H5 | Eurasian HPAI H5 | Eurasian HPAI H5 | Eurasian HPAI H5 | Eurasian HPAI H5 | Eurasian HPAI H5 |
| A/duck/Korea/H499/2020(H5N8) | 2020-Dec-20 | EPI_ISL_985197 | Korea | Duck | b2 | b2 (N8) | Eurasian HPAI H5 | Eurasian HPAI H5 | Eurasian HPAI H5 | Eurasian HPAI H5 | Eurasian HPAI H5 | Eurasian HPAI H5 |
| A/duck/Korea/H524/2020(H5N8) | 2020-Dec-24 | EPI_ISL_985205 | Korea | Duck | b2 | b2 (N8) | Eurasian HPAI H5 | Eurasian HPAI H5 | Eurasian HPAI H5 | Eurasian HPAI H5 | Eurasian HPAI H5 | Eurasian HPAI H5 |
| A/duck/Korea/H538/2020(H5N8) | 2020-Dec-28 | EPI_ISL_985211 | Korea | Duck | b2 | b2 (N8) | Eurasian HPAI H5 | Eurasian HPAI H5 | Eurasian HPAI H5 | Eurasian HPAI H5 | Eurasian HPAI H5 | Eurasian HPAI H5 |
| A/duck/Korea/H542/2020(H5N8) | 2020-Dec-29 | EPI_ISL_1009683 | Korea | Duck | b2 | b2 (N8) | Eurasian HPAI H5 | Eurasian HPAI H5 | Eurasian HPAI H5 | Eurasian HPAI H5 | Eurasian HPAI H5 | Eurasian HPAI H5 |
| A/duck/Korea/H549/2020(H5N8) | 2020-Dec-30 | EPI_ISL_1009685 | Korea | Duck | b2 | b2 (N8) | Eurasian HPAI H5 | Eurasian HPAI H5 | Eurasian HPAI H5 | Eurasian HPAI H5 | Eurasian HPAI H5 | Eurasian HPAI H5 |
| A/goose/Korea/H449/2020(H5N8) | 2020-Dec-14 | EPI_ISL_985194 | Korea | Goose | b2 | b2 (N8) | Eurasian HPAI H5 | Eurasian HPAI H5 | Eurasian HPAI H5 | Eurasian HPAI H5 | Eurasian HPAI H5 | Eurasian HPAI H5 |
| A/mallard/Korea/WA820/2020(H5N8) | 2020-Nov-25 | EPI_ISL_1009698 | Korea | Mallard | b2 | b2 (N8) | Eurasian HPAI H5 | Eurasian HPAI H5 | Eurasian HPAI H5 | Eurasian HPAI H5 | Eurasian HPAI H5 | Eurasian HPAI H5 |
| A/quail/Korea/H551/2020(H5N8) | 2020-Dec-31 | EPI_ISL_1009687 | Korea | Quail | b2 | b2 (N8) | Eurasian HPAI H5 | Eurasian HPAI H5 | Eurasian HPAI H5 | Eurasian HPAI H5 | Eurasian HPAI H5 | Eurasian HPAI H5 |
| A/spot-billed_duck/Korea/WA1000/2020(H5N8) | 2020-Dec-15 | EPI_ISL_1009708 | Korea | Spot-billed duck | b2 | b2 (N8) | Eurasian HPAI H5 | Eurasian HPAI H5 | Eurasian HPAI H5 | Eurasian HPAI H5 | Eurasian HPAI H5 | Eurasian HPAI H5 |
| A/White_peacock/Korea/H533/2020(H5N8) | 2020-Dec-28 | EPI_ISL_1009680 | Korea | White peacock | b2 | b2 (N8) | Eurasian HPAI H5 | Eurasian HPAI H5 | Eurasian HPAI H5 | Eurasian HPAI H5 | Eurasian HPAI H5 | Eurasian HPAI H5 |
| A/wild_bird/Korea/H467/2020(H5N8) | 2020-Dec-14 | EPI_ISL_1009707 | Korea | Wild bird | b2 | b2 (N8) | Eurasian HPAI H5 | Eurasian HPAI H5 | Eurasian HPAI H5 | Eurasian HPAI H5 | Eurasian HPAI H5 | Eurasian HPAI H5 |
| A/wild_bird/Korea/H496-3/2020(H5N8) | 2020-Dec-16 | EPI_ISL_1009709 | Korea | Wild bird | b2 | b2 (N8) | Eurasian HPAI H5 | Eurasian HPAI H5 | Eurasian HPAI H5 | Eurasian HPAI H5 | Eurasian HPAI H5 | Eurasian HPAI H5 |
| A/wild_duck/Korea/H331/2020(H5N8) | 2020-Nov-17 | EPI_ISL_1009710 | Korea | Wild bird | b2 | b2 (N8) | Eurasian HPAI H5 | Eurasian HPAI H5 | Eurasian HPAI H5 | Eurasian HPAI H5 | Eurasian HPAI H5 | Eurasian HPAI H5 |
| A/barnacle_goose/Netherlands/20016935-002/2020(H5N8) | 2020-Nov-01 | EPI_ISL_632317 | Netherlands | Barnacle goose | b2 | b2 (N8) | Eurasian HPAI H5 | Eurasian HPAI H5 | Eurasian HPAI H5 | Eurasian HPAI H5 | Eurasian HPAI H5 | Eurasian HPAI H5 |
| A/chicken/Netherlands/20016597-026030/2020(H5N8) | 2020-Oct-28 | EPI_ISL_603132 | Netherlands | Chicken | b2 | b2 (N8) | Eurasian HPAI H5 | Eurasian HPAI H5 | Eurasian HPAI H5 | Eurasian HPAI H5 | Eurasian HPAI H5 | Eurasian HPAI H5 |
| A/chicken/Netherlands/20016978-001/2020(H5N8) | 2020-Nov-02 | EPI_ISL_641377 | Netherlands | Chicken | b2 | b2 (N8) | Eurasian HPAI H5 | Eurasian HPAI H5 | Eurasian HPAI H5 | Eurasian HPAI H5 | Eurasian HPAI H5 | Eurasian HPAI H5 |
| A/chicken/Netherlands/20017639-001/2020(H5N8) | 2020-Nov-10 | EPI_ISL_641394 | Netherlands | Chicken | b2 | b2 (N8) | Eurasian HPAI H5 | Eurasian HPAI H5 | Eurasian HPAI H5 | Eurasian HPAI H5 | Eurasian HPAI H5 | Eurasian HPAI H5 |
| A/chicken/Netherlands/20017694-004/2020(H5N8) | 2020-Nov-11 | EPI_ISL_641395 | Netherlands | Chicken | b2 | b2 (N8) | Eurasian HPAI H5 | Eurasian HPAI H5 | Eurasian HPAI H5 | Eurasian HPAI H5 | Eurasian HPAI H5 | Eurasian HPAI H5 |
| A/chicken/Netherlands/20019879-001005/2020(H5N1) | 2020-Dec-14 | EPI_ISL_711055 | Netherlands | Chicken | b2 | N1 | LPAIV | LPAIV | LPAIV | LPAIV | Eurasian HPAI H5 | LPAIV |
| A/Common_Buzzard/Netherlands/4/2020(H5N8) | 2020-Nov-06 | EPI_ISL_1575129 | Netherlands | Common buzzard | b2 | b2 (N8) | Eurasian HPAI H5 | Eurasian HPAI H5 | Eurasian HPAI H5 | Eurasian HPAI H5 | Eurasian HPAI H5 | Eurasian HPAI H5 |
| A/eurasian_teal/Netherlands/20016896-013/2020(H5N1) | 2020-Nov-02 | EPI_ISL_632315 | Netherlands | Eurasian teal | b2 | N1 | LPAIV | LPAIV | LPAIV | LPAIV | Eurasian HPAI H5 | LPAIV |
| A/Eurasian_Wigeon/Netherlands/1/2020(H5N1) | 2020-Oct-16 | EPI_ISL_603133 | Netherlands | Eurasian wigeon | b2 | N1 | LPAIV | LPAIV | LPAIV | LPAIV | Eurasian HPAI H5 | LPAIV |
| A/Eurasian_Wigeon/Netherlands/4/2020(H5N1) | 2020-Oct-16 | EPI_ISL_603134 | Netherlands | Eurasian wigeon | b2 | N1 | LPAIV | LPAIV | LPAIV | LPAIV | Eurasian HPAI H5 | LPAIV |
| A/Eurasian_Wigeon/Netherlands/5/2020(H5N1) | 2020-Oct-16 | EPI_ISL_603135 | Netherlands | Eurasian wigeon | b2 | N1 | LPAIV | LPAIV | LPAIV | LPAIV | Eurasian HPAI H5 | LPAIV |
| A/Eurasian_Wigeon/Netherlands/7/2020(H5N8) | 2020-Oct-16 | EPI_ISL_603136 | Netherlands | Eurasian wigeon | b2 | b2 (N8) | Eurasian HPAI H5 | Eurasian HPAI H5 | Eurasian HPAI H5 | Eurasian HPAI H5 | Eurasian HPAI H5 | Eurasian HPAI H5 |
| A/Greylag_Goose/Netherlands/1/2020(H5N8) | 2020-Nov-06 | EPI_ISL_1575135 | Netherlands | Greylag goose | b2 | b2 (N8) | Eurasian HPAI H5 | Eurasian HPAI H5 | Eurasian HPAI H5 | Eurasian HPAI H5 | Eurasian HPAI H5 | Eurasian HPAI H5 |
| A/greylag_goose/Netherlands/20016582-004/2020(H5N1) | 2020-Oct-28 | EPI_ISL_632314 | Netherlands | Greylag goose | b2 | N1 | LPAIV | LPAIV | LPAIV | LPAIV | Eurasian HPAI H5 | LPAIV |
| A/greylag_goose/Netherlands/20016879-001/2020(H5N8) | 2020-Nov-01 | EPI_ISL_632318 | Netherlands | Greylag goose | b2 | b2 (N8) | Eurasian HPAI H5 | Eurasian HPAI H5 | Eurasian HPAI H5 | Eurasian HPAI H5 | Eurasian HPAI H5 | Eurasian HPAI H5 |
| A/Herring_Gull/Netherlands/1/2020(H5N8) | 2020-Nov-06 | EPI_ISL_1575131 | Netherlands | Herring gull | b2 | b2 (N8) | Eurasian HPAI H5 | Eurasian HPAI H5 | Eurasian HPAI H5 | Eurasian HPAI H5 | Eurasian HPAI H5 | Eurasian HPAI H5 |
| A/Herring_Gull/Netherlands/2/2020(H5N8) | 2020-Nov-06 | EPI_ISL_1575132 | Netherlands | Herring gull | b2 | b2 (N8) | Eurasian HPAI H5 | Eurasian HPAI H5 | Eurasian HPAI H5 | Eurasian HPAI H5 | Eurasian HPAI H5 | Eurasian HPAI H5 |
| A/Herring_Gull/Netherlands/3/2020(H5N8) | 2020-Nov-06 | EPI_ISL_1575133 | Netherlands | Herring gull | b2 | b2 (N8) | Eurasian HPAI H5 | Eurasian HPAI H5 | Eurasian HPAI H5 | Eurasian HPAI H5 | Eurasian HPAI H5 | Eurasian HPAI H5 |
| A/Mute_Swan/Netherlands/1/2020(H5N8) | 2020-Nov-06 | EPI_ISL_1575130 | Netherlands | Mute swan | b2 | b2 (N8) | Eurasian HPAI H5 | Eurasian HPAI H5 | Eurasian HPAI H5 | Eurasian HPAI H5 | Eurasian HPAI H5 | Eurasian HPAI H5 |
| A/mute_swan/Netherlands/20015931-001/2020(H5N8) | 2020-Oct-17 | EPI_ISL_591075 | Netherlands | Mute swan | b2 | b2 (N8) | Eurasian HPAI H5 | Eurasian HPAI H5 | Eurasian HPAI H5 | Eurasian HPAI H5 | Eurasian HPAI H5 | Eurasian HPAI H5 |
| A/Mute_Swan/Netherlands/3/2020(H5N8) | 2020-Nov-06 | EPI_ISL_1575134 | Netherlands | Mute swan | b2 | b2 (N8) | Eurasian HPAI H5 | Eurasian HPAI H5 | Eurasian HPAI H5 | Eurasian HPAI H5 | Eurasian HPAI H5 | Eurasian HPAI H5 |
| A/Mute_Swan/Netherlands/5/2020(H5N8) | 2020-Nov-06 | EPI_ISL_1575136 | Netherlands | Mute swan | b2 | b2 (N8) | Eurasian HPAI H5 | Eurasian HPAI H5 | Eurasian HPAI H5 | Eurasian HPAI H5 | Eurasian HPAI H5 | Eurasian HPAI H5 |
| A/turkey/Norway/FU496/2020(H5N8) | 2020-Nov-30 | EPI_ISL_1295639 | Norway | Turkey | b2 | Null | Eurasian HPAI H5 | Eurasian HPAI H5 | Eurasian HPAI H5 | Eurasian HPAI H5 | Null | Eurasian HPAI H5 |
| A/chicken/Poland/448/2020(H5N8) | 2020-Nov-24 | EPI_ISL_661177 | Poland | Chicken | b2 | b2 (N8) | Eurasian HPAI H5 | Eurasian HPAI H5 | Eurasian HPAI H5 | Eurasian HPAI H5 | Eurasian HPAI H5 | Eurasian HPAI H5 |
| A/chicken/Poland/474/2020(H5N8) | 2020-Dec-03 | EPI_ISL_846601 | Poland | Chicken | b2 | b2 (N8) | Eurasian HPAI H5 | Eurasian HPAI H5 | Eurasian HPAI H5 | Eurasian HPAI H5 | Eurasian HPAI H5 | Eurasian HPAI H5 |
| A/chicken/Poland/565/2020(H5N8) | 2020-Dec-28 | EPI_ISL_846617 | Poland | Chicken | b2 | Null | Null | Null | Null | Null | Null | Null |
| A/swan/Poland/MB141/2020(H5N8) | 2020-Dec-16 | EPI_ISL_846623 | Poland | Swan | b2 | b2 (N8) | Eurasian HPAI H5 | Eurasian HPAI H5 | Eurasian HPAI H5 | Eurasian HPAI H5 | Eurasian HPAI H5 | Eurasian HPAI H5 |
| A/tundra_bean_goose/Poland/MB128/2020(H5N8) | 2020-Dec-08 | EPI_ISL_846625 | Poland | Tundra bean goose | b2 | Null | Null | Null | Null | Null | Null | Null |
| A/tundra_bean_goose/Poland/MB132/2020(H5N8) | 2020-Dec-09 | EPI_ISL_1220094 | Poland | Tundra bean goose | b2 | b2 (N8) | Eurasian HPAI H5 | Eurasian HPAI H5 | Eurasian HPAI H5 | Eurasian HPAI H5 | Eurasian HPAI H5 | Eurasian HPAI H5 |
| A/turkey/Poland/464/2020(H5N8) | 2020-Dec-01 | EPI_ISL_779129 | Poland | Turkey | b2 | b2 (N8) | Eurasian HPAI H5 | Eurasian HPAI H5 | Eurasian HPAI H5 | Eurasian HPAI H5 | Eurasian HPAI H5 | Eurasian HPAI H5 |
| A/turkey/Poland/475/2020(H5N8) | 2020-Dec-03 | EPI_ISL_846602 | Poland | Turkey | b2 | b2 (N8) | Eurasian HPAI H5 | Eurasian HPAI H5 | Eurasian HPAI H5 | Eurasian HPAI H5 | Eurasian HPAI H5 | Eurasian HPAI H5 |
| A/turkey/Poland/542/2020(H5N8) | 2020-Dec-21 | EPI_ISL_846614 | Poland | Turkey | b2 | Null | Null | Null | Null | Null | Null | Null |
| A/wild_goose/Poland/MB142/2020(H5N8) | 2020-Dec-15 | EPI_ISL_846624 | Poland | Wild goose | b2 | b2 (N8) | Eurasian HPAI H5 | Eurasian HPAI H5 | Eurasian HPAI H5 | Eurasian HPAI H5 | Eurasian HPAI H5 | Eurasian HPAI H5 |
| A/Astrakhan/3212/2020(H5N8) | 2020-Dec-12 | EPI_ISL_1038924 | Russia | Human | b2 | b2 (N8) | Eurasian HPAI H5 | Eurasian HPAI H5 | Eurasian HPAI H5 | Eurasian HPAI H5 | Eurasian HPAI H5 | Eurasian HPAI H5 |
| A/chicken/Astrakhan/2171-1/2020(H5N8) | 2020-Dec-07 | EPI_ISL_1185026 | Russia | Chicken | b2 | b2 (N8) | Eurasian HPAI H5 | Eurasian HPAI H5 | Eurasian HPAI H5 | Eurasian HPAI H5 | Eurasian HPAI H5 | Eurasian HPAI H5 |
| A/chicken/Astrakhan/321-01/2020(H5N8) | 2020-Dec-12 | EPI_ISL_1039232 | Russia | Chicken | b2 | b2 (N8) | Eurasian HPAI H5 | Eurasian HPAI H5 | Eurasian HPAI H5 | Eurasian HPAI H5 | Eurasian HPAI H5 | Eurasian HPAI H5 |
| A/chicken/Astrakhan/321-05/2020(H5N8) | 2020-Dec-12 | EPI_ISL_1039235 | Russia | Chicken | b2 | b2 (N8) | Eurasian HPAI H5 | Eurasian HPAI H5 | Eurasian HPAI H5 | Eurasian HPAI H5 | Eurasian HPAI H5 | Eurasian HPAI H5 |
| A/chicken/Astrakhan/321-06/2020(H5N8) | 2020-Dec-12 | EPI_ISL_1039236 | Russia | Chicken | b2 | b2 (N8) | Eurasian HPAI H5 | Eurasian HPAI H5 | Eurasian HPAI H5 | Eurasian HPAI H5 | Eurasian HPAI H5 | Eurasian HPAI H5 |
| A/chicken/Astrakhan/321-09/2020(H5N8) | 2020-Dec-12 | EPI_ISL_1039238 | Russia | Chicken | b2 | b2 (N8) | Eurasian HPAI H5 | Eurasian HPAI H5 | Eurasian HPAI H5 | Eurasian HPAI H5 | Eurasian HPAI H5 | Eurasian HPAI H5 |
| A/chicken/Astrakhan/321-10/2020(H5N8) | 2020-Dec-12 | EPI_ISL_1039240 | Russia | Chicken | b2 | b2 (N8) | Eurasian HPAI H5 | Eurasian HPAI H5 | Eurasian HPAI H5 | Eurasian HPAI H5 | Eurasian HPAI H5 | Eurasian HPAI H5 |
| A/chicken/Chelyabinsk/201/2020(H5N8) | 2020-Sep-08 | EPI_ISL_654833 | Russia | Chicken | b2 | b2 (N8) | Eurasian HPAI H5 | Eurasian HPAI H5 | Eurasian HPAI H5 | Eurasian HPAI H5 | Eurasian HPAI H5 | Eurasian HPAI H5 |
| A/chicken/Chelyabinsk/401/2020(H5N8) | 2020-Aug-06 | EPI_ISL_644145 | Russia | Chicken | b2 | b2 (N8) | Eurasian HPAI H5 | Eurasian HPAI H5 | Eurasian HPAI H5 | Eurasian HPAI H5 | Eurasian HPAI H5 | Eurasian HPAI H5 |
| A/chicken/Chelyabinsk/402/2020(H5N8) | 2020-Aug-06 | EPI_ISL_644146 | Russia | Chicken | b2 | b2 (N8) | Eurasian HPAI H5 | Eurasian HPAI H5 | Eurasian HPAI H5 | Eurasian HPAI H5 | Eurasian HPAI H5 | Eurasian HPAI H5 |
| A/chicken/Chelyabinsk/403/2020(H5N8) | 2020-Aug-06 | EPI_ISL_644160 | Russia | Chicken | b2 | b2 (N8) | Eurasian HPAI H5 | Eurasian HPAI H5 | Eurasian HPAI H5 | Eurasian HPAI H5 | Eurasian HPAI H5 | Eurasian HPAI H5 |
| A/chicken/Chelyabinsk/404/2020(H5N8) | 2020-Aug-06 | EPI_ISL_644148 | Russia | Chicken | b2 | b2 (N8) | Eurasian HPAI H5 | Eurasian HPAI H5 | Eurasian HPAI H5 | Eurasian HPAI H5 | Eurasian HPAI H5 | Eurasian HPAI H5 |
| A/chicken/Kostroma/304-01/2020(H5N8) | 2020-Oct-17 | EPI_ISL_1114737 | Russia | Chicken | b2 | b2 (N8) | Eurasian HPAI H5 | Eurasian HPAI H5 | Eurasian HPAI H5 | Eurasian HPAI H5 | Eurasian HPAI H5 | Eurasian HPAI H5 |
| A/chicken/Kostroma/304-03/2020(H5N8) | 2020-Oct-17 | EPI_ISL_1114738 | Russia | Chicken | b2 | b2 (N8) | Eurasian HPAI H5 | Eurasian HPAI H5 | Eurasian HPAI H5 | Eurasian HPAI H5 | Eurasian HPAI H5 | Eurasian HPAI H5 |
| A/chicken/Kostroma/304-04/2020(H5N8) | 2020-Oct-17 | EPI_ISL_1114740 | Russia | Chicken | b2 | b2 (N8) | Eurasian HPAI H5 | Eurasian HPAI H5 | Eurasian HPAI H5 | Eurasian HPAI H5 | Eurasian HPAI H5 | Eurasian HPAI H5 |
| A/chicken/Kostroma/304-06/2020(H5N8) | 2020-Oct-17 | EPI_ISL_1114741 | Russia | Chicken | b2 | b2 (N8) | Eurasian HPAI H5 | Eurasian HPAI H5 | Eurasian HPAI H5 | Eurasian HPAI H5 | Eurasian HPAI H5 | Eurasian HPAI H5 |
| A/chicken/Kostroma/304-08/2020(H5N8) | 2020-Oct-17 | EPI_ISL_1114742 | Russia | Chicken | b2 | b2 (N8) | Eurasian HPAI H5 | Eurasian HPAI H5 | Eurasian HPAI H5 | Eurasian HPAI H5 | Eurasian HPAI H5 | Eurasian HPAI H5 |
| A/chicken/Kostroma/304-10/2020(H5N8) | 2020-Oct-17 | EPI_ISL_1114745 | Russia | Chicken | b2 | b2 (N8) | Eurasian HPAI H5 | Eurasian HPAI H5 | Eurasian HPAI H5 | Eurasian HPAI H5 | Eurasian HPAI H5 | Eurasian HPAI H5 |
| A/chicken/Kurgan/1001/2020(H5N8) | 2020-Aug-27 | EPI_ISL_654834 | Russia | Chicken | b2 | b2 (N8) | Eurasian HPAI H5 | Eurasian HPAI H5 | Eurasian HPAI H5 | Eurasian HPAI H5 | Eurasian HPAI H5 | Eurasian HPAI H5 |
| A/chicken/Kurgan/1003/2020(H5N8) | 2020-Aug-27 | EPI_ISL_654835 | Russia | Chicken | b2 | b2 (N8) | Eurasian HPAI H5 | Eurasian HPAI H5 | Eurasian HPAI H5 | Eurasian HPAI H5 | Eurasian HPAI H5 | Eurasian HPAI H5 |
| A/chicken/Kurgan/1004/2020(H5N8) | 2020-Aug-27 | EPI_ISL_654836 | Russia | Chicken | b2 | b2 (N8) | Eurasian HPAI H5 | Eurasian HPAI H5 | Eurasian HPAI H5 | Eurasian HPAI H5 | Eurasian HPAI H5 | Eurasian HPAI H5 |
| A/chicken/Kurgan/1005/2020(H5N8) | 2020-Aug-27 | EPI_ISL_654837 | Russia | Chicken | b2 | b2 (N8) | Eurasian HPAI H5 | Eurasian HPAI H5 | Eurasian HPAI H5 | Eurasian HPAI H5 | Eurasian HPAI H5 | Eurasian HPAI H5 |
| A/chicken/Kurgan/1010/2020(H5N8) | 2020-Aug-27 | EPI_ISL_654838 | Russia | Chicken | b2 | b2 (N8) | Eurasian HPAI H5 | Eurasian HPAI H5 | Eurasian HPAI H5 | Eurasian HPAI H5 | Eurasian HPAI H5 | Eurasian HPAI H5 |
| A/chicken/Omsk/0073/2020(H5N8) | 2020-Aug-17 | EPI_ISL_644158 | Russia | Chicken | b2 | b2 (N8) | Eurasian HPAI H5 | Eurasian HPAI H5 | Eurasian HPAI H5 | Eurasian HPAI H5 | Eurasian HPAI H5 | Eurasian HPAI H5 |
| A/chicken/Omsk/0112/2020(H5N8) | 2020-Aug-17 | EPI_ISL_644150 | Russia | Chicken | b2 | b2 (N8) | Eurasian HPAI H5 | Eurasian HPAI H5 | Null | Eurasian HPAI H5 | Eurasian HPAI H5 | Eurasian HPAI H5 |
| A/chicken/Omsk/0118/2020(H5N8) | 2020-Aug-17 | EPI_ISL_644134 | Russia | Chicken | b2 | b2 (N8) | Eurasian HPAI H5 | Eurasian HPAI H5 | Eurasian HPAI H5 | Eurasian HPAI H5 | Eurasian HPAI H5 | Eurasian HPAI H5 |
| A/chicken/Omsk/0119/2020(H5N8) | 2020-Aug-17 | EPI_ISL_644155 | Russia | Chicken | b2 | b2 (N8) | Eurasian HPAI H5 | Eurasian HPAI H5 | Eurasian HPAI H5 | Eurasian HPAI H5 | Eurasian HPAI H5 | Eurasian HPAI H5 |
| A/chicken/Omsk/30007/2020(H5N8) | 2020-Sep-03 | EPI_ISL_654830 | Russia | Chicken | b2 | b2 (N8) | Eurasian HPAI H5 | Eurasian HPAI H5 | Eurasian HPAI H5 | Eurasian HPAI H5 | Eurasian HPAI H5 | Eurasian HPAI H5 |
| A/chicken/Rostov-on-Don/308-02/2020(H5N8) | 2020-Oct-25 | EPI_ISL_1114746 | Russia | Chicken | b2 | b2 (N8) | Eurasian HPAI H5 | Eurasian HPAI H5 | Eurasian HPAI H5 | Eurasian HPAI H5 | Eurasian HPAI H5 | Eurasian HPAI H5 |
| A/chicken/Rostov-on-Don/308-03/2020(H5N8) | 2020-Oct-25 | EPI_ISL_1114747 | Russia | Chicken | b2 | b2 (N8) | Eurasian HPAI H5 | Eurasian HPAI H5 | Eurasian HPAI H5 | Eurasian HPAI H5 | Eurasian HPAI H5 | Eurasian HPAI H5 |
| A/chicken/Rostov-on-Don/308-04/2020(H5N8) | 2020-Oct-25 | EPI_ISL_1114748 | Russia | Chicken | b2 | b2 (N8) | Eurasian HPAI H5 | Eurasian HPAI H5 | Eurasian HPAI H5 | Eurasian HPAI H5 | Eurasian HPAI H5 | Eurasian HPAI H5 |
| A/chicken/Russia_Novosibirsk_region/1910-1/2020(H5N8) | 2020-Sep-22 | EPI_ISL_739688 | Russia | Chicken | b2 | b2 (N8) | Eurasian HPAI H5 | Eurasian HPAI H5 | Eurasian HPAI H5 | Eurasian HPAI H5 | Eurasian HPAI H5 | Eurasian HPAI H5 |
| A/chicken/Russia_Novosibirsk_region/1910-2/2020(H5N8) | 2020-Sep-22 | EPI_ISL_739689 | Russia | Chicken | b2 | b2 (N8) | Eurasian HPAI H5 | Eurasian HPAI H5 | Eurasian HPAI H5 | Eurasian HPAI H5 | Eurasian HPAI H5 | Eurasian HPAI H5 |
| A/chicken/Russia_Novosibirsk_region/3-1/2020(H5N8) | 2020-Sep-20 | EPI_ISL_739690 | Russia | Chicken | b2 | LPAIV (N8) | LPAIV | LPAIV | LPAIV | LPAIV | Eurasian HPAI H5 | Eurasian HPAI H5 |
| A/chicken/Russia_Novosibirsk_region/3-15/2020(H5N8) | 2020-Sep-20 | EPI_ISL_739691 | Russia | Chicken | b2 | LPAIV (N8) | LPAIV | LPAIV | LPAIV | LPAIV | Eurasian HPAI H5 | Eurasian HPAI H5 |
| A/chicken/Russia_Novosibirsk_region/3-29/2020(H5N8) | 2020-Sep-20 | EPI_ISL_739692 | Russia | Chicken | b2 | LPAIV (N8) | LPAIV | LPAIV | LPAIV | LPAIV | Eurasian HPAI H5 | Eurasian HPAI H5 |
| A/chicken/Russian_Federation/Omsk/1680-10/2020(H5N5) | 2020-Oct-02 | EPI_ISL_626647 | Russia | Chicken | b2 | N5 | Eurasian HPAI H5 | Eurasian HPAI H5 | Eurasian HPAI H5 | Eurasian HPAI H5 | Eurasian HPAI H5 | Eurasian HPAI H5 |
| A/chicken/Saratov/29801/2020(H5N8) | 2020-Sep-15 | EPI_ISL_654821 | Russia | Chicken | b2 | b2 (N8) | Eurasian HPAI H5 | Eurasian HPAI H5 | Eurasian HPAI H5 | Eurasian HPAI H5 | Eurasian HPAI H5 | Eurasian HPAI H5 |
| A/chicken/Tyumen/302-01/2020(H5N8) | 2020-Sep-26 | EPI_ISL_1114735 | Russia | Chicken | b2 | b2 (N8) | Eurasian HPAI H5 | Eurasian HPAI H5 | Eurasian HPAI H5 | Eurasian HPAI H5 | Eurasian HPAI H5 | Eurasian HPAI H5 |
| A/chicken/Tyumen/302-02/2020(H5N8) | 2020-Sep-26 | EPI_ISL_1114736 | Russia | Chicken | b2 | b2 (N8) | Eurasian HPAI H5 | Eurasian HPAI H5 | Eurasian HPAI H5 | Eurasian HPAI H5 | Eurasian HPAI H5 | Eurasian HPAI H5 |
| A/duck/Chelyabinsk/1207-1/2020(H5N8) | 2020-Jul-31 | EPI_ISL_637098 | Russia | Duck | b2 | b2 (N8) | Eurasian HPAI H5 | Eurasian HPAI H5 | Eurasian HPAI H5 | Eurasian HPAI H5 | Eurasian HPAI H5 | Eurasian HPAI H5 |
| A/duck/Omsk/0004/2020(H5N8) | 2020-Aug-13 | EPI_ISL_644149 | Russia | Duck | b2 | b2 (N8) | Eurasian HPAI H5 | Eurasian HPAI H5 | Eurasian HPAI H5 | Eurasian HPAI H5 | Eurasian HPAI H5 | Eurasian HPAI H5 |
| A/duck/Omsk/0075/2020(H5N8) | 2020-Aug-17 | EPI_ISL_644142 | Russia | Duck | b2 | b2 (N8) | Eurasian HPAI H5 | Eurasian HPAI H5 | Eurasian HPAI H5 | Eurasian HPAI H5 | Eurasian HPAI H5 | Eurasian HPAI H5 |
| A/duck/Omsk/0076/2020(H5N8) | 2020-Aug-17 | EPI_ISL_644159 | Russia | Duck | b2 | b2 (N8) | Eurasian HPAI H5 | Eurasian HPAI H5 | Eurasian HPAI H5 | Eurasian HPAI H5 | Eurasian HPAI H5 | Eurasian HPAI H5 |
| A/duck/Omsk/0077/2020(H5N8) | 2020-Aug-17 | EPI_ISL_644144 | Russia | Duck | b2 | b2 (N8) | Eurasian HPAI H5 | Eurasian HPAI H5 | Eurasian HPAI H5 | Eurasian HPAI H5 | Eurasian HPAI H5 | Eurasian HPAI H5 |
| A/duck/Russian_Federation/Omsk/1328-2/2020(H5N8) | 2020-Aug-17 | EPI_ISL_626650 | Russia | Duck | b2 | b2 (N8) | Eurasian HPAI H5 | Eurasian HPAI H5 | Eurasian HPAI H5 | Eurasian HPAI H5 | Eurasian HPAI H5 | Eurasian HPAI H5 |
| A/duck/Russian_Federation/Saratov/1578-2/2020(H5N8) | 2020-Sep-18 | EPI_ISL_626649 | Russia | Duck | b2 | b2 (N8) | Eurasian HPAI H5 | Eurasian HPAI H5 | Eurasian HPAI H5 | Eurasian HPAI H5 | Eurasian HPAI H5 | Eurasian HPAI H5 |
| A/duck/Saratov/29804/2020(H5N8) | 2020-Sep-15 | EPI_ISL_654824 | Russia | Duck | b2 | b2 (N8) | Eurasian HPAI H5 | Eurasian HPAI H5 | Eurasian HPAI H5 | Eurasian HPAI H5 | Eurasian HPAI H5 | Eurasian HPAI H5 |
| A/goose/Kurgan/01/2020(H5N8) | 2020-Aug-27 | EPI_ISL_654839 | Russia | Goose | b2 | b2 (N8) | Eurasian HPAI H5 | Eurasian HPAI H5 | Eurasian HPAI H5 | Eurasian HPAI H5 | Eurasian HPAI H5 | Eurasian HPAI H5 |
| A/goose/Omsk/0002/2020(H5N8) | 2020-Aug-13 | EPI_ISL_644122 | Russia | Goose | b2 | b2 (N8) | Eurasian HPAI H5 | Eurasian HPAI H5 | Eurasian HPAI H5 | Eurasian HPAI H5 | Eurasian HPAI H5 | Eurasian HPAI H5 |
| A/goose/Omsk/0071/2020(H5N8) | 2020-Aug-17 | EPI_ISL_644139 | Russia | Goose | b2 | b2 (N8) | Eurasian HPAI H5 | Eurasian HPAI H5 | Eurasian HPAI H5 | Eurasian HPAI H5 | Eurasian HPAI H5 | Eurasian HPAI H5 |
| A/goose/Omsk/0074/2020(H5N8) | 2020-Aug-17 | EPI_ISL_644141 | Russia | Goose | b2 | b2 (N8) | Eurasian HPAI H5 | Eurasian HPAI H5 | Eurasian HPAI H5 | Eurasian HPAI H5 | Eurasian HPAI H5 | Eurasian HPAI H5 |
| A/goose/Omsk/0111/2020(H5N8) | 2020-Aug-17 | EPI_ISL_644136 | Russia | Goose | b2 | b2 (N8) | Eurasian HPAI H5 | Eurasian HPAI H5 | Null | Eurasian HPAI H5 | Eurasian HPAI H5 | Eurasian HPAI H5 |
| A/goose/Omsk/011101/2020(H5N8) | 2020-Aug-17 | EPI_ISL_644136 | Russia | Goose | b2 | b2 (N8) | Eurasian HPAI H5 | Eurasian HPAI H5 | Eurasian HPAI H5 | Eurasian HPAI H5 | Eurasian HPAI H5 | Eurasian HPAI H5 |
| A/goose/Omsk/0113/2020(H5N8) | 2020-Aug-17 | EPI_ISL_644127 | Russia | Goose | b2 | b2 (N8) | Eurasian HPAI H5 | Eurasian HPAI H5 | Eurasian HPAI H5 | Eurasian HPAI H5 | Eurasian HPAI H5 | Eurasian HPAI H5 |
| A/goose/Omsk/0114/2020(H5N8) | 2020-Aug-17 | EPI_ISL_644151 | Russia | Goose | b2 | b2 (N8) | Eurasian HPAI H5 | Eurasian HPAI H5 | Eurasian HPAI H5 | Eurasian HPAI H5 | Eurasian HPAI H5 | Eurasian HPAI H5 |
| A/goose/Omsk/0115/2020(H5N8) | 2020-Aug-17 | EPI_ISL_644129 | Russia | Goose | b2 | b2 (N8) | Eurasian HPAI H5 | Eurasian HPAI H5 | Null | Eurasian HPAI H5 | Eurasian HPAI H5 | Eurasian HPAI H5 |
| A/goose/Omsk/01161/2020(H5N8) | 2020-Aug-17 | EPI_ISL_644152 | Russia | Goose | b2 | b2 (N8) | Eurasian HPAI H5 | Eurasian HPAI H5 | Eurasian HPAI H5 | Eurasian HPAI H5 | Eurasian HPAI H5 | Eurasian HPAI H5 |
| A/goose/Omsk/01171/2020(H5N8) | 2020-Aug-17 | EPI_ISL_644132 | Russia | Goose | b2 | b2 (N8) | Eurasian HPAI H5 | Eurasian HPAI H5 | Eurasian HPAI H5 | Eurasian HPAI H5 | Eurasian HPAI H5 | Eurasian HPAI H5 |
| A/goose/Omsk/30001/2020(H5N8) | 2020-Sep-03 | EPI_ISL_654826 | Russia | Goose | b2 | b2 (N8) | Eurasian HPAI H5 | Eurasian HPAI H5 | Eurasian HPAI H5 | Eurasian HPAI H5 | Eurasian HPAI H5 | Eurasian HPAI H5 |
| A/goose/Omsk/30003/2020(H5N8) | 2020-Sep-03 | EPI_ISL_654827 | Russia | Goose | b2 | b2 (N8) | Eurasian HPAI H5 | Eurasian HPAI H5 | Eurasian HPAI H5 | Eurasian HPAI H5 | Eurasian HPAI H5 | Eurasian HPAI H5 |
| A/goose/Omsk/30004/2020(H5N8) | 2020-Sep-03 | EPI_ISL_654828 | Russia | Goose | b2 | b2 (N8) | Eurasian HPAI H5 | Eurasian HPAI H5 | Eurasian HPAI H5 | Eurasian HPAI H5 | Eurasian HPAI H5 | Eurasian HPAI H5 |
| A/goose/Omsk/30006/2020(H5N8) | 2020-Sep-03 | EPI_ISL_654829 | Russia | Goose | b2 | b2 (N8) | Eurasian HPAI H5 | Eurasian HPAI H5 | Eurasian HPAI H5 | Eurasian HPAI H5 | Eurasian HPAI H5 | Eurasian HPAI H5 |
| A/goose/Omsk/30009/2020(H5N8) | 2020-Sep-03 | EPI_ISL_654831 | Russia | Goose | b2 | b2 (N8) | Eurasian HPAI H5 | Eurasian HPAI H5 | Eurasian HPAI H5 | Eurasian HPAI H5 | Eurasian HPAI H5 | Eurasian HPAI H5 |
| A/goose/Omsk/30010/2020(H5N8) | 2020-Sep-03 | EPI_ISL_654832 | Russia | Goose | b2 | b2 (N8) | Eurasian HPAI H5 | Eurasian HPAI H5 | Eurasian HPAI H5 | Eurasian HPAI H5 | Eurasian HPAI H5 | Eurasian HPAI H5 |
| A/goose/Russia_Novosibirsk_region/1-12/2020(H5N8) | 2020-Sep-15 | EPI_ISL_739684 | Russia | Goose | b2 | b2 (N8) | Eurasian HPAI H5 | Eurasian HPAI H5 | Eurasian HPAI H5 | Eurasian HPAI H5 | Eurasian HPAI H5 | Eurasian HPAI H5 |
| A/goose/Russia_Omsk_region/55-1/2020(H5N8) | 2020-Aug-29 | EPI_ISL_739685 | Russia | Goose | b2 | b2 (N8) | Eurasian HPAI H5 | Eurasian HPAI H5 | Eurasian HPAI H5 | Eurasian HPAI H5 | Eurasian HPAI H5 | Eurasian HPAI H5 |
| A/goose/Russian_Federation/Kurgan/1345-25/2020(H5N8) | 2020-Aug-20 | EPI_ISL_626651 | Russia | Goose | b2 | b2 (N8) | Eurasian HPAI H5 | Eurasian HPAI H5 | Eurasian HPAI H5 | Eurasian HPAI H5 | Eurasian HPAI H5 | Eurasian HPAI H5 |
| A/goose/Russian_Federation/Omsk/1680-6/2020(H5N5) | 2020-Oct-02 | EPI_ISL_626648 | Russia | Goose | b2 | N5 | Eurasian HPAI H5 | Eurasian HPAI H5 | Eurasian HPAI H5 | Eurasian HPAI H5 | Eurasian HPAI H5 | Eurasian HPAI H5 |
| A/mute_swan/North_Ossetia-Alania/325-01/2020(H5N8) | 2020-Dec-31 | EPI_ISL_1114752 | Russia | Mute swan | b2 | b2 (N8) | Eurasian HPAI H5 | Eurasian HPAI H5 | Eurasian HPAI H5 | Eurasian HPAI H5 | Eurasian HPAI H5 | Eurasian HPAI H5 |
| A/mute_swan/North_Ossetia-Alania/325-02/2020(H5N8) | 2020-Dec-31 | EPI_ISL_1114753 | Russia | Mute swan | b2 | b2 (N8) | Eurasian HPAI H5 | Eurasian HPAI H5 | Eurasian HPAI H5 | Eurasian HPAI H5 | Eurasian HPAI H5 | Eurasian HPAI H5 |
| A/mute_swan/North_Ossetia-Alania/325-03/2020(H5N8) | 2020-Dec-31 | EPI_ISL_1114754 | Russia | Mute swan | b2 | b2 (N8) | Eurasian HPAI H5 | Eurasian HPAI H5 | Eurasian HPAI H5 | Eurasian HPAI H5 | Eurasian HPAI H5 | Eurasian HPAI H5 |
| A/swan/Tumen/1479-2/2020(H5N8) | 2020-Sep-10 | EPI_ISL_661178 | Russia | Swan | b2 | b2 (N8) | Eurasian HPAI H5 | Eurasian HPAI H5 | Eurasian HPAI H5 | Eurasian HPAI H5 | Eurasian HPAI H5 | Eurasian HPAI H5 |
| A/turkey/Omsk/0001/2020(H5N8) | 2020-Aug-13 | EPI_ISL_644121 | Russia | Turkey | b2 | b2 (N8) | Eurasian HPAI H5 | Eurasian HPAI H5 | Eurasian HPAI H5 | Eurasian HPAI H5 | Eurasian HPAI H5 | Eurasian HPAI H5 |
| A/turkey/Omsk/0003/2020(H5N8) | 2020-Aug-13 | EPI_ISL_644123 | Russia | Turkey | b2 | b2 (N8) | Eurasian HPAI H5 | Eurasian HPAI H5 | Eurasian HPAI H5 | Eurasian HPAI H5 | Eurasian HPAI H5 | Eurasian HPAI H5 |
| A/turkey/Stavropol/320-01/2020(H5N8) | 2020-Dec-11 | EPI_ISL_1114749 | Russia | Turkey | b2 | b2 (N8) | Eurasian HPAI H5 | Eurasian HPAI H5 | Eurasian HPAI H5 | Eurasian HPAI H5 | Eurasian HPAI H5 | Eurasian HPAI H5 |
| A/turkey/Stavropol/320-02/2020(H5N8) | 2020-Dec-11 | EPI_ISL_1114750 | Russia | Turkey | b2 | b2 (N8) | Eurasian HPAI H5 | Eurasian HPAI H5 | Eurasian HPAI H5 | Eurasian HPAI H5 | Eurasian HPAI H5 | Eurasian HPAI H5 |
| A/turkey/Stavropol/320-03/2020(H5N8) | 2020-Dec-11 | EPI_ISL_1114751 | Russia | Turkey | b2 | b2 (N8) | Eurasian HPAI H5 | Eurasian HPAI H5 | Eurasian HPAI H5 | Eurasian HPAI H5 | Eurasian HPAI H5 | Eurasian HPAI H5 |
| A/wild_duck/Omsk/01111/2020(H5N8) | 2020-Aug-17 | EPI_ISL_644138 | Russia | Wild bird | b2 | b2 (N8) | Eurasian HPAI H5 | Eurasian HPAI H5 | Eurasian HPAI H5 | Eurasian HPAI H5 | Eurasian HPAI H5 | Eurasian HPAI H5 |
| A/mute_swan/Slovenia/1639-20_21VIR959-1/2020(H5N8) | 2020-Nov-17 | EPI_ISL_1665263 | Slovenia | Mute swan | b2 | b2 (N8) | Eurasian HPAI H5 | Eurasian HPAI H5 | Eurasian HPAI H5 | Null | Eurasian HPAI H5 | Eurasian HPAI H5 |
| A/mute_swan/Slovenia/1756-20_21VIR959-3/2020(H5N8) | 2020-Dec-02 | EPI_ISL_1665264 | Slovenia | Mute swan | b2 | b2 (N8) | Eurasian HPAI H5 | Eurasian HPAI H5 | Eurasian HPAI H5 | Eurasian HPAI H5 | Eurasian HPAI H5 | Eurasian HPAI H5 |
| A/mute_swan/Slovenia/1799-20_21VIR959-2/2020(H5N8) | 2020-Dec-09 | EPI_ISL_1665265 | Slovenia | Mute swan | b2 | b2 (N8) | Eurasian HPAI H5 | Eurasian HPAI H5 | Eurasian HPAI H5 | Eurasian HPAI H5 | Eurasian HPAI H5 | Eurasian HPAI H5 |
| A/mute_swan/Slovenia/1820-20_21VIR959-4/2020(H5N8) | 2020-Dec-10 | EPI_ISL_1665266 | Slovenia | Mute swan | b2 | b2 (N8) | Eurasian HPAI H5 | Eurasian HPAI H5 | Eurasian HPAI H5 | Eurasian HPAI H5 | Eurasian HPAI H5 | Eurasian HPAI H5 |
| A/mute_swan/Slovenia/1914-20_21VIR959-5/2020(H5N5) | 2020-Dec-24 | EPI_ISL_1665267 | Slovenia | Mute swan | b2 | N5 | Eurasian HPAI H5 | Eurasian HPAI H5 | LPAIV | Null | Eurasian HPAI H5 | Eurasian HPAI H5 |
| A/barnacle_goose/Sweden/SVA201117SZ0468/KN003355/2020(H5N8) | 2020-Nov-12 | EPI_ISL_668457 | Sweden | Barnacle goose | b2 | b2 (N8) | Eurasian HPAI H5 | Eurasian HPAI H5 | Eurasian HPAI H5 | Eurasian HPAI H5 | Eurasian HPAI H5 | Eurasian HPAI H5 |
| A/Peregrine_falcon/Sweden/SVA201117SZ0467/20KN003345/2020(H5N8) | 2020-Nov-10 | EPI_ISL_668456 | Sweden | Peregrine falcon | b2 | b2 (N8) | Eurasian HPAI H5 | Eurasian HPAI H5 | Eurasian HPAI H5 | Eurasian HPAI H5 | Eurasian HPAI H5 | Eurasian HPAI H5 |
| A/Turkey/Sweden/SVA201114SZ0001/20KN303106/2020(H5N8) | 2020-Nov-13 | EPI_ISL_647969 | Sweden | Turkey | b2 | b2 (N8) | Eurasian HPAI H5 | Eurasian HPAI H5 | Eurasian HPAI H5 | Eurasian HPAI H5 | Eurasian HPAI H5 | Eurasian HPAI H5 |
| * b1, 2.3.4.4b1; b2, 2.3.4.4b2  # LPAIV, low pathogenic avian influenza virus; HPAI, highly pathogenic avian influenza. | | | | | | | | | | | | |

**Appendix Table 8**. Log marginal likelihoods calculated with path sampling and stepping-stone sampling.

| Clock model | Coalescent model | Path sampling | Stepping-stone sampling |
| --- | --- | --- | --- |
| Strict clock | Constant size | -6359.21 | -6382.35 |
|  | Exponential growth | -6339.30 | -6352.38 |
|  | Bayesian skyline | -6335.76 | -6350.50 |
| **Uncorrelated lognormal relaxed clock** | Constant size | -6349.34 | -6364.48 |
|  | Exponential growth | -6336.57 | -6348.65 |
|  | **Bayesian skyline** | **-6327.97** | **-6337.61** |
