# Supplementary figures and images for: Emergence, prevalence, and evolution of H5N8 avian influenza viruses in central China, 2020
Source: Emerg Microbes Infect. 2021 Dec 22;11(1):73–82. doi: 10.1080/22221751.2021.2011622 (PMC8725850; doi:10.1080/22221751.2021.2011622)

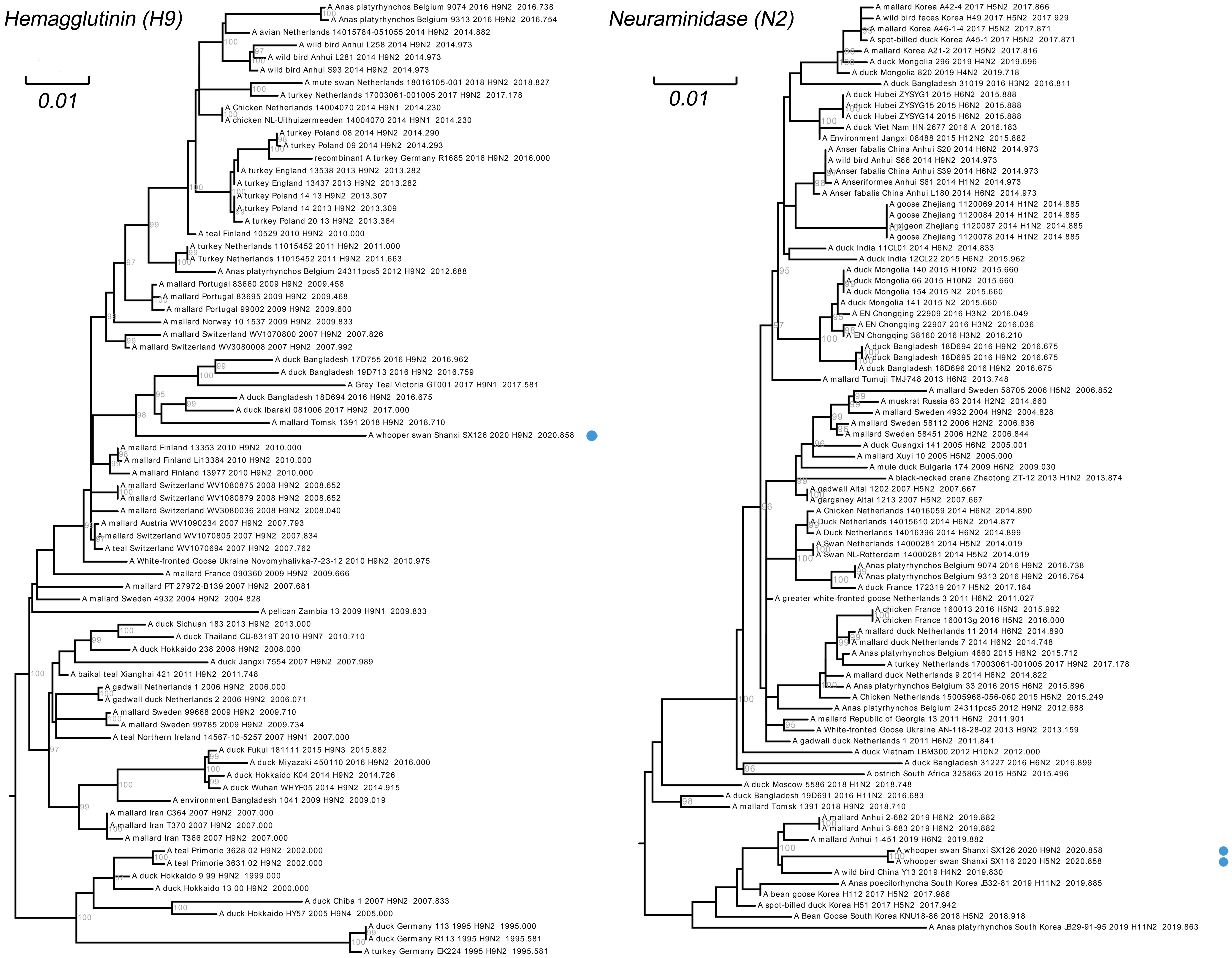

Supplement: Supplemental Material [file TEMI_A_2011622_SM3732.zip › Supplmentary files/Appendix Figure 1-H9N2-800.jpg]

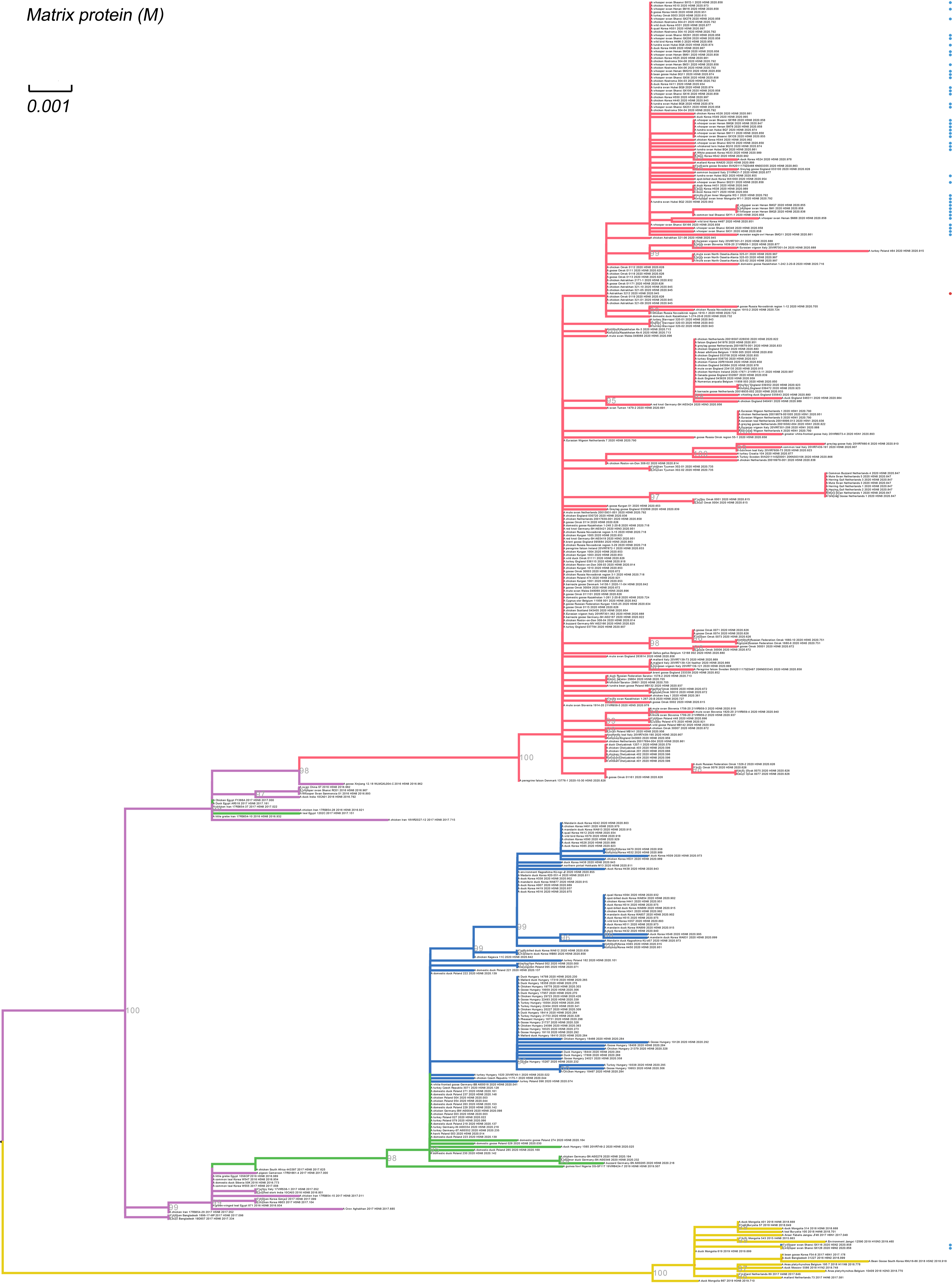

Supplement: Supplemental Material [file TEMI_A_2011622_SM3732.zip › Supplmentary files/Appendix Figure 1-M-1000.jpg]

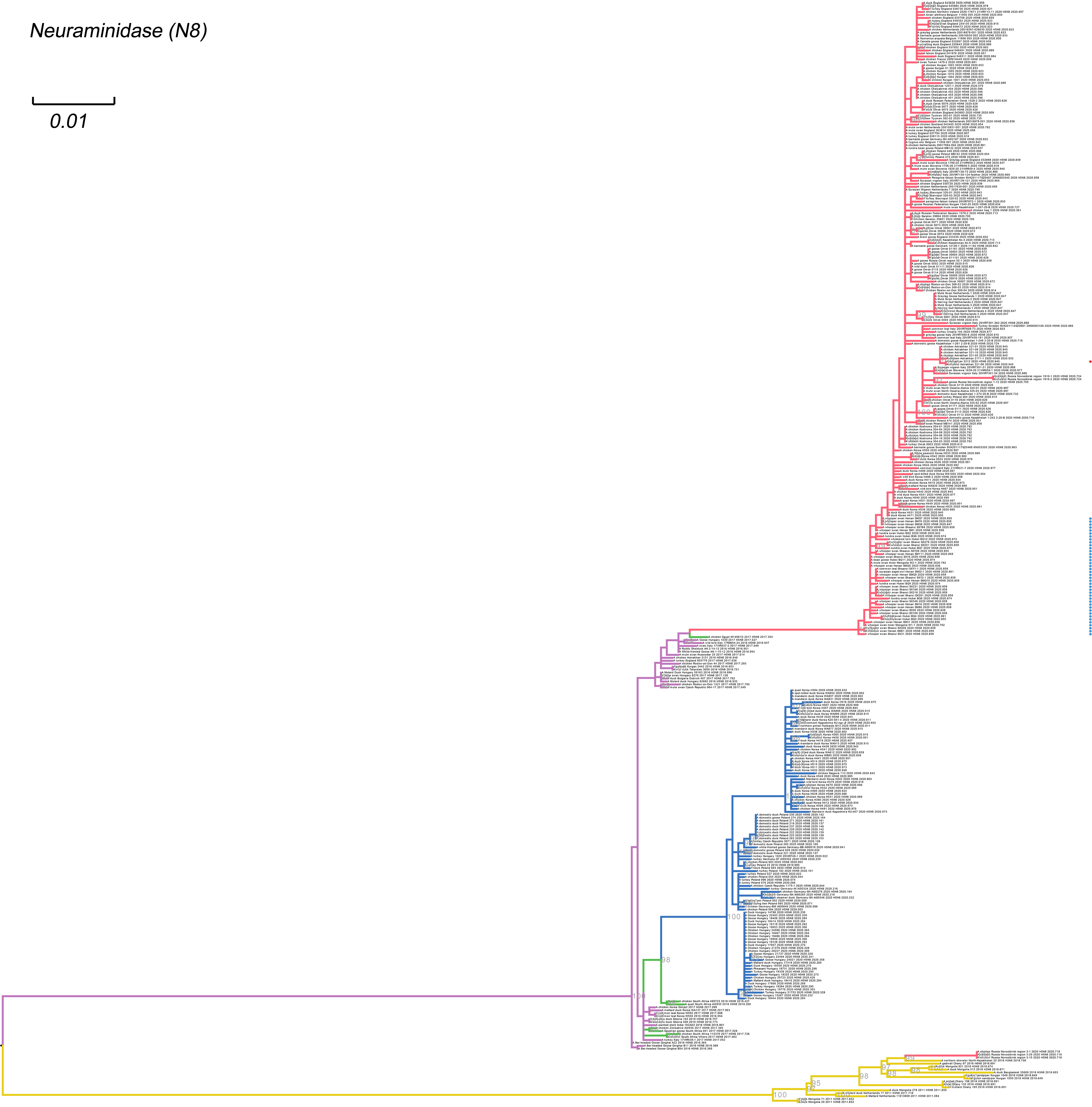

Supplement: Supplemental Material [file TEMI_A_2011622_SM3732.zip › Supplmentary files/Appendix Figure 1-N8-1000.jpg]

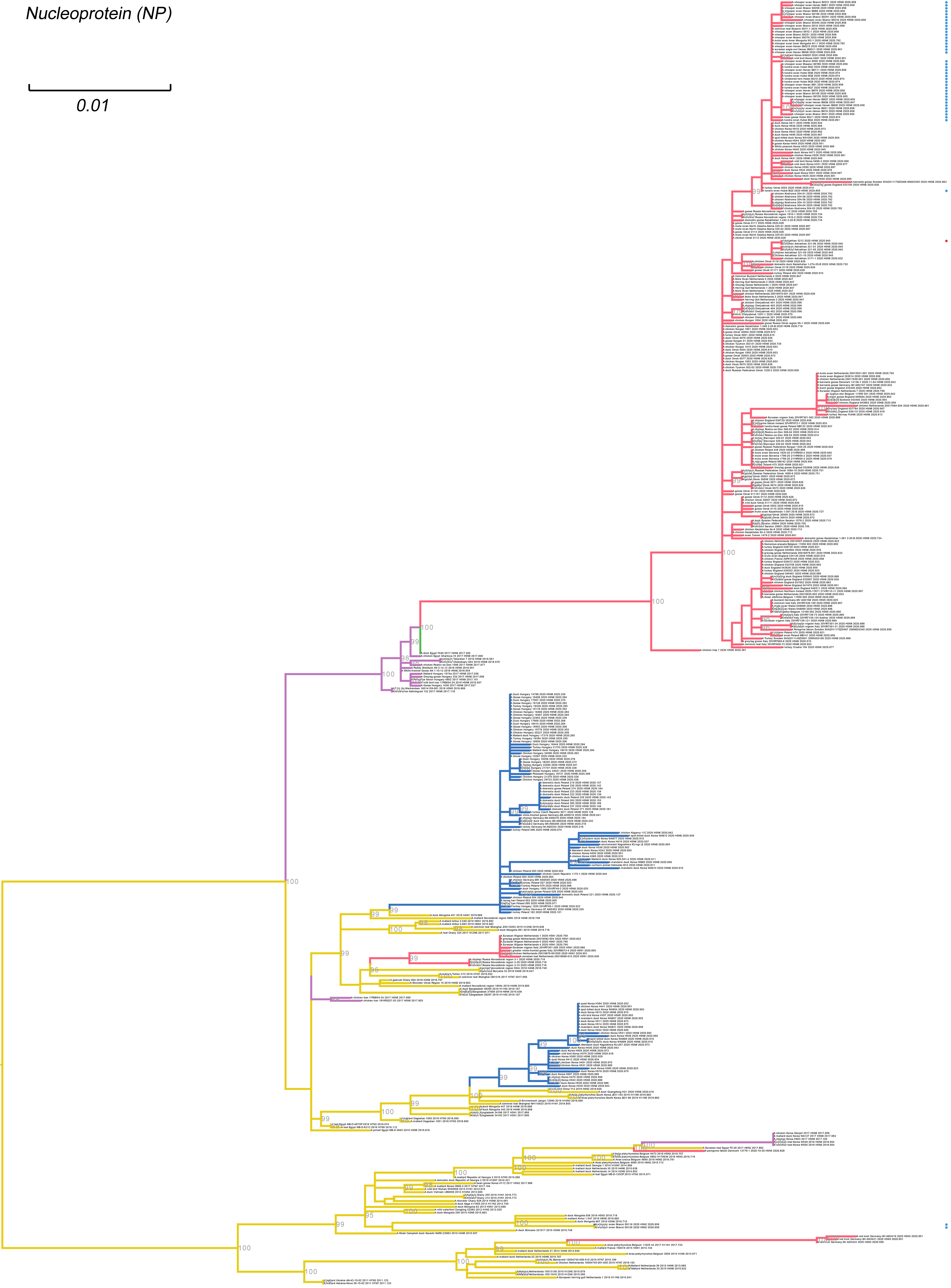

Supplement: Supplemental Material [file TEMI_A_2011622_SM3732.zip › Supplmentary files/Appendix Figure 1-NP-1000.jpg]

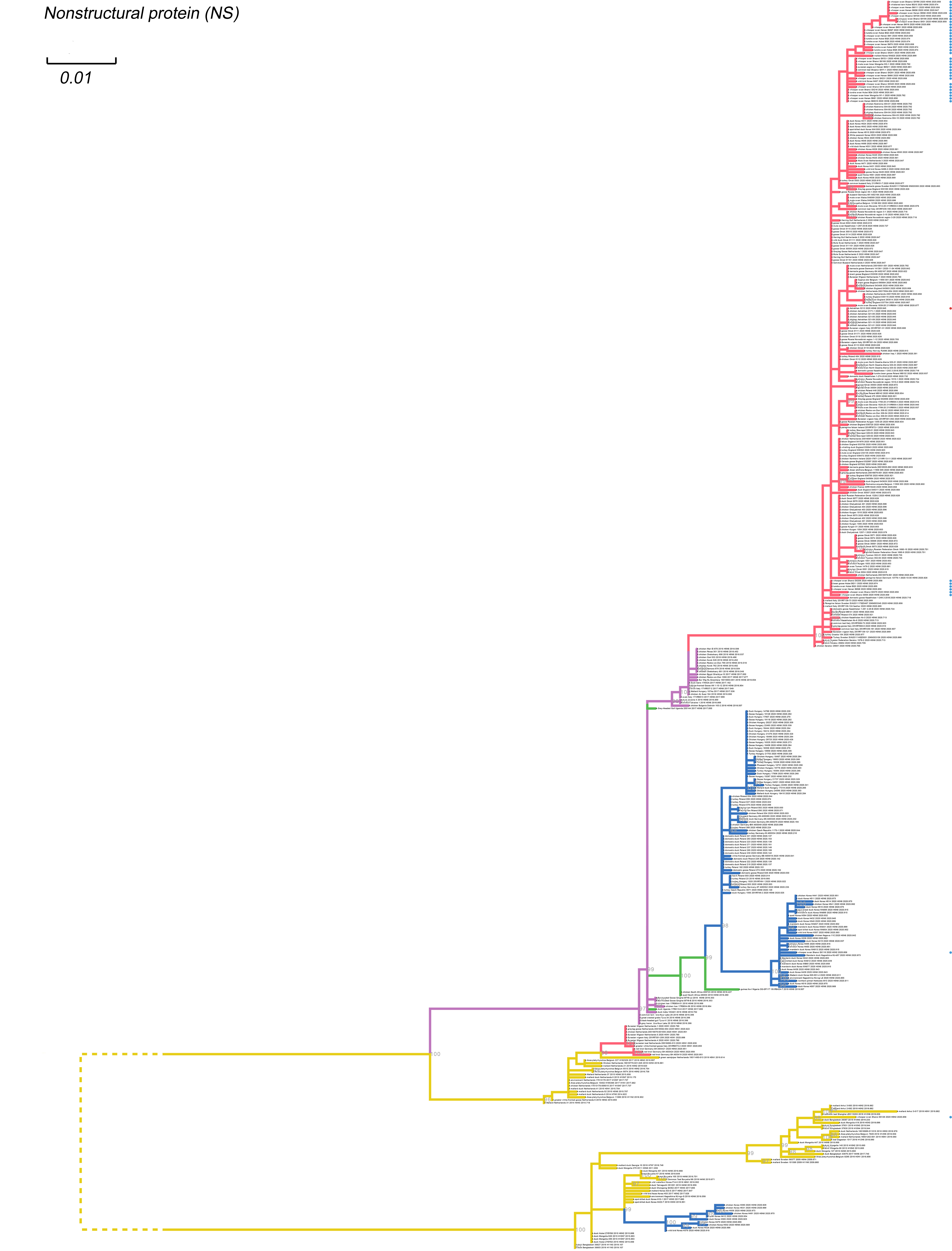

Supplement: Supplemental Material [file TEMI_A_2011622_SM3732.zip › Supplmentary files/Appendix Figure 1-NS-1000.jpg]

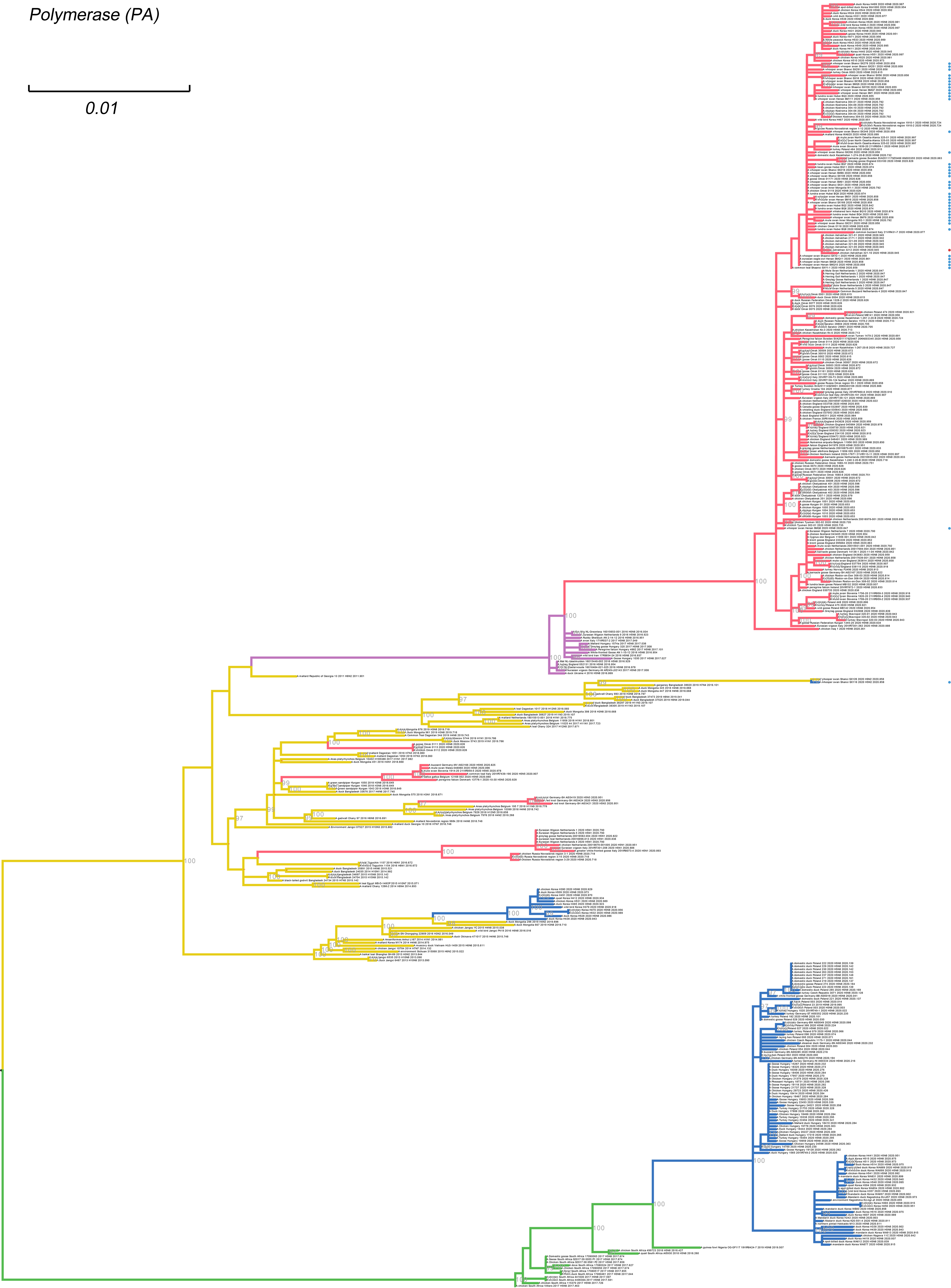

Supplement: Supplemental Material [file TEMI_A_2011622_SM3732.zip › Supplmentary files/Appendix Figure 1-PA-1000.jpg]

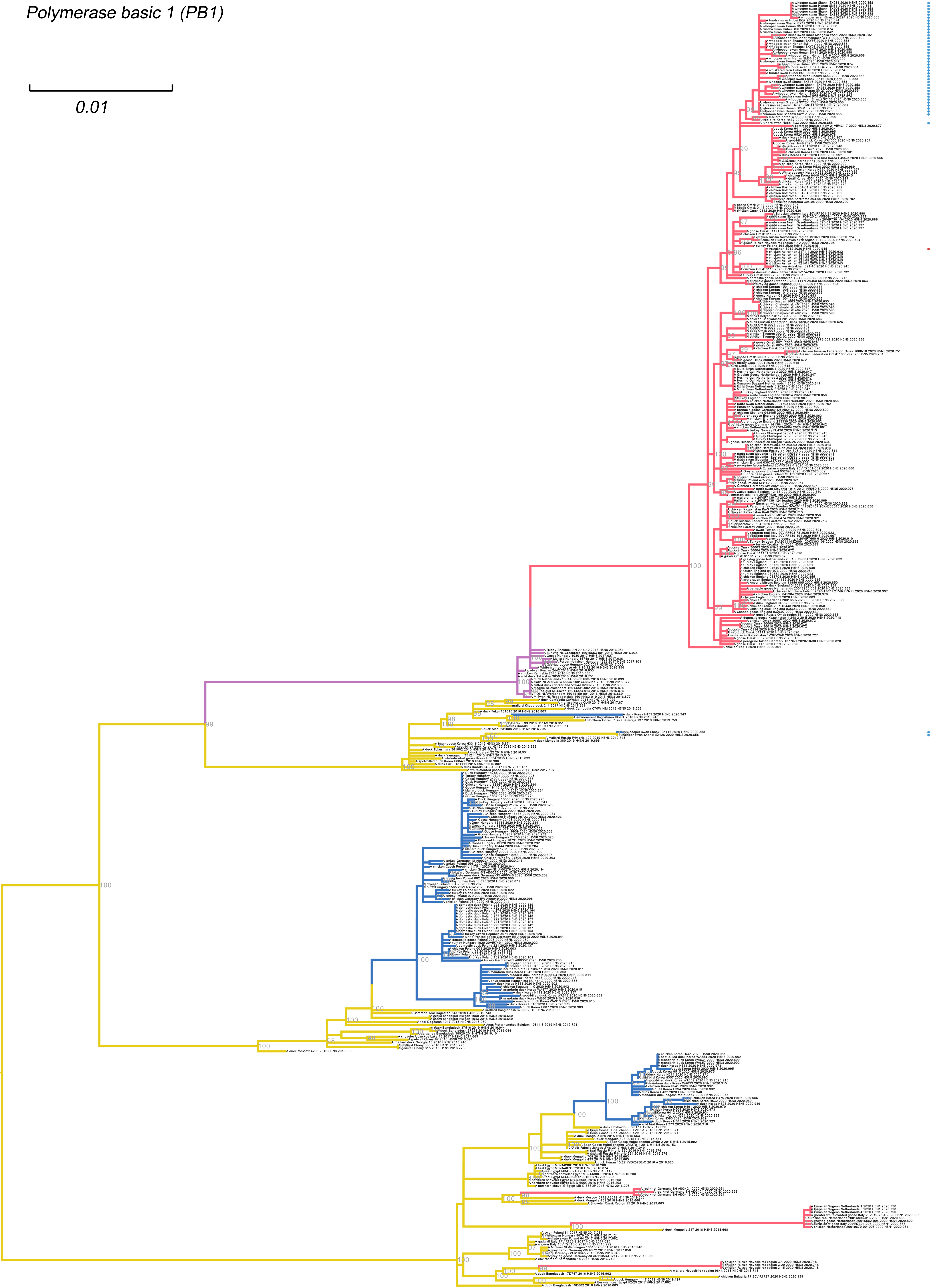

Supplement: Supplemental Material [file TEMI_A_2011622_SM3732.zip › Supplmentary files/Appendix Figure 1-PB1-1000.jpg]

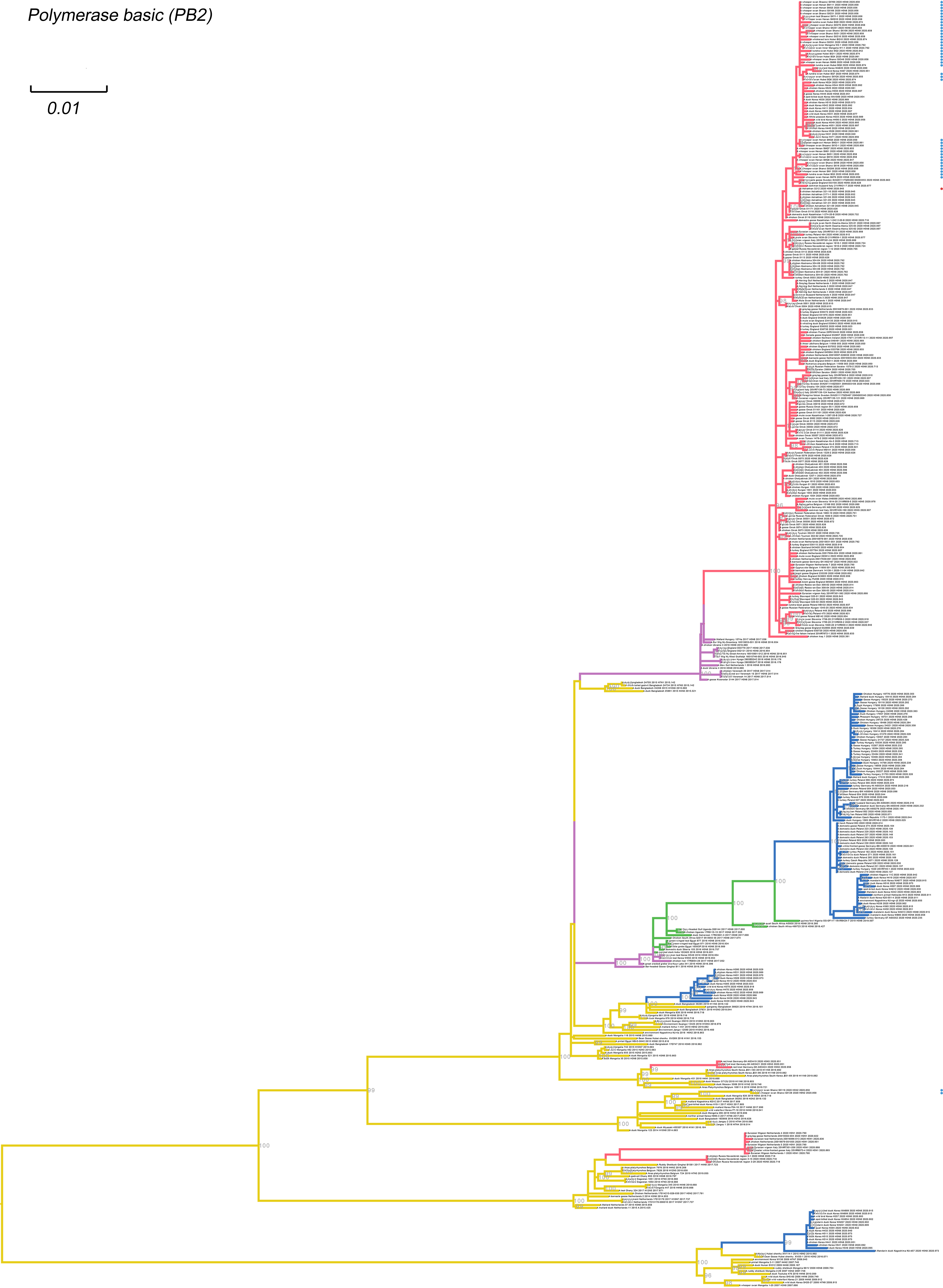

Supplement: Supplemental Material [file TEMI_A_2011622_SM3732.zip › Supplmentary files/Appendix Figure 1-PB2-1000.jpg]
